# Whole-exome and transcriptome sequencing of refractory diffuse large B-cell lymphoma

**DOI:** 10.18632/oncotarget.13239

**Published:** 2016-11-09

**Authors:** Ha Young Park, Seung-Bok Lee, Hae-Yong Yoo, Seok-Jin Kim, Won-Seog Kim, Jong-Il Kim, Young-Hyeh Ko

**Affiliations:** ^1^ Department of Biomedical Sciences, Seoul National University Graduate School, Seoul, 03080, Republic of Korea; ^2^ Department of Pathology, Samsung Medical Center, SungKyunKwan University, Seoul, 06351, Republic of Korea; ^3^ Department of Pediatrics, Seoul National University College of Medicine, Seoul National University Children's Hospital, Seoul, 03080, Republic of Korea; ^4^ Department of Health Sciences and Technology, Samsung Advanced Institute for Health Sciences and Technology, Sungkyunkwan University, Seoul, 06351, Republic of Korea; ^5^ Section of Hematology-Oncology, Department of Medicine, Samsung Medical Center, Sung Kyun Kwan University, 06351, Republic of Korea

**Keywords:** diffuse large B cell lymphoma, refractory, whole exome, transcriptome, sequencing

## Abstract

Diffuse large B-cell lymphoma (DLBCL) is the most common type of non-Hodgkin lymphoma. Although rituximab therapy improves clinical outcome, some patients develop resistant DLBCL; however, the genetic alterations in these patients are not well documented. To identify the genetic background of refractory DLBCL, we conducted whole-exome sequencing and transcriptome sequencing for six patients with refractory and seven with responsive DLBCL. The average numbers of pathogenic somatic single nucleotide variants and indels in coding regions were 71 in refractory patients (range 28–120) and 38 (range 19–66) in responsive patients. Missense mutations of *TP53* were exclusive in 50% (3/6) of refractory patients and involved the DNA-binding domain of *TP53*. All missense mutations of *TP53* were accompanied by copy number deletions. *RAB11FIP5, PRKCB, PRDM15, FNBP4, AHR, CEP128, BRE, DHX16, MYO6*, and *NMT1* mutations were recurrent in refractory patients. *MYD88*, *B2M*, *SORCS3*, and *WDFY3* mutations were more frequent in refractory patients than in responsive patients. *REL*–*BCL11A* fusion was found in two refractory patients; one had both fusion and copy number gain. Recurrent copy gains of *POU2AF1, SLC1A4, REL11, FANCL, CACNA1D, TRRAP*, and *CUX1* with significantly increased average expression were found in refractory patients. The expression profile revealed enriched gene sets associated with treatment resistance, including oxidative phosphorylation and ATP-binding cassette transporters. In conclusion, this study integrated both genomic and transcriptomic alterations associated with refractory DLBCL and found several treatment-resistance alterations that may contribute to refractoriness.

## INTRODUCTION

Diffuse large B-cell lymphoma (DLBCL) comprises about 40% of non-Hodgkin lymphoma and is a heterogeneous disease in terms of the pathological changes, cell of origin (COO), clinical course, and genetic alterations. Clinically, DLBCL is a curable disease and 30–50% of patients can achieve a complete remission after first-line treatment with rituximab (R)–cyclophosphamide, doxorubicin, vincristine, and prednisone (CHOP). However, up to 40% of DLBCL patients experience disease relapse or are refractory to the initial treatment and have a poor chance of survival [1].

Many clinical, immunohistochemical, and molecular factors have been proposed for predicting the treatment response and prognosis of patients after standard R-CHOP treatment, but no reliable markers have been identified. The International Prognostic Index (IPI) is regarded as a surrogate marker of the biology of the host and tumor. The IPI is calculated using the patient's age >60 years, abnormal increase in LDH level, Ann Arbor stage III or IV disease, >1 extranodal site, and European Cooperative Oncology Group performance status ≥2. Although the IPI is an excellent prognostic marker, it fails to predict the prognosis and treatment response in many patients [2].

DLBCL is divided into germinal center B-cell-like (GCB) DLBCL and activated B-cell-like (ABC) DLBCL based on the gene expression profile or immunohistochemistry (IHC) [3, 4]. ABC-type DLBCL is characterized by constitutive activation of the NF-κB signaling pathway and has a poorer prognosis with standard R-CHOP chemotherapy compared with GCB-type DLBCL [5]. This difference is exploited therapeutically, and pathway-targeted therapy has been developed to improve the survival of patients with ABC-type DLBCL. In addition to these biological markers, other factors including expression and rearrangement of the BCL-2 gene, [6] expression and mutation of the p53 gene, [7] expression of B2 microglobulin protein, [8] and proliferation fraction [9] have been used to predict the prognosis of patients with DLBCL.

DLBCL is associated with alterations in many different genes, with a median somatic frequency of 3.3 mutations per Mb [10]. Identification of these genetic aberrations of DLBCL is needed to provide more rational molecularly defined approaches to treatment. Deep sequencing is currently the method of choice for cataloging genomic changes in tumors. To obtain a comprehensive overview of the gene expression patterns and genomic alterations in refractory DLBCL, we produced a multidimensional genomic dataset based on data obtained from whole-exome sequencing (WES), transcriptome sequencing, and copy number variation in six patients with refractory DLBCL and seven patients with DLBCL with a good treatment response.

## RESULTS

### Single nucleotide variants (SNVs) and insertion/deletions (indels)

**Figure 1:**
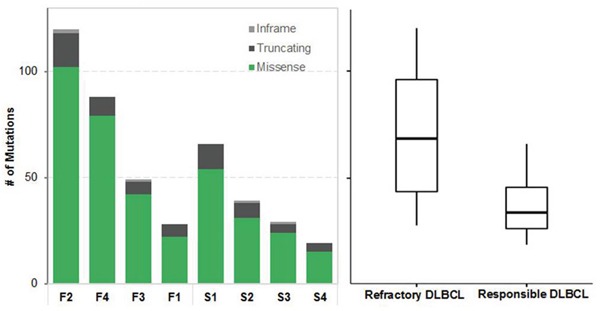
Summary of somatic single nucleotide variants (SNVs) Left, different colors indicate different types of nonsynonymous mutations. F respresents refractory DLBCL and S represents responsible DLBCL. Right, box plot compares number of SNVs of two groups.

We performed WES and RNA-Seq for all 13 samples: seven from treatment-responsive patients (S group) and six from treatment-refractory patients (F group). Eight tumor–normal pairs (S1–4, F1–4) were checked primarily and had high-confidence somatic mutations. The average numbers of pathogenic somatic SNVs and indels in coding regions were 38 (range 19–66) in the S group and 71 in the F group (range 28–120). These coding variants tended to occur more frequently in the F group, although the difference was not significant (*P* = 0.3429, Figure 1).

### Genes mutated more frequently in refractory DLBCL

**Figure 2:**
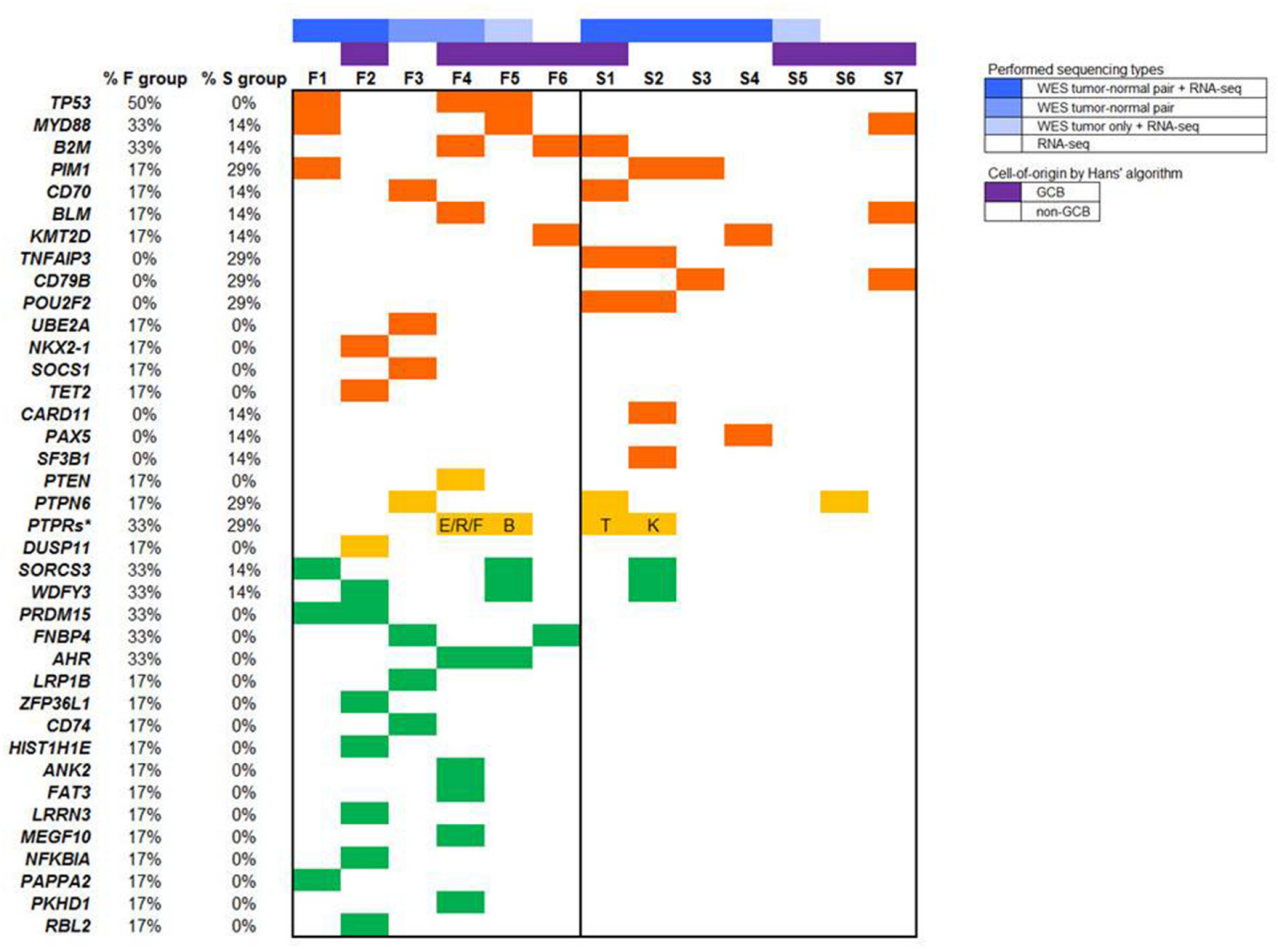
Mutation distribution Each column represents a DLBCL case. Top panel in different colors represent the sequencing performed for each case and cell-of-origin subtypes. Each row represents a gene which belongs to known DLBCL-associated genes or exclusively/more frequently mutated genes in refractory DLBCL. The asterisk represents a group of protein tyrosine phosphatases receptor type (PTPRs) family genes. Different isoforms are indicated by corresponding alphabet.

SNVs of *TP53* were the most frequent (50%) and exclusive to refractory DLBCL (Figure 2). All SNVs of *TP53* occurred in the DNA-binding domain (Supplementary Figure S1a–S1c). Variant allele frequencies of *TP53* were >60% at the DNA level and higher (>90%) when transcribed. *RAB11FIP5, PRKCB, PRDM15, FNBP4, AHR, CEP128, BRE, DHX16, MYO6*, and *NMT1* mutations were identified more than two samples in the F group (Supplement Table S4).

*MYD88, B2M, SORCS3*, and *WDFY3* mutations were found in two refractory cases and one responsible sample.(Figure 2). Activating hotspot mutations of *MYD88* (p.Leu265Pro) have been reported to be more common in non-GCB type DLBCLs [11]. All four mutations occurred in highly conserved loci in the Toll-IL-1 receptor (TIR) domain of *MYD88* (Supplementary Figure S1d). Two of four mutations in these patients occurred on the hotspot; the others involved different loci.

*B2M* encodes β2 microglobulin, which is a component of the class I major histocompatibility complex and is required for recognition by cytotoxic T cells. Inactivating mutations and deletions in *B2M* are common and account for up to 12% of DLBCLs [12]. The mutations of *B2M* in refractory DLBCL patients included a missense mutation (n=1) and frameshift deletion (n=1).

**Figure 3:**
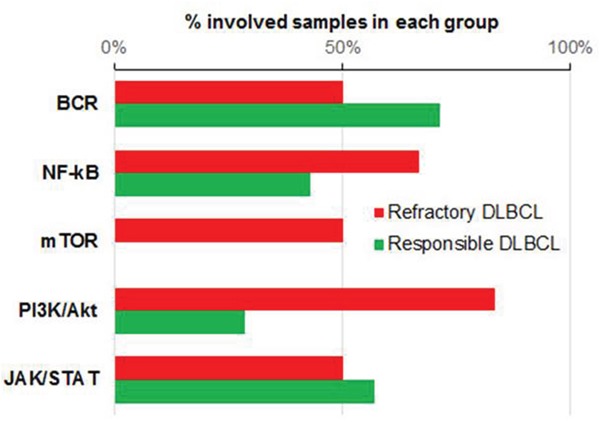
Mutational state of five major pathways % of involved samples in each pathway indicated by red bar for refractory DLBCL, green for responsible DLBCL. BCR, B-cell receptor signaling pathway.

We assessed the mutation distribution of the major pathways associated with the pathogenesis of DLBCL which including BCR signaling, NF-kB, mammalian target of rapamycin (mTOR), phosphoinositide 3-kinase (PI3K)-protein kinase B (Akt), and JAK/STAT pathways. These are closely related with each other. BCR activation is upstream of PI3K/Akt and NF-kB pathways [13]. mTOR pathway is activated by Akt which can activate NF-kB pathway [14]. NF-kB and STAT3 collboratively mediate tumorigenesis [15]. The F group showed more frequent mutations in the PI3K-Akt and mTOR pathways (Figure 3). *PTEN* and *TSC2* both belong to the PI3K–Akt and mTOR pathways and were mutated in F4. These are known tumor-suppressor genes (TSGs). All patients in the F group had ≥1 tumor-suppressor mutations, whereas TSG mutations were identified only in half of the patients in the S group.

### Common mutations in all DLBCL patients irrespective of treatment response

Of the 485 candidate SNVs and indels (Supplement Table S4), mutation of four genes (*TP53, MYD88, PIM1*, and *B2M*) that relate to the major driver genes in DLBCL [16, 17] were shared by three of 13 patients (23.1%). Most somatic SNVs or indels occurred in different positions, except for *MYD88*, which encodes p.Leu265Pro, and *PIM1*, which encodes p.Glu226Lys.

In addition, *PTPN6*, *TRIP12*, *SORCS3*, and *WDFY3* were mutated in three of the 13 patients (23.1%). *PTPN6* and *PTEN* belong to the Class I classical Cys-based phosphatase family. *PTPN6* encodes SHP-1 phosphatase, which attenuates BCR signaling by dephosphorylating the ITAM motifs of the CD79A and CD79B signaling subunits of the BCR [18]. All mutations identified in this study were missense variants (p.Leu63Gln, p.Ser26Asn, p.Lys68Thr, and p.Pro105Leu), and three-quarters of the *PTPN6* mutations involved the SH2 domain. One patient (S1) had both a missense mutation and copy-neutral loss of heterozygosity (CN-LOH, Supplementary Figure S3c), which suggested a loss of tumor-suppressor function. Loss-of-function mutations in *PTPN6* promote STAT3 deregulation via JAK3 kinase in DLBCL [19].

We checked the mutation of genes belonging to the Class I classical Cys-based phosphatase family and found an additional eight mutations in variable receptor type protein tyrosine phosphatase and one in *DUSP11*. A total of 12 mutations were found in 53.8% (7/13) of patients. Five of 12 mutations in the phosphatase family involved the phosphatase domain and two occurred in the fibronectin domain, which is located in the extracellular-interacting part. Frequent mutations involving functional domains implies that alterations in phosphatases may play an important role in the pathogenesis of DLBCL.

*SORCS3* encodes a type-I receptor transmembrane protein that is a member of the vacuolar protein sorting 10 receptor family. The function of *SORCS3* is not known, but a missense mutation was recently reported in 6% of relapsed/refractory DLBCL (rrDLBCL) patients [16, 20]. Thyroid hormone receptor interactor 12 (*TRIP12*), encoding HECT domain ubiquitin E3 ligase, is vital for the homeostasis of ubiquitin-controlled events after DNA breakage [21]. *WDFY* encodes a phosphatidylinositol 3-phosphate-binding protein that functions as a master conductor for aggregate clearance by autophagy [22]. Insertion or missense mutations have been reported in up to 21% of rrDLBCL patients [16].

### Fusion genes

To identify confident fusion transcripts, we used three different fusion callers. Eight were selected as candidates that were common in >2 fusion callers (Supplementary Table S5, Supplementary Figure S4). Five of eight were identified in two S patients and three in two F patients. We checked for differences in expression across breakpoints in each partner gene to predict functional alterations caused by fusion. Most of the partner genes did not show differences in fusion status; however, *REL*, *BCL11A*, and *MAPK13* showed distinct read-depth patterns across the breakpoints (Supplementary Figure S5). The fusion transcript of *PIM1–MAPK13* comprised each protein kinase domain of PIM1 and MAPK13. Both partner genes of *PIM1–MAPK13* were upregulated compared with nonfusion samples, on the assumption that fusion leads to gain of function of the involved genes. In the two patients in the F group with *REL–BCL11A* fusion, one had both fusion and copy number gain, and the same result was identified for *PIM1–MAPK13* fusion (Supplementary Figure S5). The S group exhibited known DLBCL-associated structural variations, such as translocation of *BCL6* and *IGLL5*, and inversion of *PRDM1*. Inactivation of *PRDM1* reported previously in DLBCL patients comprises mainly truncating mutations [23, 24]. We identified *PRDM1–ATG5* fusion transcript generated by inversion. The fusion transcript was joined out-of-frame, which may reflect a newly identified mechanism of *PRDM1* inactivation. *BCL6* and IG are commonly translocated in DLBCL, and we identified fusion transcripts of these genes in two samples from the S group.

### Copy number alterations (CNAs)

**Figure 4:**
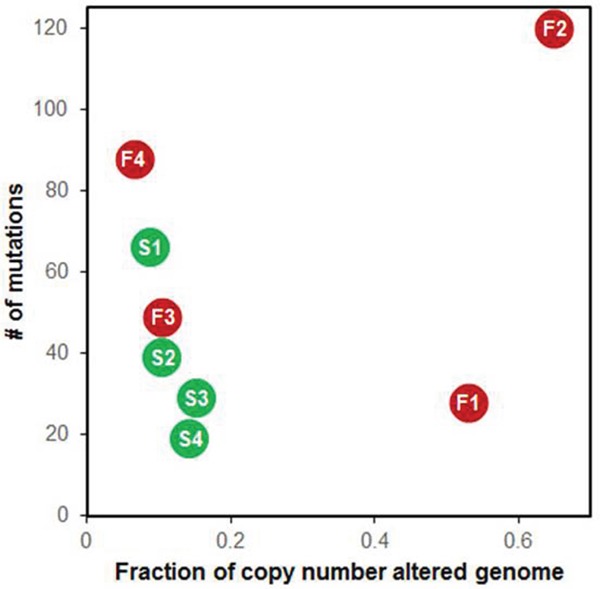
Relationship of somatic single nucleotide variants (SNVs) and copy number alterations F1-4 in red dots represent refractory DLBCL, S1-4 in green dots represent responsible DLBCL.

Segmental CNAs were defined based on a T/N coverage ratio >1.25 or <0.75. Segmental CNAs were found in >20% of genomes in the refractory DLBCL patients F1 and F2 (Figure 4 and Supplementary Figure S2). The minimal common regions (MCRs) of CNAs identified exclusively in refractory DLBCL are listed in Table 1. We checked the expression levels of the genes included in each MCR; the genes with concordant alterations between CNA and expression are listed in the column ‘Genes validated by RNA-seq’ in Table 1.

**Table 1:** Minimal common regions of copy number alterations exclusive in the refractory DLBCL

Chr	Start	End	Cytoband	Genes validated by RNA-seq	CNA type	F1	F2	F3	F4	F5
1	7792507	12726746	1p36.22–p36.23	*CLSTN1, H6PD, MTOR, UBE4B*	Loss		---			--
1	13940794	16776736	1p36.13–p36.21	*DDI2, SPEN*	Loss		--			--
1	32384570	35229325	1p34.3–p35.1	*LCK*	Loss		---	--		
2	58387242	68359137	2p15–p16.1	*BCL11A, PAPOLG, REL, SLC1A4, WDPCP*	Gain		++		+++	
3	50598347	56592938	3p21.2–p14.3	*CACNA1D*	Gain	++	++			
6	33169521	44275966	6p21.32–p21.2	*CNPY3, CUL9, LEMD2, MAPK13, MEA1, NFKBIE, PFDN6, PIM1, POLR1C, TAPBP*	Gain			++		+++
7	96653580	101957868	7q22.1–q21.3	*C7orf61, CLDN15, CUX1, GIGYF1, IFT22, MDSPD3, PILRB, TRRAP*	Gain	++	+++			+++
7	102950761	112472730	7q31.1–q22.2	*PMPCB, RINT1*	Gain		+++			+++
8	8866541	11710204	8p23.1	*MTMR9*	Loss			--		--
8	121243705	142190953	8q24.22–q24.3	*ZFAT*	Gain		+++			++
11	1256532	3390732	11p15.5–p15.4		Loss	--		--		
11	9761727	43364203	11p14.1–p12		Gain		++		++	
11	92623656	115375283	11q22.2–q21	*POU2AF1*	Gain	++			+++	++
15	45335454	48059712	15q21.1		Loss			---	--	
15	56974451	63089601	15q22.2–q21.3	*RORA, VPS13C*	Loss		--		--	
15	63092566	65257801	15q22.31–q22.2	*SNX1*	Loss		--	--	--	

+++, tumor/normal (T/N) copy ratio > 1.5; ++, T/N copy ratio > 1.25 and ≤ 1.5; -- T/N copy ratio < 0.75 and ≥ 0.5; ---, T/N copy ratio < 0.5

### CNAs found in refractory DLBCL

Gain of *POU2AF1* was found in F1, F4, and F5 (Supplementary Figure S3). POU2AF1 protein, often called Oct-binding factor 1 (OBF-1), Oct coactivator from B cells (OCA-B), or BOB.1, is a transcriptional coactivator that is involved in transcription of immunoglobulin genes [25] and plays a role in B-cell development and Ig expression [26, 27]. Growth-promoting effects of overexpressed POU2AF1 have been demonstrated in human multiple myeloma cells from hematological malignancies [28]. In Hodgkin lymphoma, the percentage of relapsed patients after complete remission was greater in BOB.1-positive patients, and the association was stronger when BOB.1 expression was greater [29].

Gain of *BCL11A, REL, XPO1*, and *FANCL* at 2p16.3–p15 was found in two of five refractory *DLBCL* patients. Fusion or gain of *REL*, a member of the NF-κB pathway, and *BCL11A* was reported to be enriched in transformed lymphoma, and this may be a genomic marker for disease progression to clinically more aggressive forms [30, 31]. *XPO1* encodes CRM1, an exporter of several tumor suppressor proteins. Cytoplasmic export of tumor-suppressor proteins renders them inactive, which indicates that *XPO1* acts as a proto-oncogene. *XPO1* mutations were reported to be significantly overrepresented in a relapsed/refractory DLBCL patient cohort and in patients with mediastinal large B-cell lymphoma [20, 32]. Gain of *CACNA1D* at 3p14.3–p21.1 is recurrent in refractory DLBCL patients. *CACNA1D* encodes the L-type voltage-gated calcium channel. In the refractory DLBCL patients in our study, CACNA1D was overexpressed compared with the responsive DLBCL patients. Gain of transformation/transcription domain-associated protein (TRRAP) and *CUX1* at 7q22.1–q21.3 was present in three patients with refractory DLBCL. As a common component of many HAT complexes, TRRAP is an essential cofactor for both the c-Myc and E1A/E2F oncogenic transcription factor pathways [33, 34]. CUT-like homeobox 1 (*CUX1*) is a homeobox gene that is implicated in both tumor suppression and progression. Increased *CUX1* expression is associated with tumor progression [35]. RNA-seq data showed significantly increased average gene expression for *POU2AF1*, *SLC1A4*, *REL11*, *FANCL*, *CACNA1D*, *TRRAP*, and *CUX1* in the F group compared with the S group. Four of five patients in the F group showed a T/N copy ratio ≤0.5 for *TP53* (Supplementary Figure S1); this is a CNA that is consistent with one copy deletion.

### Gene expression signature in refractory DLBCL patients

We identified the genes expressed differently between the F and S groups. Using the criteria of log_2_FC ≥1 and FDR <0.05, we identified a total of 1531 differentially expressed genes (DEGs), including 744 upregulated and 787 downregulated genes. Upregulated DEGs were enriched in the canonical pathway gene sets associated with transcription/translation, replication/cell cycle/DNA repair, lipid synthesis, cellular energy metabolism, B cell receptor signaling, and Notch signaling (Supplementary Figure S6 and Table S6). Most significantly enriched gene sets were in the ‘transcription/translation’ group, which includes protein synthesis and degradation, genes encoding S and L ribosomal proteins, RNA polymerase subunits, zinc finger proteins, and exosome components. The ‘replication/cell cycle’ group included *E2F1* transcription activator, MCM7 possessing DNA helicase activity, telomerase reverse transcriptase (*TERT*), and centromere proteins. The second largest group was ‘cellular energy metabolism’, which included members of the NADH: ubiquinone oxidoreductase, ubiquinol–cytochrome C reductase complex as well as adenosine triphosphate (ATP) synthase components.

Downregulated DEGs were enriched in gene sets related to the immune response, especially T-cell receptor signaling, cytokine/chemokine signaling, and the complement cascade. In 2005, a study using whole genome microarray suggested that there are three discrete subsets of DLBCL [36]: ‘oxidative phosphorylation (OxPhos)’, ‘B cell receptor/proliferation (BCR/proliferation)’, and ‘host response (HR)’. We extracted the genes classifying the three groups (‘Consensus Clusters Markers’) to determine which group refractory DLBCL belongs to. The log_2_FC of these genes differed significantly between the three groups (*P* = 5.475e–09). The median log_2_FC was highest in the OxPhos group and lowest in the HR group (Supplementary Figure S7); the refractory DLBCL group had a higher expression of OxPhos genes and lower expression of HR gene compared with the responsive group.

We identified cancer outlier genes (COGs) and checked the overrepresented gene sets in these COGs. The top-ranked gene sets overlapped with many of the upregulated gene sets. Additionally identified genes included the ATP-binding cassette transporter gene family. (Supplementary Table S7) Three of four refractory samples overexpressed at least one ATP-binding cassette transporter, while one of six responsible sample did. (Supplementary Figure S8) Major signaling pathways, such as the mitogen-activated protein kinase (MAPK), insulin, adipocytokine, Toll-like receptor, mTOR, and VEGF pathways, were significantly enriched (FDR <0.05, Supplementary Table S7). *MAPK12* and *MAPK13*, which encode p38 gamma and delta, respectively, are members of the MAPK family and the p38 signaling pathway is involved in resistance to cytotoxic drugs and its inhibition by rituximab sensitizes cells to drug-induced apoptosis [38]. A recent study reported that the p38 expression is increased in CHOP-resistant patients [39]. Insulin, adipocytokine, and Toll-like receptor pathways, which share the PI3K–Akt pathway, were overexpressed in the F group. Taken together with the mutation results, these findings suggest that activation of the PI3K–Akt–mTOR pathway may be important to treatment resistance in DLBCL.

### Difference in genetic alterations between cell-of-origin (COO) groups

We checked known DLBCL-associated genetic alterations and compared difference between COO groups. In a total of 13 cases, 8 were GCB and 6 were non-GCB type irrespective of treatment response subtypes. It is well known that genes frequently mutated in non-GCB type are *CARD11, MYD88, CDKN2A, CD79A/B TNFAIP3*, and *PRDM1* while in GCB type, *GNA13, EZH2, BCL6* mutations are frequent. *TP53, B2M, MEF2B* and *CREBBP* mutations can be are found in both types [39]. In our study, 8 GCB cases showed mutations of *MYD88*(2/8), truncating mutations of TNFAIP3 (1/8), missense mutations of *CD79B* (1/8), *B2M*(3/8), and *TP53*(2/8) while 6 non-GCB cases had mutations of *MYD88*(1/6), truncating mutations of *TNFAIP3* (1/6), missense mutations of *CD79B* (1/6), non-frameshift deletion of *CARD11*(1/6), *MEF2B*(2/6), and *TP53*(1/6). *GNA13, EZH2* and *BCL6* mutations were not found in both types. These results didn't show distinct mutations profile as previous reports, which might be due to small sample size.

In GCB subtype, copy gain of *REL*, and *BCL11A* were found in two cases and copy neutral LOH of *PTEN* was found in a case. In non-GCB subtype, copy gain of *BCL2* was found in four cases, and copy loss of *CDKN2A* and *CDKN2B* were found in two different cases. These findings are in line with those of previous report [40]. Of the 11 samples conducted RNA-seq, up-regulted DEGs were enriched in gene sets of extracellular matrix proteins in non-GCB type and gene sets associated with transcription/translation in GCB type.

## DISCUSSION

In the present study, we used exome and transcriptome sequencing to identify the molecular background of refractory DLBCL by comparing two groups of DLBCL patients with different treatment responses. Although the sample size was rather small to make conclusion, we have discovered putative sources of resistance to R-CHOP therapy. These include overexpression of ATP-binding cassette transporter genes, mutations or CNAs related to cellular proliferation and apoptosis, activation of the PI3K–AKT–mTOR pathway, and increased mitochondrial oxidative phosphorylation.

ATP-binding cassette transporters are a family of transporter proteins that contribute to drug resistance via ATP-dependent drug efflux pumps. These transporters are expressed on the cell membrane and transport their substrates across the membrane in an ATP-dependent manner. The overexpression of ATP-binding cassette transporters reduces the intracellular concentration of the substrate agents, including vincristine and doxorubicin, commonly used in the treatment of lymphoma patients [41]. In addition, ATP-binding cassette transporters in aggressive lymphoma can modulate exosome release, which leads to exosome-mediated shielding of target cells as a critical determinant of tumor cell susceptibility to antibody therapy [42]. In this study, the ATP-binding cassette transporters, *ABCA3*, *ABCB7*, *ABCC1*, *ABCB1*, *ABCG2*, and *ABCG1* were overexpressed in the F-group. *ABCB1*, also called MDR1, is the prototype of ATP-binding cassette transporters and is overexpressed in up to 80% of relapsed lymphomas [43, 44].

Oncogenic signaling pathways can drive metabolic reprogramming, and metabolic adaptation is a mechanism of resistance to targeted therapy [45]. Refractory DLBCL show DEGs that were significantly enriched in genes involved in mitochondrial oxidative phosphorylation, including *UQCR11*, *COX7C*, *UQCRQ*, *NDUFB9*, *NDUFB10*, *NDUFC1*, *ATP5D*, *ATP5O*, *ATP5G2*, and *ATP5J2*. DLBCL is a heterogeneous disease and can be subdivided into B-cell receptor, OxPhos, and host response tumors based on the transcriptional profile [36]. OxPhos DLBCL cells harbor the signature of genes involved in mitochondrial metabolism. In contrast to the B-cell receptor subtype, OxPhos DLBCLs do not display active or functional BCR signaling and are insensitive to inhibitors of BCR signaling [46]. They are selectively sensitive to pharmacological or genetic inhibition of fatty acid oxidation, which suggests that the metabolic features of this subtype may be exploited therapeutically [47].

Many chemotherapeutic agents exert antitumor effects by inducing apoptosis in tumor cells, and some alterations in apoptosis-signaling pathways are associated with drug resistance. TP53 was the most frequent mutation found in our study and was exclusive to the patients with refractory DLBCL. Half of the F group (3/6) had nsSNVs in the DNA-binding domain of TP53. Each nsSNV was accompanied by a deletion, which resulted in LOH and transcription of altered mRNA. Abnormalities in the tumor-suppressor gene, p53, have also been shown to be associated with drug resistance and short progression-free survival in patients with non-Hodgkin lymphoma [48]. In DLBCL, *TP53* mutations have been reported as a marker of poor survival [7, 49, 50]. A recent study of 506 DLBCL patients showed that *TP53* mutation was a predictor of survival in R-CHOP-treated patients, whereas TP53 deletion and loss of heterozygosity did not confer worse survival [7]. The *TP53* mutation rates in these studies were about 20%, which is similar to the rate observed in our study (23.1%, 3/13). Studies of refractory or recurrent DLBCL using NGS platforms have been published recently, and the reported mutation rates of *TP53* were lower than the 50% observed in our study: 32%, [20] 14.9% (32/215), [32] and 21.4% (3/14) [16]).

In this study, activation of the PI3K–Akt–mTOR pathway was predominant in the refractory DLBCL patients. The PI3K–Akt–mTOR signaling pathway plays an important role in controlling proliferation and survival of tumor cells in various types of malignancy, including DLBCL [51]. Activation of the PI3K–Akt–mTOR pathway was reported to be related to poor disease outcome in DLBCL patients treated with CHOP but not in those treated with R-CHOP [52]. In our study, refractory DLBCL patients with an altered mTOR pathway showed a poor response despite R–CHOP treatment. The number of DLBCL patients with refractory disease requires further searching for novel drugs to overcome cell resistance. Agents that directly target the PI3K–Akt–mTOR pathway have potential for the development of future treatments both as single agents and in combination with standard chemotherapeutics [51].

In conclusion, we explored the genetic characteristics of refractory DLBCL and found mutation of genes involved in proliferation and apoptosis, overexpression of drug-resistant genes, metabolic reprogramming with activated mitochondrial oxidative phosphorylation, and abnormal activation of signaling pathways. Although these results must be validated in a larger numbers of samples, our results provide information that may be useful in developing therapeutic strategies for refractory DLBCL.

## MATERIALS AND METHODS

### Study subjects and sample preparation for next-generation sequencing (NGS)

To identify genomic alterations associated with a therapeutic response in DLBCL patients, we collected fresh-frozen tumor tissues obtained for initial biopsy from 13 patients treated with R-CHOP. Frozen sections were obtained to evaluate the cellularity, and samples with >50% of the tumor cells were included. Patients whose complete remission was maintained for >1 year were classified as the responsive group (S group) and the others were classified as the refractory (F group). Seven of the 13 tumor samples were from the S group (numbered S1–7) and six were from the F group (numbered F1–6). We also obtained blood samples from four patients in each group (NS1–4 and NF1–4), which were paired with the tumor tissues S1–4 and F1–4, respectively. DNA was extracted from all 13 tumors and eight paired blood samples, and RNA was extracted from the 13 tumor samples for deep sequencing. COO subtypes were determined according to Han's classification by IHC of CD10, Bcl-6, and MUM-1. All samples were negative for EBER in situ hybridization. The clinical information and study platforms applied to each sample are described in Supplementary Table S1. This study was approved by the institutional review board and was in accordance with the Declaration of Helsinki (Approval number: 2015-01-034-001).

### Exome and transcriptome sequencing and sequence alignment

We performed WES on 10 tumor samples (S1–5 and F1–5) and eight paired normal blood samples (NS1–4 and NF1–4). To enrich the coding regions, we used SureSelect Human All Exon 50M (Agilent Technologies) for tumors and SureSelect Human All Exon V4 for normal samples. Sequence reads were produced using an Illumina HiSeq2000 instrument with a median on-target depth of 53× for tumor samples (range 47–77×) and 102× (range 96–106×) for normal samples (Supplementary Table S2). We performed the alignment using BWA [53] with the default parameters and hg19 as the reference genome.

RNA-seq was also performed on samples from the 13 tumors (S1–7 and F1–6). Disposable RNA chips (Agilent RNA 6000 Nano LabChip kit) were used to determine the concentration and purity/integrity of RNA samples using an Agilent 2100 bioanalyzer. The sequencing libraries were prepared as previously described [54]. Raw reads from the Illumina HiSeq 2000 were aligned to the human reference genome (hg19) using the STAR [55] 2-pass method. Alignment performance was assessed using RNA-SeqQC (Supplementary Table S3), [56] and two samples (F3-4) with low throughput (# of mapped reads <10 Mb) were excluded from downstream analysis.

### Sequence variation analysis

PCR duplicates were removed using Picard (http://broadinstitute.github.io/picard/). A Genome Analysis Tool Kit (GATK) was used for indel realignment and base quality score recalibration. SNVs were identified using MuTect [57]. Somatic indels of eight tumor–normal pairs were called using GATK SomaticIndelDetector (https://www.broadinstitute.org/cancer/cga/indelocator), and indels of unpaired samples were predicted using GATK UnifiedGenotyper and HaplotypeCaller. Variants annotations were obtained using the software tool ANNOVAR [58], which integrated the results into databases on gene, type of variants, and minor allele frequency (MAF) in Exome Aggregation Consortium Version 0.3 (ExAC03, http://exac.broadinstitute.org) and Combined Annotation Dependent Depletion (CADD) score [59].

After listing the nonsynonymous SNVs (nsSNVs), splice site SNVs, and indels of the coding regions from eight paired samples, we applied the criteria and filters to identify candidate mutations as follows: 1) tumor allele frequency ≥10% and normal allele frequency ≤1%; 2) rare mutations based on MAF <0.5% in ExAC03; and 3) deleterious mutations based on a CADD score ≥20. We checked additional mutations of somatic candidate genes in unpaired samples. In samples with both DNA and RNA available, the mutations were checked if they were transcribed or edited in RNA. A full list of mutations is presented in Supplementary Table S4.

### Copy number alteration analysis

Somatic copy number alterations (CNAs) were identified in eight tumor–normal pairs using Excavator [60] in somatic mode. For two unpaired samples (S5 and F5), we used Excavator in pooling mode to compare each tumor sample with a mean of the eight normal (NS1–4 and NF1–4) samples. Excavator generates CNA calls based on the ratio of tumor–normal read counts (T/N ratio) and detects segments with a similar copy number. T/N ratios >1.25 and <0.75 were defined as copy gain and loss, respectively. When the T/N ratio was >1.5 or <0.5, the segments were regarded as high-level gain and deep loss, respectively. Minimal common regions (MCRs)[61] were identified using Integrative Genome Browser (IGV) [62]. For MCRs exclusive to F group, we checked the CNAs of individual genes. B allele frequency was calculated from alternate allele frequency of MuTect results and was used to identify CN-LOH.

### Fusion gene analysis

To identify fusion transcripts, we used deFuse [63], ChimeraScan [64], and pyPRADA [65]. We first excluded paralog pairs based on Ensembl version 75. Of the remaining results, those supported by ≥2 junction-spanning reads and that were predicted at least two of three tools were selected as fusion candidates.

### Differential expression of genes in the two groups

HTSeq [66] was used to count sequence reads for the genes. Differential expression was calculated using DESeq2 [67]. DEGs were defined as genes with a q-value <0.05 and log_2_(fold change, FC) ≥1. To identify overrepresented gene sets, we used MSigDB (http://software.broadinstitute.org/gsea/msigdb/index.jsp), which integrates gene oncology (GO) terms and KEGG pathways. To identify cancer outlier genes (COGs), we used the criteria suggested previously [68].

## SUPPLEMENTARY MATERIALS FIGURES AND TABLES




