# Supplementary material for: Whole-exome and transcriptome sequencing of refractory diffuse large B-cell lymphoma
Source: Oncotarget. 2016 Nov 9;7(52):86433–45. doi: 10.18632/oncotarget.13239 (PMC5349924; doi:10.18632/oncotarget.13239)
Supplement: Supplementary file 1 [file oncotarget-07-86433-s001.pdf]

Whole-exome and transcriptome sequencing of refractory diffuse large B-cell lymphoma

SUPPLEMENTARY FIGURES AND TABLES

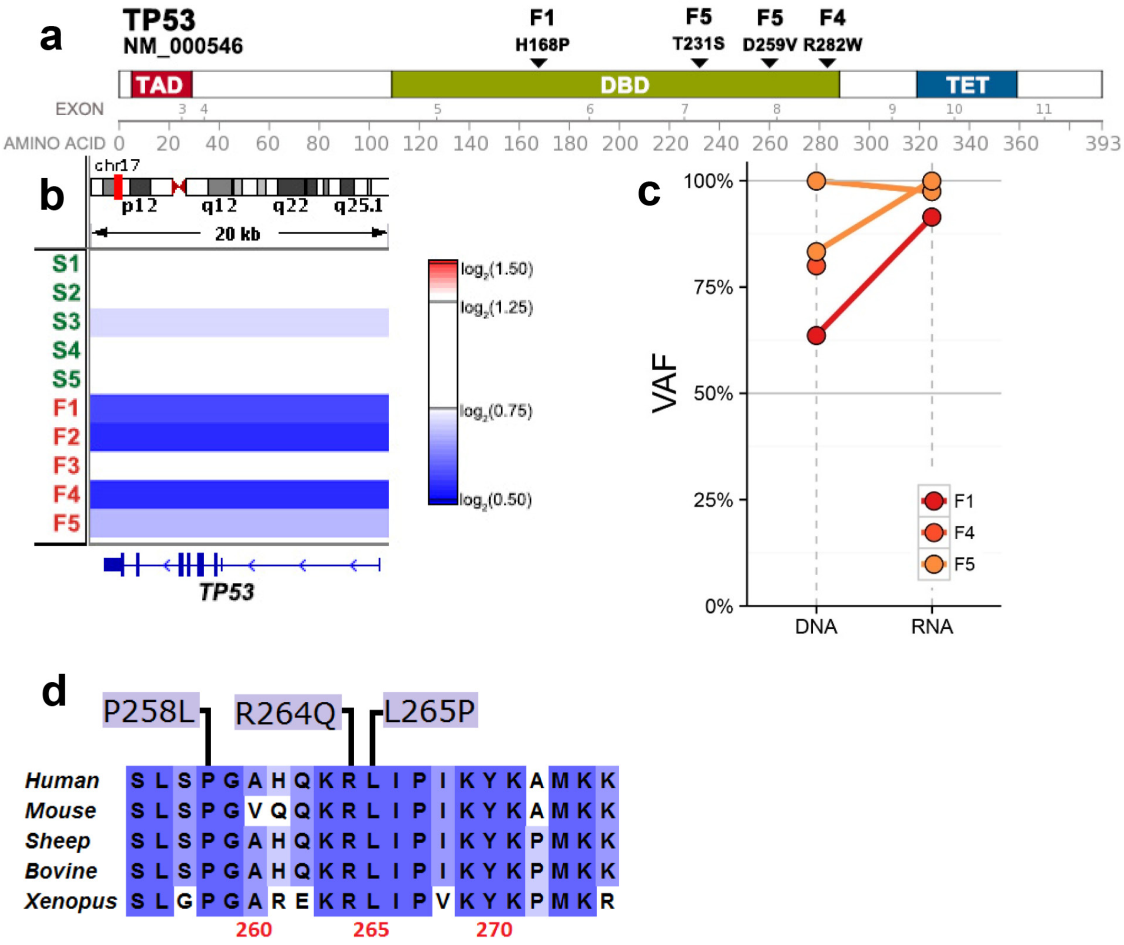

Supplementary Figure S1: Recurrent mutations of *TP53* and *MYD88*. a. Localization of altered residues in *TP53*. **b.** *TP53* copy number data is shown using IGV. Each row represents a DLBCL case. Blue bars indicate copy loss, the darker bar indicates higher copy loss level. Responsible cases are arrayed upper and refractory lower. **c.** Comparison of variant allele frequencies (VAFs) of *TP53* in DNA and RNA. Each line represents the relationship of one VAF of mutation in comparison. **d.** The evolutionary conservation of the mutated resides of *MYD88*.

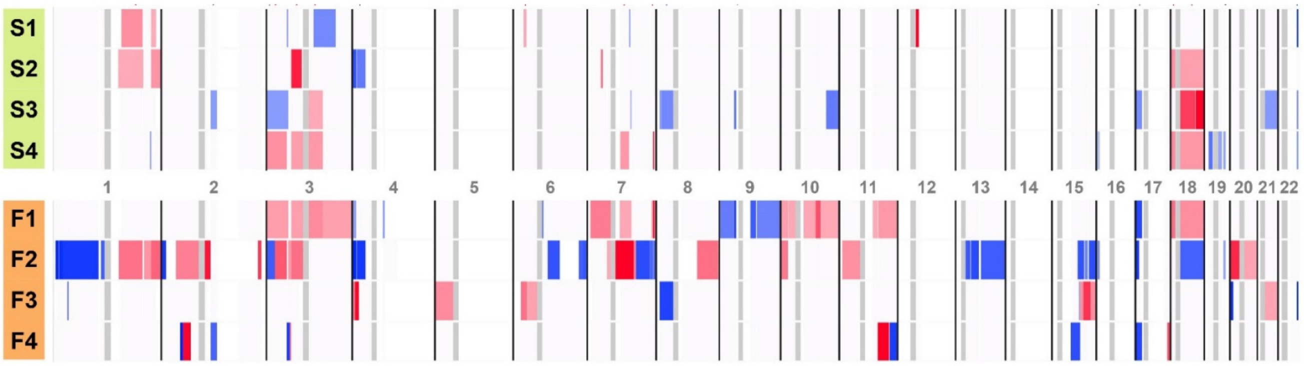

Supplementary Figure S2: Genome-wide distribution of somatic CNAs. Chromosome positions are indicated along x-axis with chromosomal boundaries indicated by black lines, centromere positions by grey bars. Copy gain are represented in red and loss in blue.

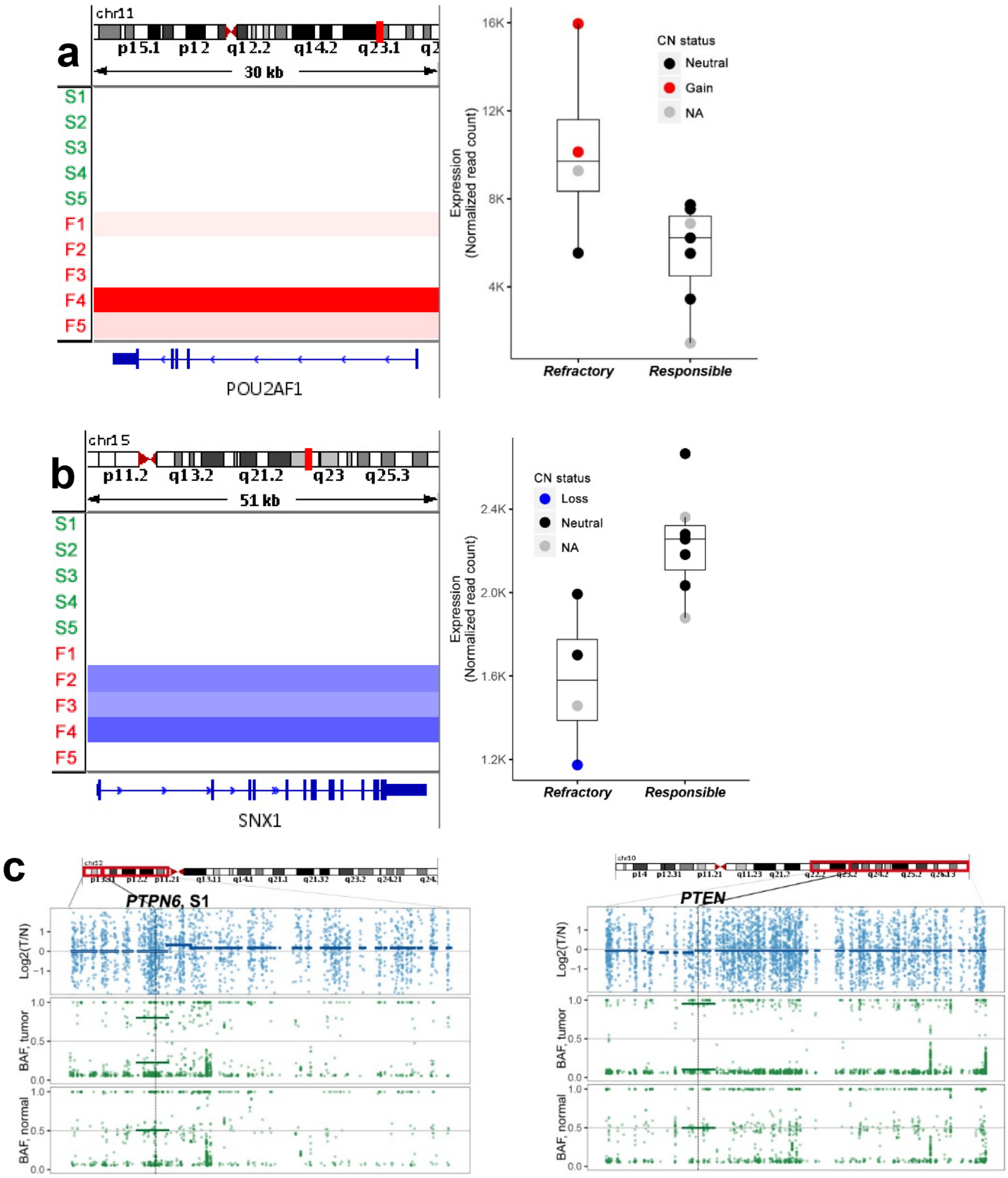

Supplementary Figure S3: Representative copy number alterations (CNAs). a-b. Left, CNAs are visualized using IGV, red bars represent copy gain and blue bars copy loss, the darker bar indicates higher copy alteration level. Right, comparison of expression level between two groups. Each dot with different color indicates copy number state, gain in red dots, loss in blue, neutral in black and not available (NA) in grey. Figure **a.** shows copy gain and concordant high expression level of POU2AF1. **b.** shows copy loss and concordant low expression level of SNX1. **c.** Copy neutral loss of heterozygosities (CN-LOHs) of *PTPN6* (left) and *PTEN* (right). Panels represent log2(tumor/normal) copy ratio, B allele frequency (BAF) of tumor, and BAF of normal from the top, respectively.

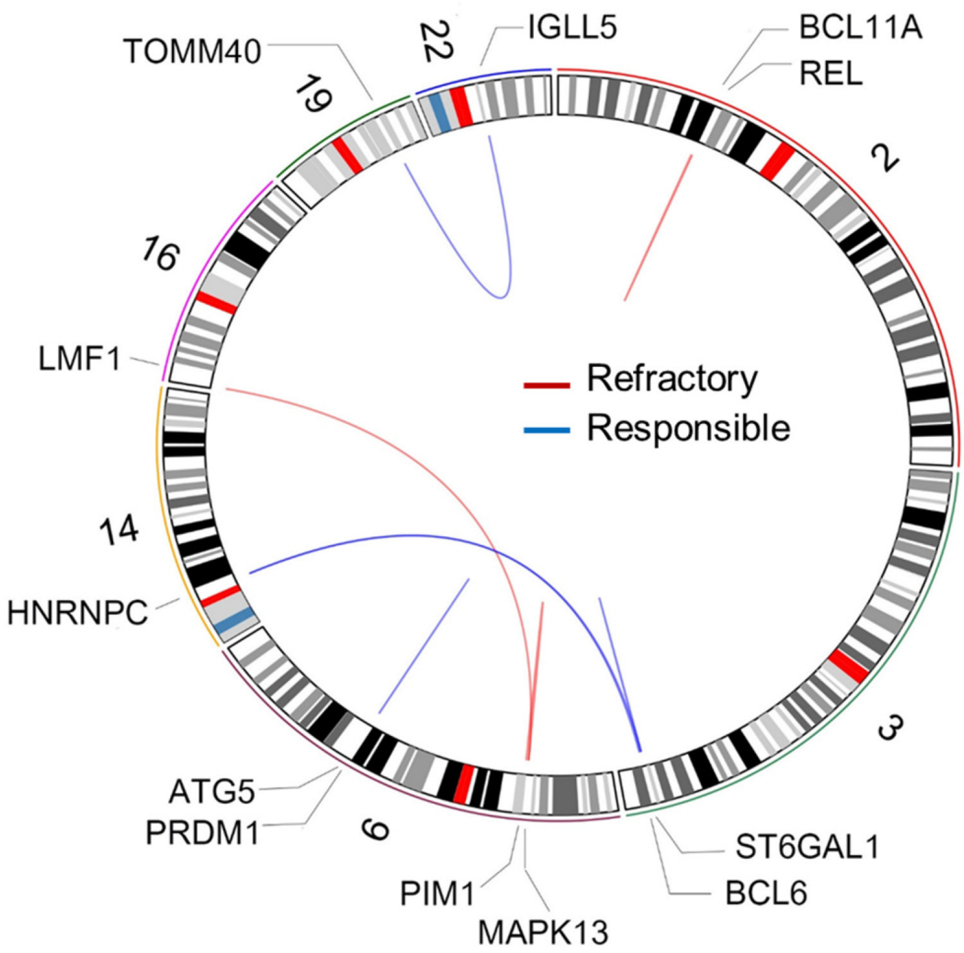

Supplementary Figure S4: Circos plot of fusion gene candidates.

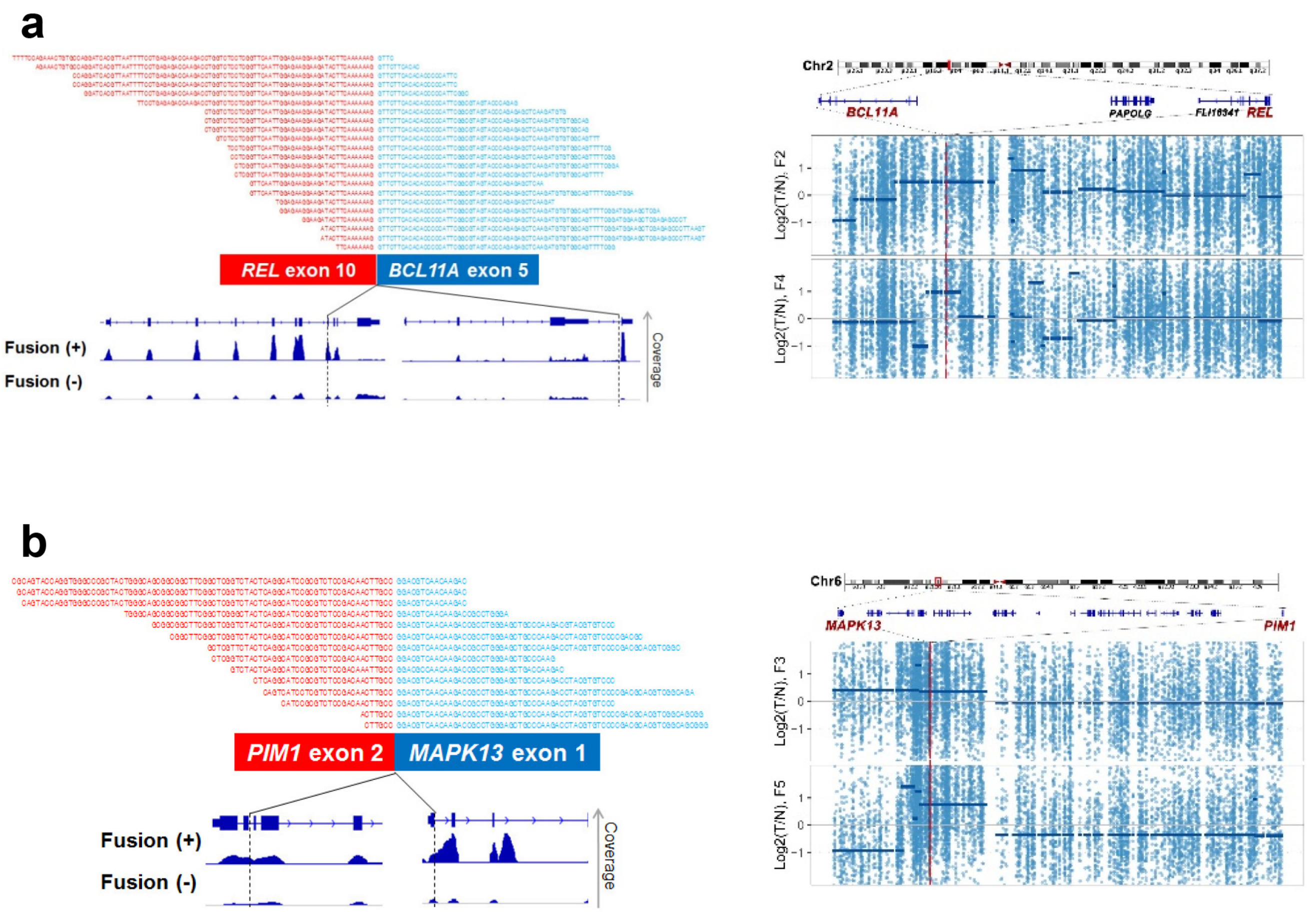

Supplementary Figure S5: Representative fusion gene candidates. a. Left, split reads are aligned on the breakpoint. *REL* sequences in red and *BCL11A* in blue. Expression levels across break point (dashed line) are shown at the bottom, comparing fusion-positive (F2) and –negative (F6) cases. Right, The log2 ratio of normalized read count between tumor and normal is indicated for all targeted exons (blue dots) of chr2 for F2 and F4. Segmental copy number change is indicated by blue line. Copy gain region of *REL* and *BCL11A* is indicated by a red dashed line. **b.** Left, split reads are aligned on the breakpoint. *PIM1* sequences in red and *MAPK13* in blue. Expression levels across break point (dashed line) are shown at the bottom, comparing fusion-positive (F5) and –negative (F6) cases. Right, The log2 ratio of normalized read count between tumor and normal is indicated for all targeted exons (blue dots) of chr2 for F3 and F5. Segmental copy number change is indicated by blue line. Copy gain region of *REL* and *BCL11A* is indicated by a red dashed line.

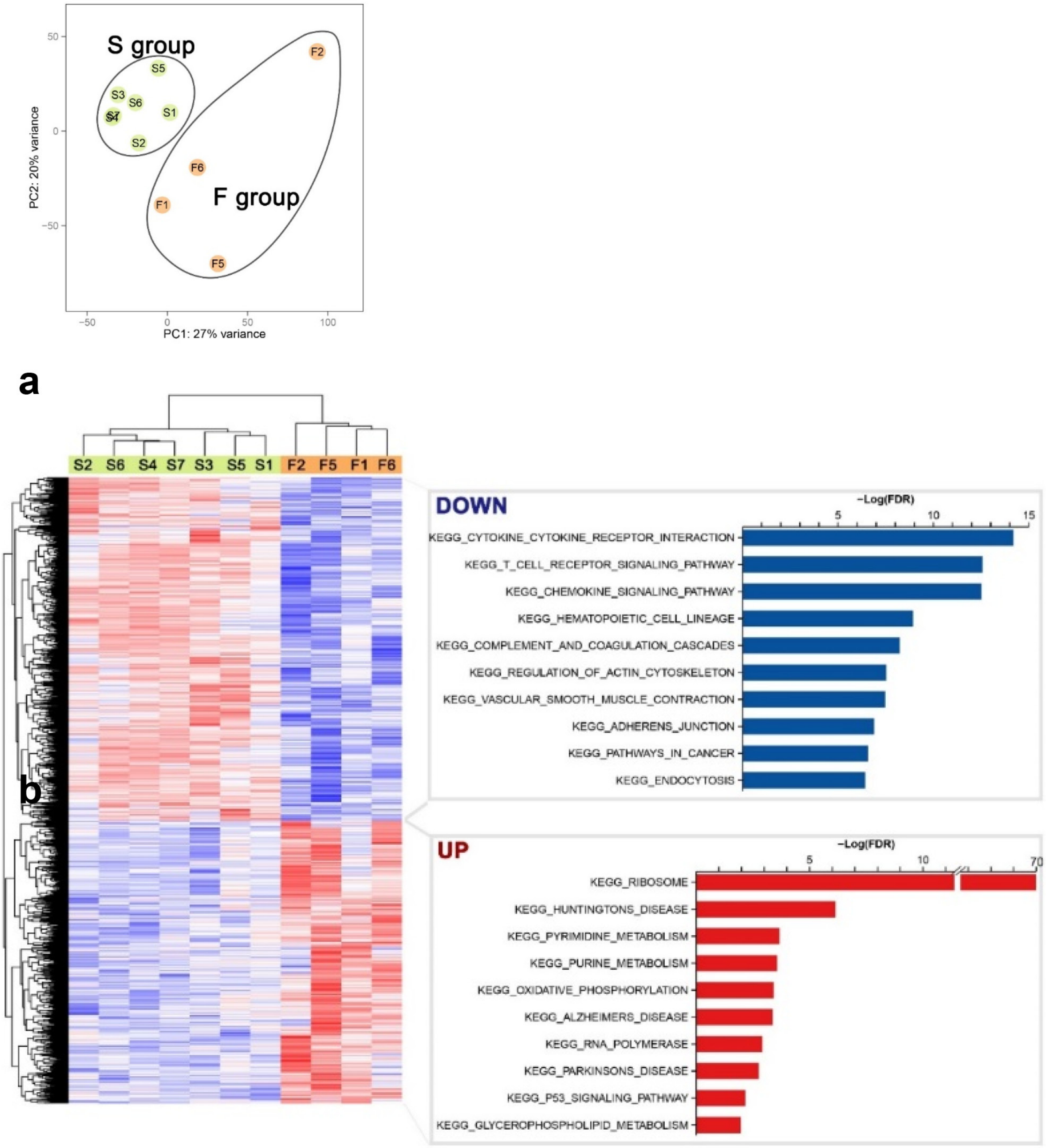

Supplementary Figure S6: Expression profiles. a. Principal component analysis (PCA) of normalized read count of ten RNA-seq samples. Green and orange dots represent responsible and refractory cases, respectively. **b.** Hierarchical clustering and heat map of differentially expressed genes. In right panel, top 10 KEGG pathways gene sets enriched in up- and down-regulate genes are visualized with bar plot, arranged in significance order.

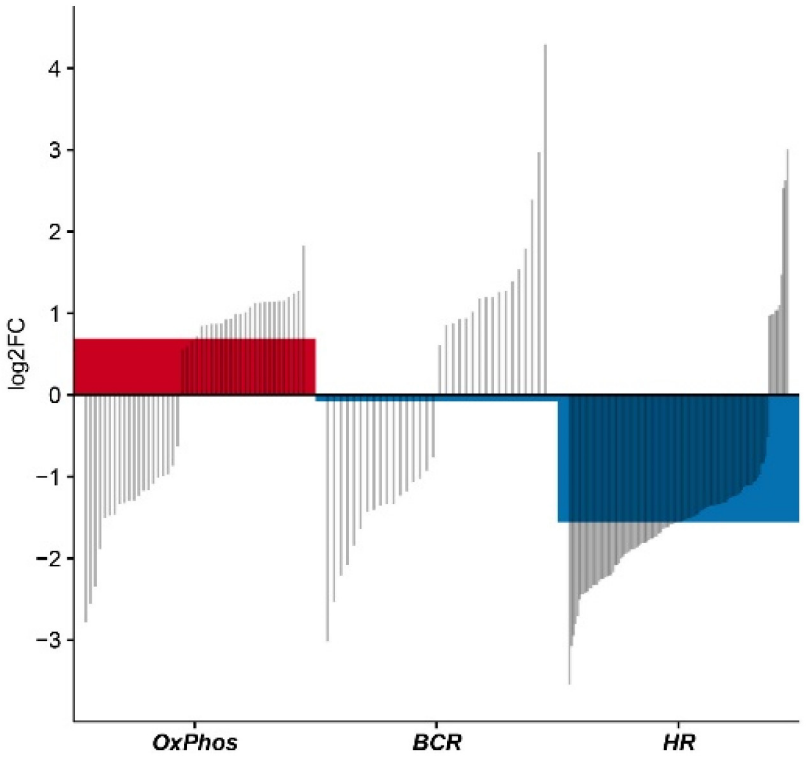

Supplementary Figure S7: Relative expression levels for three different subsets by Margaret A. Shipp et al. (Molecular profiling of diffuse large B-cell lymphoma identifies robust subtypes including one characterized by host inflammatory response. Blood. 2005 Mar 1;105(5):1851-61.) Each thin grey bar represents log2 fold change (FC) of refractory to responsible group of each genes in the subsets. Rectangles at the bottom layer represent median log2FC of each group, positive number in red and negative in blue. OxPhos, oxidative phosphorylation; BCR B-cell receptor/proliferation; HR, host response.

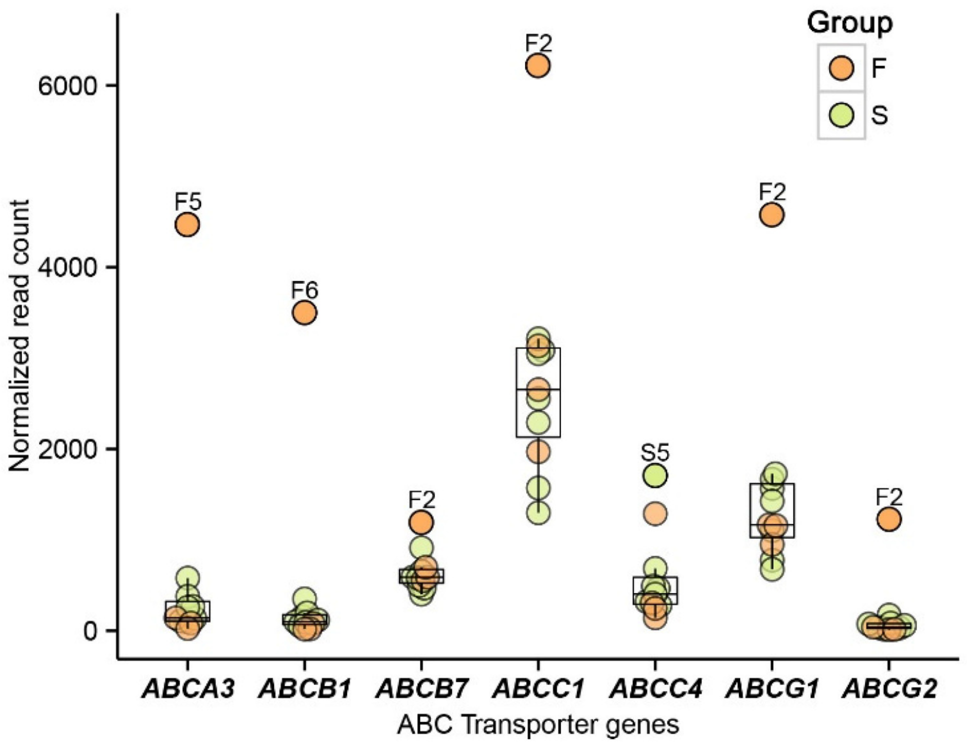

Supplementary Figure S8: Outlier expression of ABC transporter genes. Normalized expression value are plotted in dots with different colors by the group. Expression distribution is represented with boxplot for each ABC transporter genes.

Supplementary Table S1: Clinical information and sequencing platforms

Group	Sample ID	Age	Gender	Sample site	Primary therapy outcome	Days to recur	cd10	bcl-6	mum-1	Han's classification	Exome	RNA-Seq
Responsible	S1	42	F	Lymph node (neck)	CR		−	+	−	GCB	T-N pair	T only
	S2	71	M	Lymph node (supraclavicular)	CR		−	+	+	non-GCB	T-N pair	T only
	S3	66	M	Lymph node (mesenteray)	CR		−	+	+	non-GCB	T-N pair	T only
	S4	63	M	Lymph node (neck)	CR		−	+	+	non-GCB	T-N pair	T only
	S5	53	M	Lymph node (inguinal)	CR		+	+	−	GCB	T only	T only
	S6	39	M	Lymph node (neck)	CR		−	+	−	GCB	Not done	T only
	S7	58	M	Lymph node (neck)	CR		+	+	−	GCB	Not done	T only
Refractory	F1	54	F	Lymph node (supraclavicular)	CR	6 month	−	+	+	non-GCB	T-N pair	T only
	F2	40	M	Lymph node (neck)	CR	4 month	+	+	−	GCB	T-N pair	T only
	F3	69	F	Ileum	PD	death (6 month)	−	+	+	non-GCB	T-N pair	NA
	F4	68	M	Lymph node (mesenteray)	PD	death (2yr 11 month)	+	na	na	GCB	T-N pair	NA
	F5	50	F	Lymph node (neck)	CR	3 month	−	+	−	GCB	T only	T only
	F6	53	M	Lymph node (mediastinum)	CR	4 month	−	+	−	GCB	Not done	T only

Supplementary Table S2: Whole exome sequencing summary

Sample ID	# of total reads	% of reads mapped to reference	% of duplicate reads	% of reads aligned to target regions	Mean read depth on target regions (x)
**Tumor**					
**S1**	90314562	97.64	7.03	40.48	51.31
**S2**	89304243	97.73	6.79	39.17	49.17
**S3**	82705006	97.63	6.85	40.39	46.83
**S4**	88308463	97.38	7.66	41.87	51.06
**S5**	121773677	97.67	7.17	35.12	59.65
**F1**	142828993	97.6	7.02	38.77	77.47
**F2**	101670016	96.44	7.45	40.58	55.63
**F3**	119612876	97.64	6.63	37.27	62.76
**F4**	89073172	97.64	6.59	39.23	49.08
**F5**	111071310	97.87	6.38	36.76	57.71
**Normal**					
**NS1**	161468032	99.7	12.14	81.33	104.22
**NS2**	149314396	99.74	12.48	83.54	98.63
**NS3**	163282595	99.71	13.28	80.57	103.37
**NS4**	158057091	99.73	9.79	82.6	106.42
**NF1**	156085378	99.74	19.47	84.62	96.21
**NF2**	150658597	99.7	11.18	85.24	103.11
**NF3**	159141062	99.71	15.95	84.1	101.76
**NF4**	146550626	99.7	13.64	84.43	96.5

Supplementary Table S3: Transcriptome sequencing summary

Sample ID	# of mapped reads	Intragenic Rate	Exonic Rate	Intronic Rate	Intergenic Rate	Transcripts Detected	Genes Detected
**S1**	31,820,363	0.962	0.835	0.127	0.037	108,563	20,632
**S2**	42,629,246	0.969	0.881	0.088	0.03	110,277	21,051
**S3**	45,898,941	0.969	0.879	0.09	0.03	113,886	21,464
**S4**	48,685,545	0.97	0.89	0.081	0.029	111,206	21,050
**S5**	35,994,192	0.96	0.831	0.129	0.039	112,282	21,368
**S6**	48,627,721	0.967	0.873	0.094	0.032	111,250	21,132
**S7**	51,747,298	0.966	0.859	0.107	0.033	113,292	21,782
**F1**	41,376,928	0.96	0.824	0.136	0.04	107,741	20,881
**F2**	46,926,205	0.961	0.863	0.098	0.038	109,010	21,254
**F5**	25,365,091	0.946	0.808	0.137	0.054	98,802	19,239
**F6**	16,483,718	0.945	0.83	0.115	0.055	96,162	18,447

Supplementary Table S4: List of identified SNVs and InDels

See Supplementary File 1

Supplementary Table S5: Fusion gene candidates

Gene (5')	Gene (3')	Chr (5')	Chr (3')	Breakpoint position (5')	Breakpoint position(3')	Breakpoint location (5')	Breakpoint location (3')	Frame	# of split reads	# of spanning reads	Sample ID
RFC4	ST6GAL1	3	3	186518906	186756530	coding	5' UTR	in	14	6	S1
ST6GAL1	HNRNPC	3	14	186681714	21702388	5' UTR	5' UTR	in	84	24	S1
HNRNPC	BCL6	14	3	21731470	187452695	5' UTR	5' UTR	in	14	7	S1
TOMM40	IGLL5	19	22	45394946	23237555	coding	coding	in	42	17	S4
ATG5	PRDM1	6	6	106740903	106552700	coding	coding	out	14	10	S4
PRDM1	ATG5	6	6	106547427	106727698	coding	coding	out	52	15	S4
REL	BCL11A	2	2	61147777	60679801	coding	coding	in	2036	610	F2
PIM1	MAPK13	6	6	37138654	36098395	coding	coding	in	16	5	F5
MAPK13	LMF1	6	16	36100456	921341	coding	coding	out	42	8	F5

Supplementary Table S6: Overrepresented gene sets of differentially expressed genes

See Supplementary File 1

Supplementary Table S7: Overrepresented gene sets of cancer gene outliers

See Supplementary File 1
