# Supplementary material for: Whole-exome and transcriptome sequencing of refractory diffuse large B-cell lymphoma
Source: Oncotarget. 2016 Nov 9;7(52):86433–45. doi: 10.18632/oncotarget.13239 (PMC5349924; doi:10.18632/oncotarget.13239)
Supplement: Supplementary file 2 [file oncotarget-07-86433-s002.docx]

**Supplementary table 4. List of identified SNVs and InDels**

| **Sample ID** | **Chr** | **Pos** | **Ref** | **Alt** | **Gene Symbol** | **Variant Type** | **Transcript ID** | **Amino acid change** | **# of COSMIC occurrence** | **CADD  score** | **Exome TumorFreq** | **Exome Tumor**  **Ref AC** | **Exome Tumor**  **Alt AC** | **Exome Normal Freq** | **Exome Normal**  **Ref AC** | **Exome Normal Alt AC** | **RNA-Seq Tumor Freq** | **RNA-Seq Tumor Ref AC** | **RNA-Seq Tumor Alt AC** |
| --- | --- | --- | --- | --- | --- | --- | --- | --- | --- | --- | --- | --- | --- | --- | --- | --- | --- | --- | --- |
| F1 | 17 | 7578427 | T | G | TP53 | missense | NM_000546 | p.H168P | 12 | 22.8 | 64% | 4 | 7 | 0% | 85 | 0 | 91% | 11 | 118 |
| F4 | 17 | 7577094 | G | A | TP53 | missense | NM_000546 | p.R282W | 440 | 33 | 80% | 2 | 8 | 0% | 78 | 0 | . | . | . |
| F5 | 17 | 7577505 | T | A | TP53 | missense | NM_000546 | p.D259V | 17 | 24.4 | 100% | 0 | 6 | . | . | . | 97% | 1 | 38 |
| F5 | 17 | 7577590 | T | A | TP53 | missense | NM_000546 | p.T231S | 0 | 28.2 | 83% | 1 | 5 | . | . | . | 100% | 0 | 39 |
| F1 | 3 | 38182641 | T | C | MYD88 | missense | NM_002468 | p.L265P | 1082 | 32 | 59% | 30 | 44 | 0% | 105 | 0 | 46% | 89 | 77 |
| S7 | 3 | 38182641 | T | C | MYD88 | missense | NM_002468 | p.L265P | 1082 | 32 | . | . | . | . | . | . | 10% | 231 | 27 |
| F5 | 3 | 38182337 | C | T | MYD88 | missense | NM_002468 | p.P258L | 0 | 34 | 42% | 21 | 15 | . | . | . | 52% | 15 | 16 |
| F5 | 3 | 38182638 | G | A | MYD88 | missense | NM_002468 | p.R264Q | 0 | 35 | 33% | 24 | 12 | . | . | . | 62% | 13 | 21 |
| S1 | 12 | 7061202 | T | A | PTPN6 | missense | NM_002831 | p.L63Q | 0 | 29.8 | 69% | 5 | 11 | 0% | 152 | 0 | 72% | 100 | 261 |
| F3 | 12 | 7060840 | G | A | PTPN6 | missense | NM_002831 | p.S26N | 0 | 24.3 | 40% | 9 | 6 | 0% | 118 | 0 | . | . | . |
| F3 | 12 | 7061217 | A | C | PTPN6 | missense | NM_002831 | p.K68T | 0 | 25.1 | 58% | 13 | 18 | 0% | 110 | 0 | . | . | . |
| S6 | 12 | 7061328 | C | T | PTPN6 | missense | NM_002831 | p.P105L | 0 | 34 | . | . | . | . | . | . | 17% | 527 | 110 |
| S2 | 6 | 37139063 | G | A | PIM1 | missense | NM_001243186 | p.E226K | 2 | 24.5 | 27% | 22 | 8 | 0% | 86 | 0 | 37% | 306 | 183 |
| S3 | 6 | 37139063 | G | A | PIM1 | missense | NM_001243186 | p.E226K | 2 | 24.5 | 34% | 19 | 10 | 0% | 86 | 0 | 55% | 97 | 120 |
| F1 | 6 | 37138374 | C | T | PIM1 | missense | NM_001243186 | p.S99L | 0 | 24.2 | 38% | 5 | 3 | 0% | 203 | 0 | 27% | 348 | 129 |
| S1 | 15 | 45007789 | A | T | B2M | missense | NM_004048 | p.D79V | 0 | 25.3 | 25% | 50 | 17 | 1% | 188 | 1 | 10% | 857 | 95 |
| F4 | 15 | 45003779 | T | G | B2M | missense | NM_004048 | p.L12R | 0 | 22.8 | 67% | 3 | 6 | 0% | 22 | 0 | . | . | . |
| F6 | 15 | 45008536 | TGTAAG | T | B2M | FS Del | NM_004048 | p.M119fs | 0 | 20.5 | . | . | . | . | . | . | 14% | 349 | 58 |
| S1 | 2 | 230656691 | G | A | TRIP12 | nonsense | NM_001284216 | p.Q1091X | 0 | 41 | 25% | 66 | 22 | 0% | 200 | 0 | 12% | 44 | 6 |
| S4 | 2 | 230654350 | G | A | TRIP12 | nonsense | NM_001284216 | p.R1213X | 1 | 41 | 21% | 44 | 12 | 0% | 41 | 0 | 5% | 196 | 11 |
| F3 | 2 | 230661314 | G | A | TRIP12 | missense | NM_001284216 | p.S925F | 0 | 24.4 | 45% | 29 | 24 | 1% | 66 | 1 | . | . | . |
| S2 | 10 | 107016613 | C | A | SORCS3 | nonsense | NM_014978 | p.S1125X | 0 | 50 | 19% | 25 | 6 | 0% | 18 | 0 | . | 0 | 0 |
| F1 | 10 | 107016592 | C | G | SORCS3 | missense | NM_014978 | p.S1118C | 0 | 23.6 | 30% | 33 | 14 | 0% | 16 | 0 | . | 0 | 0 |
| F5 | 10 | 106865247 | G | T | SORCS3 | missense | NM_014978 | p.V396F | 0 | 31 | 55% | 24 | 29 | . | . | . | . | 0 | 0 |
| S2 | 4 | 85657414 | C | T | WDFY3 | missense | NM_014991 | p.R2275H | 1 | 35 | 31% | 29 | 13 | 0% | 157 | 0 | 0% | 6 | 0 |
| F2 | 4 | 85722830 | G | A | WDFY3 | missense | NM_014991 | p.S932F | 0 | 31 | 44% | 59 | 47 | 0% | 24 | 0 | . | 0 | 0 |
| F5 | 4 | 85625552 | A | G | WDFY3 | missense | NM_014991 | p.M2794T | 0 | 27.9 | 46% | 43 | 36 | . | . | . | . | 0 | 0 |
| F4 | 2 | 73303234 | T | C | RAB11FIP5 | missense | NM_015470 | p.S549G | 0 | 23.7 | 40% | 27 | 18 | 0% | 22 | 0 | . | . | . |
| F4 | 2 | 73316323 | G | C | RAB11FIP5 | missense | NM_015470 | p.D184E | 0 | 25.7 | 14% | 73 | 12 | 0% | 122 | 0 | . | . | . |
| F5 | 2 | 73315364 | C | T | RAB11FIP5 | missense | NM_015470 | p.R461Q | 0 | 23.7 | 45% | 30 | 25 | . | . | . | . | 0 | 0 |
| F5 | X | 12994907 | G | C | TMSB4X | missense | NM_021109 | p.E38Q | 0 | 26.2 | 50% | 75 | 76 | . | . | . | 79% | 200 | 760 |
| S3 | X | 12994904 | CAGG | C | TMSB4X | nonFS Del | NM_021109 | p.37_38del | 0 | 22.8 | 56% | 37 | 47 | 0% | 14 | 0 | 36% | 4359 | 2495 |
| F3 | 11 | 47767924 | G | A | FNBP4 | missense | NM_015308 | p.A310V | 0 | 29.9 | 39% | 46 | 30 | 0% | 75 | 0 | . | . | . |
| F6 | 11 | 47744542 | C | CT | FNBP4 | FS Ins | NM_015308 | p.G931fs | 0 | 35 | . | . | . | . | . | . | 35% | 15 | 8 |
| S2 | 1 | 39924917 | A | G | MACF1 | missense | NM_012090 | p.E5060G | 0 | 28.5 | 12% | 29 | 4 | 0% | 80 | 0 | 1% | 78 | 1 |
| S7 | 1 | 39901431 | C | G | MACF1 | missense | NM_012090 | p.Q3934E | 0 | 27.2 | . | . | . | . | . | . | 51% | 18 | 19 |
| F3 | 16 | 23847583 | G | C | PRKCB | missense | NM_002738 | p.K29N | 0 | 24.3 | 16% | 16 | 3 | 0% | 14 | 0 | . | . | . |
| F5 | 16 | 24135291 | A | G | PRKCB | missense | NM_002738 | p.S352G | 0 | 31 | 35% | 34 | 18 | . | . | . | 33% | 4 | 2 |
| S3 | 12 | 49437710 | G | A | KMT2D | nonsense | NM_003482 | p.Q1754X | 0 | 37 | 30% | 50 | 21 | 0% | 98 | 0 | 23% | 10 | 3 |
| S3 | 12 | 49437514 | C | CA | KMT2D | FS Ins | NM_003482 | p.A1791fs | 0 | 34 | 32% | 38 | 18 | 0% | 129 | 0 | 50% | 7 | 7 |
| F5 | 12 | 49425612 | TGGGGCAGGGAGCCGGGGTGGGCCCTGAGGTCGAGGCCCTGCCCCTAGCTCCTGGA | T | KMT2D | FS Del | NM_003482 | p.L4274fs | 0 | 35 | 38% | 10 | 6 | . | . | . | 0% | 5 | 0 |
| S2 | 1 | 16199531 | A | T | SPEN | nonsense | NM_015001 | p.R102X | 0 | 36 | 20% | 35 | 9 | 0% | 138 | 0 | 27% | 16 | 6 |
| S7 | 1 | 16255086 | G | A | SPEN | missense | NM_015001 | p.R784H | 1 | 25.1 | . | . | . | . | . | . | 55% | 23 | 28 |
| S7 | 1 | 16260485 | G | GA | SPEN | FS Ins | NM_015001 | p.E2584fs | 0 | 29.9 | . | . | . | . | . | . | 14% | 99 | 16 |
| F4 | 7 | 17379347 | A | C | AHR | missense | NM_001621 | p.E633A | 0 | 23.9 | 51% | 35 | 36 | 0% | 182 | 0 | . | . | . |
| F4 | 7 | 17379377 | A | C | AHR | missense | NM_001621 | p.K643T | 0 | 24.1 | 47% | 41 | 37 | 0% | 207 | 0 | . | . | . |
| F5 | 7 | 17379260 | C | A | AHR | missense | NM_001621 | p.S604Y | 0 | 23.6 | 22% | 69 | 19 | . | . | . | 13% | 13 | 2 |
| F4 | 17 | 38557196 | C | G | TOP2A | missense | NM_001067 | p.G857A | 0 | 26.2 | 11% | 195 | 23 | 0% | 51 | 0 | . | . | . |
| F4 | 17 | 38557235 | A | C | TOP2A | missense | NM_001067 | p.I844S | 0 | 28.5 | 10% | 170 | 19 | 0% | 46 | 0 | . | . | . |
| S6 | 17 | 38561120 | G | GT | TOP2A | FS Ins | NM_001067 | p.Q657fs | 0 | 35 | . | . | . | . | . | . | 14% | 340 | 54 |
| S1 | 6 | 138201320 | C | G | TNFAIP3 | nonsense | NM_001270507 | p.Y673X | 0 | 47 | 35% | 26 | 14 | 0% | 113 | 0 | 26% | 97 | 34 |
| S2 | 6 | 138199968 | GACAGCAC | G | TNFAIP3 | FS Del | NM_001270507 | p.T463fs | 0 | 35 | 33% | 12 | 6 | 0% | 70 | 0 | 45% | 186 | 153 |
| S1 | 19 | 6586182 | C | A | CD70 | missense | NM_001252 | p.R144L | 0 | 26.7 | 60% | 4 | 6 | 0% | 59 | 0 | 98% | 11 | 446 |
| F3 | 19 | 6590875 | G | A | CD70 | nonsense | NM_001252 | p.Q47X | 0 | 32 | 36% | 18 | 10 | 0% | 220 | 0 | . | . | . |
| S1 | 6 | 44216451 | A | C | HSP90AB1 | missense | NM_001271969 | p.I29L | 0 | 28.9 | 33% | 99 | 49 | 0% | 41 | 0 | 38% | 289 | 176 |
| F4 | 6 | 44216446 | T | G | HSP90AB1 | missense | NM_001271969 | p.L27R | 0 | 29 | 38% | 100 | 60 | 0% | 48 | 0 | . | . | . |
| S1 | 19 | 42600030 | T | A | POU2F2 | missense | NM_001207025 | p.T239S | 4 | 25.1 | 28% | 23 | 9 | 0% | 104 | 0 | 28% | 159 | 62 |
| S2 | 19 | 42599990 | T | G | POU2F2 | missense | NM_001207025 | p.K252T | 0 | 26.4 | 18% | 18 | 4 | 0% | 101 | 0 | 56% | 207 | 263 |
| S3 | 17 | 62006798 | T | C | CD79B | missense | NM_021602 | p.Y92C | 13 | 24.2 | 26% | 17 | 6 | 0% | 80 | 0 | 48% | 390 | 365 |
| S7 | 17 | 62006799 | A | T | CD79B | missense | NM_021602 | p.Y92N | 7 | 25.1 | . | . | . | . | . | . | 31% | 316 | 144 |
| F4 | 15 | 91352390 | G | A | BLM | missense | NM_001287247 | p.V1128I | 0 | 22.7 | 51% | 41 | 42 | 0% | 72 | 0 | . | . | . |
| S7 | 15 | 91293074 | C | CT | BLM | FS Ins | NM_000057 | p.N192fs | 0 | 33 | . | . | . | . | . | . | 20% | 33 | 8 |
| F3 | 6 | 31324484 | G | T | HLA-B | nonsense | NM_005514 | p.Y108X | 0 | 37 | 28% | 13 | 5 | 0% | 68 | 0 | . | . | . |
| S6 | 6 | 31324489 | C | CGCGGT | HLA-B | FS Ins | NM_005514 | p.G107fs | 0 | 23.2 | . | . | . | . | . | . | 25% | 36 | 12 |
| S3 | 19 | 17440576 | C | T | ANO8 | missense | NM_020959 | p.R465H | 0 | 34 | 50% | 3 | 3 | 0% | 29 | 0 | 0% | 6 | 0 |
| F4 | 19 | 17439046 | C | T | ANO8 | nonsense | NM_020959 | p.W717X | 0 | 41 | 60% | 2 | 3 | 0% | 212 | 0 | . | . | . |
| F1 | 14 | 81251859 | G | A | CEP128 | nonsense | NM_152446 | p.Q531X | 0 | 37 | 23% | 40 | 12 | 0% | 76 | 0 | 0% | 3 | 0 |
| F4 | 14 | 81329150 | A | T | CEP128 | missense | NM_152446 | p.V238E | 0 | 27 | 49% | 24 | 23 | 0% | 243 | 0 | . | . | . |
| S2 | 19 | 16437726 | G | A | KLF2 | missense | NM_016270 | p.E318K | 1 | 24.9 | 36% | 9 | 5 | 0% | 93 | 0 | 13% | 125 | 19 |
| F3 | 19 | 16437750 | C | T | KLF2 | missense | NM_016270 | p.H326Y | 0 | 23.2 | 40% | 6 | 4 | 0% | 75 | 0 | . | . | . |
| S3 | 19 | 19260039 | A | G | MEF2B | missense | NM_001145785 | p.L85P | 0 | 26.2 | 83% | 1 | 5 | 0% | 43 | 0 | 65% | 39 | 74 |
| F3 | 19 | 19260088 | A | C | MEF2B | missense | NM_001145785 | p.Y69D | 0 | 26.9 | 50% | 9 | 9 | 0% | 37 | 0 | . | . | . |
| F1 | 21 | 43256236 | G | A | PRDM15 | missense | NM_001040424 | p.H459Y | 0 | 25.8 | 14% | 19 | 3 | 0% | 16 | 0 | 59% | 20 | 29 |
| F2 | 21 | 43248635 | T | G | PRDM15 | missense | NM_001040424 | p.N511T | 0 | 25.8 | 48% | 60 | 56 | 0% | 88 | 0 | 100% | 0 | 2 |
| S1 | 12 | 79693297 | A | G | SYT1 | missense | NM_001135806 | p.E259G | 0 | 25.5 | 30% | 46 | 20 | 0% | 80 | 0 | . | 0 | 0 |
| F2 | 12 | 79747310 | C | T | SYT1 | missense | NM_001135806 | p.S280F | 0 | 34 | 34% | 75 | 38 | 0% | 92 | 0 | . | 0 | 0 |
| F2 | 2 | 197990182 | A | C | ANKRD44 | nonsense | NM_001195144 | p.L158X | 0 | 43 | 41% | 32 | 22 | 0% | 33 | 0 | 8% | 23 | 2 |
| S6 | 2 | 197872537 | T | A | ANKRD44 | missense | NM_001195144 | p.H708L | 0 | 31 | . | . | . | . | . | . | 44% | 43 | 34 |
| F4 | 2 | 28117427 | T | C | BRE | missense | NM_001261840 | p.S2P | 0 | 27.5 | 33% | 52 | 26 | 0% | 212 | 0 | . | . | . |
| F6 | 2 | 28521300 | C | CA | BRE | FS Ins | NM_001261840 | p.Q344fs | 0 | 35 | . | . | . | . | . | . | 21% | 41 | 11 |
| F3 | 1 | 203276424 | G | T | BTG2 | missense | NM_006763 | p.R112L | 0 | 28.2 | 83% | 4 | 19 | 0% | 73 | 0 | . | . | . |
| S6 | 1 | 203274836 | G | T | BTG2 | missense | NM_006763 | p.R34S | 1 | 21.8 | . | . | . | . | . | . | 14% | 476 | 77 |
| S4 | 20 | 55027914 | G | T | CASS4 | missense | NM_001164114 | p.R507M | 0 | 32 | 19% | 34 | 8 | 0% | 97 | 0 | 0% | 18 | 0 |
| S6 | 20 | 55027224 | T | A | CASS4 | missense | NM_001164114 | p.V277D | 0 | 31 | . | . | . | . | . | . | 53% | 9 | 10 |
| S3 | 2 | 125555733 | C | A | CNTNAP5 | missense | NM_130773 | p.P1017H | 0 | 26.1 | 10% | 94 | 11 | 0% | 368 | 1 | . | 0 | 0 |
| F5 | 2 | 125204474 | G | A | CNTNAP5 | missense | NM_130773 | p.R293H | 1 | 24.4 | 27% | 30 | 11 | . | . | . | . | 0 | 0 |
| F3 | 4 | 105412064 | T | C | CXXC4 | missense | NM_025212 | p.N299S | 0 | 22 | 19% | 46 | 11 | 0% | 57 | 0 | . | . | . |
| F5 | 4 | 105393505 | C | T | CXXC4 | missense | NM_025212 | p.A360T | 0 | 24.6 | 42% | 94 | 68 | . | . | . | . | 0 | 0 |
| F4 | 6 | 30630729 | C | A | DHX16 | missense | NM_001164239 | p.A403S | 0 | 24 | 50% | 13 | 13 | 0% | 90 | 0 | . | . | . |
| F6 | 6 | 30621047 | A | AT | DHX16 | FS Ins | NM_001164239 | p.I973fs | 1 | 35 | . | . | . | . | . | . | 18% | 59 | 13 |
| S1 | 17 | 7736768 | G | A | DNAH2 | missense | NM_020877 | p.G4401S | 0 | 25 | 29% | 17 | 7 | 0% | 214 | 0 | 0% | 1 | 0 |
| F5 | 17 | 7734160 | G | T | DNAH2 | missense | NM_020877 | p.R4077L | 0 | 26.8 | 94% | 1 | 15 | . | . | . | . | 0 | 0 |
| S1 | 10 | 120825015 | G | A | EIF3A | nonsense | NM_003750 | p.R340X | 0 | 42 | 30% | 30 | 13 | 0% | 102 | 0 | 12% | 57 | 8 |
| S6 | 10 | 120833028 | G | GT | EIF3A | FS Ins | NM_003750 | p.T101fs | 0 | 35 | . | . | . | . | . | . | 14% | 180 | 30 |
| F2 | 2 | 55144001 | A | C | EML6 | missense | NM_001039753 | p.T1202P | 0 | 24.5 | 28% | 160 | 62 | 0% | 51 | 0 | 43% | 17 | 13 |
| S5 | 2 | 54952332 | T | G | EML6 | missense | NM_001039753 | p.V45G | 0 | 24.2 | 56% | 4 | 5 | . | . | . | 9% | 21 | 2 |
| S6 | 1 | 234745130 | G | C | IRF2BP2 | missense | NM_001077397 | p.C37W | 0 | 23.5 | . | . | . | . | . | . | 32% | 58 | 27 |
| S2 | 1 | 234744274 | T | TCG | IRF2BP2 | FS Ins | NM_001077397 | p.S323fs | 0 | 32 | 50% | 4 | 4 | 0% | 121 | 0 | 24% | 175 | 56 |
| F2 | 4 | 123132210 | C | G | KIAA1109 | missense | NM_015312 | p.T736S | 0 | 23.2 | 41% | 35 | 24 | 0% | 313 | 0 | 67% | 1 | 2 |
| S7 | 4 | 123254828 | C | CA | KIAA1109 | FS Ins | NM_015312 | p.S3837fs | 0 | 36 | . | . | . | . | . | . | 22% | 35 | 10 |
| F3 | 12 | 27950854 | A | C | KLHL42 | missense | NM_020782 | p.T425P | 0 | 25.7 | 13% | 26 | 4 | 1% | 75 | 1 | . | . | . |
| S5 | 12 | 27950854 | A | C | KLHL42 | missense | NM_020782 | p.T425P | 0 | 25.7 | 26% | 17 | 6 | . | . | . | 0% | 17 | 0 |
| S2 | 1 | 225609894 | G | A | LBR | missense | NM_002296 | p.S84F | 0 | 27.3 | 11% | 31 | 4 | 0% | 70 | 0 | 23% | 130 | 38 |
| F6 | 1 | 225592201 | C | T | LBR | missense | NM_002296 | p.G531E | 0 | 34 | . | . | . | . | . | . | 27% | 8 | 3 |
| F1 | 6 | 76623836 | C | T | MYO6 | nonsense | NM_001300899 | p.R1143X | 0 | 47 | 41% | 119 | 83 | 0% | 62 | 0 | 0% | 12 | 0 |
| F5 | 6 | 76618317 | C | T | MYO6 | missense | NM_001300899 | p.R1106C | 0 | 35 | 29% | 84 | 34 | . | . | . | 100% | 0 | 2 |
| F3 | 17 | 43138797 | G | A | NMT1 | missense | NM_021079 | p.E34K | 0 | 26 | 33% | 6 | 3 | 0% | 10 | 0 | . | . | . |
| F6 | 17 | 43159061 | C | CA | NMT1 | FS Ins | NM_021079 | p.Q61fs | 0 | 35 | . | . | . | . | . | . | 40% | 12 | 8 |
| F4 | 5 | 142680092 | G | A | NR3C1 | missense | NM_000176 | p.R569W | 1 | 34 | 48% | 89 | 83 | 0% | 112 | 0 | . | . | . |
| S7 | 5 | 142680307 | A | AT | NR3C1 | FS Ins | NM_000176 | p.I497fs | 0 | 35 | . | . | . | . | . | . | 18% | 84 | 19 |
| S1 | 5 | 140187158 | A | C | PCDHA4 | missense | NM_018907 | p.N129T | 0 | 23.4 | 14% | 51 | 8 | 0% | 27 | 0 | . | 0 | 0 |
| F5 | 5 | 140187158 | A | C | PCDHA4 | missense | NM_018907 | p.N129T | 0 | 23.4 | 12% | 76 | 10 | . | . | . | . | 0 | 0 |
| S1 | 5 | 140207785 | G | A | PCDHA6 | missense | NM_018909 | p.E37K | 0 | 26.5 | 26% | 45 | 16 | 0% | 68 | 0 | . | 0 | 0 |
| F5 | 5 | 140209172 | T | G | PCDHA6 | missense | NM_018909 | p.V499G | 0 | 23.8 | 23% | 20 | 6 | . | . | . | . | 0 | 0 |
| F2 | 1 | 171556149 | T | A | PRRC2C | missense | NM_015172 | p.V2584E | 0 | 27.1 | 31% | 72 | 33 | 1% | 196 | 1 | 7% | 65 | 5 |
| S7 | 1 | 171519316 | C | CA | PRRC2C | FS Ins | NM_015172 | p.S1686fs | 0 | 35 | . | . | . | . | . | . | 16% | 141 | 26 |
| F1 | 6 | 111726774 | G | A | REV3L | missense | NM_002912 | p.A155V | 0 | 29.4 | 48% | 65 | 61 | 0% | 26 | 0 | 100% | 0 | 2 |
| S6 | 6 | 111701224 | C | T | REV3L | missense | NM_002912 | p.R472K | 0 | 28.9 | . | . | . | . | . | . | 65% | 6 | 11 |
| S2 | 13 | 26793784 | C | T | RNF6 | missense | NM_005977 | p.M1I | 0 | 23.2 | 29% | 54 | 22 | 0% | 39 | 0 | 40% | 61 | 40 |
| S7 | 13 | 26788240 | T | TA | RNF6 | FS Ins | NM_005977 | p.L593fs | 1 | 35 | . | . | . | . | . | . | 18% | 115 | 25 |
| S1 | 16 | 89940082 | C | CG | TCF25 | FS Ins | NM_014972 | p.R3fs | 0 | 34 | 43% | 4 | 3 | 0% | 48 | 0 | 0% | 41 | 0 |
| F6 | 16 | 89951019 | G | GA | TCF25 | FS Ins | NM_014972 | p.K128fs | 0 | 34 | . | . | . | . | . | . | 41% | 10 | 7 |
| F2 | 9 | 100193273 | G | C | TDRD7 | missense | NM_001302884 | p.R15P | 1 | 32 | 10% | 62 | 7 | 0% | 56 | 0 | 0% | 4 | 0 |
| S7 | 9 | 100194362 | C | CA | TDRD7 | FS Ins | NM_001302884 | p.G61fs | 0 | 26.9 | . | . | . | . | . | . | 20% | 43 | 11 |
| F2 | 19 | 58083884 | T | C | ZNF416 | missense | NM_017879 | p.E463G | 0 | 21.8 | 47% | 36 | 32 | 0% | 139 | 0 | 59% | 9 | 13 |
| S7 | 19 | 58083764 | A | T | ZNF416 | missense | NM_017879 | p.F503Y | 0 | 27.1 | . | . | . | . | . | . | 40% | 6 | 4 |
| S1 | 18 | 6992691 | C | A | LAMA1 | missense | NM_005559 | p.Q1679H | 0 | 24.7 | 25% | 39 | 13 | 0% | 70 | 0 | . | 0 | 0 |
| F4 | 2 | 196651777 | C | A | DNAH7 | missense | NM_018897 | p.R3612I | 0 | 33 | 16% | 46 | 9 | 0% | 47 | 0 | . | . | . |
| F4 | 2 | 196651778 | T | A | DNAH7 | nonsense | NM_018897 | p.R3612X | 0 | 42 | 16% | 47 | 9 | 0% | 47 | 0 | . | . | . |
| F4 | 11 | 70011639 | C | G | ANO1 | missense | NM_018043 | p.Q672E | 0 | 27.3 | 38% | 5 | 3 | 0% | 28 | 0 | . | . | . |
| S1 | 22 | 50905804 | AG | A | SBF1 | FS Del | NM_002972 | p.L171fs | 0 | 28.6 | 38% | 13 | 8 | 0% | 38 | 0 | 42% | 21 | 15 |
| F3 | 2 | 141128819 | C | A | LRP1B | missense | NM_018557 | p.A3602S | 0 | 23.6 | 22% | 38 | 11 | 0% | 158 | 0 | . | . | . |
| F3 | 20 | 37383735 | G | A | ACTR5 | missense | NM_024855 | p.R304Q | 1 | 31 | 60% | 8 | 12 | 0% | 19 | 0 | . | . | . |
| F3 | 17 | 71231731 | C | T | C17orf80 | missense | NM_001100621 | p.T37I | 0 | 22.9 | 50% | 21 | 21 | 0% | 387 | 0 | . | . | . |
| S2 | 5 | 127668651 | T | A | FBN2 | missense | NM_001999 | p.K1392M | 0 | 27.3 | 35% | 53 | 28 | 0% | 140 | 0 | . | 0 | 0 |
| F4 | 11 | 11362523 | A | C | GALNT18 | missense | NM_198516 | p.V374G | 0 | 28.3 | 19% | 35 | 8 | 0% | 31 | 0 | . | . | . |
| F2 | 2 | 24493589 | T | G | ITSN2 | missense | NM_019595 | p.E742A | 0 | 22.8 | 46% | 43 | 36 | 0% | 128 | 0 | 32% | 13 | 6 |
| F3 | 11 | 65309130 | G | T | LTBP3 | missense | NM_001130144 | p.S889Y | 0 | 25.9 | 20% | 20 | 5 | 0% | 65 | 0 | . | . | . |
| F2 | 13 | 109707454 | A | G | MYO16 | missense | NM_001198950 | p.T1037A | 0 | 24.5 | 84% | 3 | 16 | 0% | 122 | 0 | . | 0 | 0 |
| S2 | 12 | 124821347 | T | G | NCOR2 | missense | NM_001077261 | p.T2013P | 0 | 23.4 | 44% | 14 | 11 | 0% | 40 | 0 | 16% | 48 | 9 |
| F4 | 2 | 152522901 | C | A | NEB | missense | NM_001164507 | p.E1578D | 0 | 23.9 | 40% | 47 | 31 | 0% | 154 | 0 | . | . | . |
| S1 | 10 | 46111990 | C | T | ZFAND4 | missense | NM_001128324 | p.R693H | 0 | 34 | 30% | 40 | 17 | 0% | 178 | 0 | 36% | 9 | 5 |
| F4 | 19 | 12060592 | A | G | ZNF700 | missense | NM_001271848 | p.T588A | 0 | 22.9 | 42% | 123 | 88 | 0% | 240 | 0 | . | . | . |
| F2 | 7 | 36194017 | T | A | EEPD1 | nonsense | NM_030636 | p.C28X | 0 | 36 | 70% | 6 | 14 | 0% | 141 | 0 | 56% | 20 | 25 |
| F2 | 7 | 36194181 | T | C | EEPD1 | missense | NM_030636 | p.V83A | 0 | 24.8 | 50% | 8 | 8 | 0% | 55 | 0 | 54% | 17 | 20 |
| F2 | 7 | 36336609 | G | C | EEPD1 | missense | NM_030636 | p.K441N | 0 | 23.8 | 42% | 18 | 13 | 0% | 129 | 0 | 43% | 66 | 50 |
| F2 | 14 | 103363630 | A | C | TRAF3 | missense | NM_001199427 | p.K201N | 0 | 23.1 | 53% | 23 | 26 | 0% | 35 | 0 | 20% | 24 | 6 |
| F2 | 14 | 103363634 | A | T | TRAF3 | nonsense | NM_001199427 | p.K203X | 0 | 43 | 51% | 23 | 24 | 0% | 35 | 0 | 21% | 23 | 6 |
| F2 | 14 | 103363667 | A | T | TRAF3 | missense | NM_001199427 | p.S214C | 0 | 21.3 | 53% | 19 | 21 | 0% | 47 | 0 | 16% | 26 | 5 |
| F4 | 17 | 73917325 | C | A | FBF1 | missense | NM_001080542 | p.Q543H | 0 | 24.5 | 17% | 29 | 6 | 0% | 29 | 0 | . | . | . |
| F4 | 17 | 73917327 | G | A | FBF1 | nonsense | NM_001080542 | p.Q543X | 0 | 36 | 17% | 29 | 6 | 0% | 29 | 0 | . | . | . |
| F2 | 4 | 48546850 | A | G | FRYL | missense | NM_015030 | p.L1784S | 0 | 27.9 | 44% | 63 | 50 | 0% | 149 | 0 | 67% | 2 | 4 |
| F2 | 4 | 48559045 | A | C | FRYL | missense | NM_015030 | p.F1406V | 0 | 26.3 | 26% | 14 | 5 | 0% | 62 | 0 | 60% | 2 | 3 |
| F2 | 11 | 18332952 | T | G | HPS5 | missense | NM_181507 | p.Y89S | 0 | 25.2 | 18% | 32 | 7 | 0% | 38 | 0 | 47% | 10 | 9 |
| F2 | 11 | 18332961 | T | A | HPS5 | missense | NM_181507 | p.D86V | 0 | 28.9 | 15% | 34 | 6 | 0% | 36 | 0 | 45% | 11 | 9 |
| F2 | 14 | 69256777 | T | A | ZFP36L1 | missense | NM_001244698 | p.I164F | 0 | 26.8 | 55% | 13 | 16 | 0% | 118 | 0 | 45% | 144 | 116 |
| F2 | 14 | 69259610 | C | T | ZFP36L1 | missense | NM_001244698 | p.V16I | 0 | 22.3 | 58% | 25 | 34 | 0% | 11 | 0 | 58% | 84 | 118 |
| S1 | 3 | 186504961 | A | T | EIF4A2 | missense | NM_001967 | p.I273F | 0 | 26.4 | 38% | 55 | 34 | 0% | 53 | 0 | 29% | 267 | 110 |
| F2 | 17 | 41610083 | A | C | ETV4 | missense | NM_001261437 | p.V218G | 0 | 28.2 | 25% | 21 | 7 | 0% | 9 | 0 | 50% | 1 | 1 |
| S2 | 7 | 2979543 | CGGT | C | CARD11 | nonFS Del | NM_032415 | p.234_235del | 0 | 21.6 | 41% | 24 | 17 | 0% | 169 | 0 | 68% | 39 | 83 |
| F4 | 10 | 89692795 | T | G | PTEN | missense | NM_000314 | p.H93Q | 0 | 24.8 | 91% | 6 | 60 | 0% | 232 | 0 | . | . | . |
| S4 | 4 | 153249385 | G | A | FBXW7 | missense | NM_001013415 | p.R347C | 80 | 35 | 16% | 101 | 19 | 0% | 211 | 0 | 26% | 28 | 10 |
| F3 | X | 70348466 | C | A | MED12 | missense | NM_005120 | p.H1125N | 0 | 32 | 44% | 20 | 16 | 0% | 159 | 0 | . | . | . |
| F2 | 14 | 36989296 | C | G | NKX2-1 | missense | NM_001079668 | p.E13D | 0 | 21.1 | 50% | 4 | 4 | 0% | 86 | 0 | . | 0 | 0 |
| S4 | 9 | 36923456 | G | A | PAX5 | missense | NM_001280551 | p.S161L | 0 | 23.4 | 12% | 22 | 3 | 0% | 153 | 0 | 30% | 168 | 73 |
| S2 | 2 | 198265488 | T | C | SF3B1 | missense | NM_012433 | p.E890G | 0 | 27.5 | 32% | 39 | 18 | 0% | 381 | 0 | 36% | 181 | 101 |
| F3 | 16 | 11348972 | C | T | SOCS1 | missense | NM_003745 | p.G122R | 0 | 26.6 | 43% | 4 | 3 | 0% | 28 | 0 | . | . | . |
| F2 | 4 | 106164728 | T | C | TET2 | missense | NM_001127208 | p.V1199A | 0 | 26.6 | 56% | 4 | 5 | 0% | 11 | 0 | . | 0 | 0 |
| F4 | 16 | 2108786 | T | TG | TSC2 | FS Ins | NM_000548 | p.V296fs | 0 | 35 | 43% | 4 | 3 | 0% | 21 | 0 | . | . | . |
| S1 | 1 | 85733594 | C | A | BCL10 | nonsense | NM_003921 | p.E140X | 1 | 39 | 19% | 65 | 15 | 0% | 26 | 0 | 34% | 47 | 24 |
| F3 | 5 | 149784293 | T | A | CD74 | missense | NM_001025159 | p.E192V | 0 | 33 | 19% | 13 | 3 | 0% | 36 | 0 | . | . | . |
| F1 | 16 | 64981643 | C | G | CDH11 | missense | NM_001797 | p.A752P | 0 | 28.3 | 47% | 46 | 40 | 0% | 219 | 0 | 0% | 13 | 0 |
| F2 | 1 | 51826910 | G | C | EPS15 | missense | NM_001159969 | p.P512R | 0 | 24.1 | 63% | 9 | 15 | 0% | 108 | 0 | 55% | 42 | 52 |
| F4 | 12 | 56492597 | G | C | ERBB3 | missense | NM_001982 | p.R916P | 0 | 24.6 | 39% | 30 | 19 | 1% | 145 | 1 | . | . | . |
| S4 | 12 | 12038902 | C | G | ETV6 | missense | NM_001987 | p.R399G | 0 | 33 | 14% | 72 | 12 | 0% | 72 | 0 | 35% | 81 | 43 |
| S2 | 14 | 38061535 | G | A | FOXA1 | missense | NM_004496 | p.R152C | 0 | 32 | 27% | 8 | 3 | 0% | 75 | 0 | . | 0 | 0 |
| F4 | 16 | 9858094 | G | A | GRIN2A | missense | NM_001134407 | p.R1103C | 0 | 29.3 | 47% | 39 | 34 | 0% | 114 | 0 | . | . | . |
| F2 | 1 | 226924686 | G | C | ITPKB | missense | NM_002221 | p.S158R | 0 | 26.3 | 31% | 31 | 14 | 0% | 117 | 0 | 30% | 69 | 29 |
| S2 | 1 | 164761953 | C | T | PBX1 | missense | NM_001204961 | p.T163M | 0 | 27.4 | 16% | 21 | 4 | 0% | 22 | 0 | 100% | 0 | 1 |
| F1 | 12 | 71029817 | GT | G | PTPRB | FS Del | NM_001109754 | p.K28fs | 0 | 25.9 | 54% | 43 | 50 | 0% | 331 | 0 | . | 0 | 0 |
| S2 | 6 | 128304117 | G | T | PTPRK | nonsense | NM_001291984 | p.Y1131X | 0 | 44 | 23% | 53 | 16 | 0% | 157 | 0 | . | 0 | 0 |
| F2 | X | 153627836 | A | C | RPL10 | missense | NM_001303624 | p.I31L | 0 | 25.7 | 93% | 7 | 94 | 0% | 96 | 0 | 97% | 25 | 917 |
| F2 | 6 | 26156846 | C | G | HIST1H1E | missense | NM_005321 | p.N76K | 1 | 25.8 | 44% | 10 | 8 | 0% | 116 | 0 | 60% | 2 | 3 |
| F4 | 4 | 114209583 | A | G | ANK2 | missense | NM_001148 | p.N740D | 0 | 27.8 | 38% | 55 | 34 | 0% | 105 | 0 | . | . | . |
| S1 | 5 | 41000450 | A | C | MROH2B | missense | NM_173489 | p.C1452G | 0 | 26 | 28% | 75 | 29 | 0% | 83 | 0 | . | 0 | 0 |
| S1 | 7 | 39503846 | G | T | POU6F2 | missense | NM_007252 | p.S546I | 0 | 23.3 | 27% | 120 | 44 | 0% | 270 | 0 | . | 0 | 0 |
| F2 | 19 | 52519476 | C | A | ZNF614 | nonsense | NM_025040 | p.E459X | 0 | 37 | 45% | 59 | 49 | 0% | 198 | 0 | 73% | 3 | 8 |
| S2 | 1 | 155322556 | C | G | ASH1L | missense | NM_018489 | p.R2436P | 0 | 35 | 12% | 43 | 6 | 0% | 40 | 0 | 8% | 12 | 1 |
| F1 | 1 | 203014625 | G | T | PPFIA4 | missense | NM_001304331 | p.A266S | 0 | 24.1 | 50% | 5 | 5 | 0% | 18 | 0 | . | 0 | 0 |
| F3 | 4 | 69682153 | T | G | UGT2B10 | missense | NM_001075 | p.L139R | 0 | 23.3 | 42% | 149 | 106 | 0% | 74 | 0 | . | . | . |
| F1 | 9 | 79252334 | C | T | PRUNE2 | missense | NM_015225 | p.R2988Q | 0 | 35 | 67% | 63 | 127 | 0% | 136 | 0 | 0% | 2 | 0 |
| F4 | 1 | 94481310 | A | G | ABCA4 | missense | NM_000350 | p.L1766P | 0 | 32 | 21% | 34 | 9 | 0% | 58 | 0 | . | . | . |
| F4 | 16 | 48221337 | A | C | ABCC11 | missense | NM_033151 | p.V903G | 0 | 26.6 | 50% | 6 | 6 | 0% | 33 | 0 | . | . | . |
| S2 | 16 | 48177900 | T | A | ABCC12 | nonsense | NM_033226 | p.K66X | 0 | 26.2 | 15% | 17 | 3 | 0% | 22 | 0 | . | 0 | 0 |
| S1 | X | 108917632 | G | T | ACSL4 | missense | NM_004458 | p.P334Q | 0 | 33 | 23% | 41 | 12 | 0% | 36 | 0 | 0% | 59 | 0 |
| F3 | 15 | 35084757 | G | T | ACTC1 | missense | NM_005159 | p.D156E | 0 | 27.3 | 25% | 43 | 14 | 0% | 97 | 0 | . | . | . |
| F4 | 2 | 74136248 | G | A | ACTG2 | missense | NM_001199893 | p.A102T | 0 | 24.1 | 18% | 51 | 11 | 0% | 252 | 0 | . | . | . |
| S1 | 5 | 156991421 | C | G | ADAM19 | missense | NM_033274 | p.A71P | 0 | 32 | 24% | 31 | 10 | 0% | 108 | 0 | 40% | 18 | 12 |
| S1 | 21 | 28212794 | T | G | ADAMTS1 | missense | NM_006988 | p.Q489P | 0 | 27.4 | 25% | 21 | 7 | 0% | 57 | 0 | 0% | 9 | 0 |
| F2 | 1 | 154560716 | A | T | ADAR | missense | NM_001025107 | p.D673E | 0 | 24.6 | 27% | 61 | 23 | 0% | 25 | 0 | 32% | 117 | 56 |
| F4 | 1 | 167823669 | G | C | ADCY10 | missense | NM_001167749 | p.L591V | 0 | 20.2 | 20% | 53 | 13 | 0% | 124 | 0 | . | . | . |
| F2 | 7 | 31146202 | C | A | ADCYAP1R1 | missense | NM_001199637 | p.S416R | 1 | 27.3 | 33% | 16 | 8 | 0% | 103 | 0 | . | 0 | 0 |
| S1 | 1 | 15909777 | A | T | AGMAT | missense | NM_024758 | p.V129D | 0 | 29.6 | 33% | 8 | 4 | 0% | 68 | 0 | 29% | 5 | 2 |
| F4 | 17 | 55184160 | C | G | AKAP1 | missense | NM_003488 | p.S445R | 0 | 20.4 | 12% | 29 | 4 | 0% | 109 | 0 | . | . | . |
| F2 | 12 | 38714843 | A | G | ALG10B | missense | NM_001013620 | p.Y417C | 0 | 24.3 | 45% | 112 | 90 | 0% | 208 | 0 | 50% | 5 | 5 |
| F2 | 17 | 79846121 | G | A | ALYREF | missense | NM_005782 | p.A259V | 0 | 27.7 | 45% | 6 | 5 | 0% | 206 | 0 | 38% | 218 | 134 |
| F1 | 4 | 125592397 | T | C | ANKRD50 | missense | NM_001167882 | p.T500A | 0 | 23.9 | 27% | 69 | 25 | 0% | 152 | 0 | . | 0 | 0 |
| F3 | 20 | 62331964 | C | T | ARFRP1 | missense | NM_001267546 | p.A123T | 0 | 34 | 43% | 4 | 3 | 0% | 116 | 0 | . | . | . |
| F4 | 10 | 104660445 | G | T | AS3MT | missense | NM_020682 | p.K372N | 0 | 21.8 | 59% | 12 | 17 | 0% | 122 | 0 | . | . | . |
| S1 | 18 | 31323535 | C | A | ASXL3 | nonsense | NM_030632 | p.C1241X | 0 | 36 | 32% | 109 | 51 | 0% | 176 | 0 | . | 0 | 0 |
| F1 | 14 | 96779666 | C | A | ATG2B | missense | NM_018036 | p.R1250I | 0 | 34 | 36% | 47 | 27 | 0% | 28 | 0 | 33% | 2 | 1 |
| F1 | 16 | 28890904 | C | G | ATP2A1 | missense | NM_004320 | p.F73L | 0 | 24.8 | 43% | 8 | 6 | 0% | 17 | 0 | 26% | 29 | 10 |
| F2 | 12 | 124228404 | C | T | ATP6V0A2 | missense | NM_012463 | p.R371C | 0 | 34 | 36% | 37 | 21 | 0% | 45 | 0 | 38% | 30 | 18 |
| F4 | 10 | 28971298 | T | C | BAMBI | missense | NM_012342 | p.Y251H | 0 | 25.8 | 42% | 21 | 15 | 0% | 194 | 0 | . | . | . |
| F1 | 16 | 88052099 | A | G | BANP | missense | NM_001173540 | p.I208V | 0 | 22.8 | 42% | 28 | 20 | 0% | 19 | 0 | 32% | 23 | 11 |
| F4 | 15 | 40397998 | T | A | BMF | missense | NM_001003942 | p.Y97F | 0 | 26.6 | 14% | 42 | 7 | 0% | 21 | 0 | . | . | . |
| S3 | 15 | 59961091 | T | C | BNIP2 | missense | NM_004330 | p.Q419R | 0 | 23.5 | 26% | 65 | 23 | 0% | 69 | 0 | 27% | 93 | 34 |
| F4 | 6 | 26388255 | C | A | BTN2A2 | missense | NM_181531 | p.P37T | 1 | 22.9 | 23% | 23 | 7 | 0% | 48 | 0 | . | . | . |
| F3 | 15 | 40632158 | C | T | C15orf52 | missense | NM_207380 | p.R68Q | 1 | 34 | 59% | 11 | 16 | 0% | 28 | 0 | . | . | . |
| S2 | 5 | 179275005 | T | A | C5orf45 | missense | NM_016175 | p.Q63L | 0 | 25.4 | 26% | 59 | 21 | 0% | 43 | 0 | 30% | 23 | 10 |
| S2 | 9 | 77599870 | A | C | C9orf41 | missense | NM_152420 | p.Y361D | 0 | 29.1 | 17% | 29 | 6 | 0% | 178 | 0 | 35% | 41 | 22 |
| F3 | 9 | 114508581 | G | A | C9orf84 | nonsense | NM_001080551 | p.Q235X | 0 | 26.4 | 25% | 49 | 16 | 0% | 48 | 0 | . | . | . |
| F4 | 8 | 86358502 | G | T | CA3 | missense | NM_005181 | p.E213D | 0 | 24.6 | 37% | 12 | 7 | 0% | 34 | 0 | . | . | . |
| F2 | 19 | 13414691 | G | A | CACNA1A | missense | NM_001127221 | p.T666M | 2 | 29.6 | 53% | 36 | 40 | 0% | 135 | 0 | . | 0 | 0 |
| F2 | 3 | 180372608 | A | C | CCDC39 | nonsense | NM_181426 | p.L291X | 0 | 35 | 50% | 101 | 102 | 0% | 390 | 0 | . | 0 | 0 |
| F4 | 17 | 78022525 | G | A | CCDC40 | missense | NM_001243342 | p.E274K | 0 | 22.1 | 50% | 7 | 7 | 0% | 59 | 0 | . | . | . |
| F4 | 2 | 55591118 | T | G | CCDC88A | missense | NM_001135597 | p.T152P | 0 | 26.3 | 20% | 40 | 10 | 0% | 86 | 0 | . | . | . |
| F1 | 19 | 47773834 | C | T | CCDC9 | missense | NM_015603 | p.P325L | 0 | 33 | 42% | 19 | 14 | 0% | 75 | 0 | 55% | 40 | 48 |
| F2 | 19 | 49841227 | T | A | CD37 | missense | NM_001040031 | p.Y62N | 0 | 24 | 58% | 8 | 11 | 0% | 60 | 0 | 51% | 441 | 451 |
| F2 | 16 | 68713863 | G | C | CDH3 | missense | NM_001793 | p.G285R | 0 | 31 | 62% | 13 | 21 | 0% | 41 | 0 | . | 0 | 0 |
| F3 | 7 | 105645117 | C | T | CDHR3 | nonsense | NM_001301161 | p.Q258X | 0 | 35 | 41% | 86 | 60 | 0% | 38 | 0 | . | . | . |
| F2 | 12 | 96704857 | C | A | CDK17 | missense | NM_001170464 | p.M172I | 0 | 22.7 | 50% | 29 | 29 | 0% | 104 | 0 | 46% | 7 | 6 |
| S3 | 15 | 81212481 | C | T | CEMIP | missense | NM_018689 | p.T615M | 0 | 27 | 26% | 23 | 8 | 0% | 294 | 0 | 0% | 70 | 0 |
| F2 | 5 | 64824783 | C | G | CENPK | missense | NM_001267038 | p.E145Q | 0 | 22.7 | 42% | 62 | 45 | 0% | 113 | 0 | 28% | 26 | 10 |
| S2 | 10 | 134650340 | G | T | CFAP46 | missense | NM_001200049 | p.L2173M | 0 | 25.4 | 34% | 23 | 12 | 0% | 25 | 0 | . | 0 | 0 |
| F2 | 21 | 37785189 | G | T | CHAF1B | missense | NM_005441 | p.D357Y | 0 | 32 | 43% | 13 | 10 | 0% | 87 | 0 | 33% | 16 | 8 |
| S3 | 7 | 29544369 | C | T | CHN2 | missense | NM_001293076 | p.S114L | 0 | 26.2 | 33% | 18 | 9 | 0% | 134 | 0 | 0% | 3 | 0 |
| S1 | 2 | 6990031 | C | A | CMPK2 | missense | NM_207315 | p.V434F | 0 | 34 | 27% | 45 | 17 | 0% | 176 | 0 | 0% | 8 | 0 |
| F2 | 3 | 32533314 | A | G | CMTM6 | missense | NM_017801 | p.L68P | 0 | 27.2 | 66% | 15 | 29 | 0% | 70 | 0 | 73% | 31 | 82 |
| F2 | 6 | 55933851 | C | A | COL21A1 | missense | NM_030820 | p.G695V | 0 | 31 | 42% | 46 | 33 | 0% | 70 | 0 | . | 0 | 0 |
| F2 | 19 | 17679346 | T | G | COLGALT1 | missense | NM_024656 | p.I218S | 0 | 21.4 | 24% | 19 | 6 | 0% | 49 | 0 | 41% | 26 | 18 |
| F2 | 15 | 75122625 | C | G | CPLX3 | nonsense | NM_001030005 | p.S136X | 0 | 39 | 86% | 1 | 6 | 0% | 125 | 0 | . | 0 | 0 |
| F4 | 7 | 29070261 | CT | C | CPVL | FS Del | NM_019029 | p.K417fs | 1 | 34 | 37% | 64 | 38 | 0% | 131 | 0 | . | . | . |
| F3 | 13 | 37678856 | G | A | CSNK1A1L | missense | NM_145203 | p.H180Y | 0 | 22.1 | 45% | 109 | 89 | 0% | 210 | 0 | . | . | . |
| S1 | 20 | 18163843 | C | T | CSRP2BP | missense | NM_020536 | p.H629Y | 0 | 23.2 | 32% | 45 | 21 | 0% | 57 | 0 | 22% | 7 | 2 |
| F4 | 8 | 104390460 | G | A | CTHRC1 | missense | NM_001256099 | p.R179H | 1 | 35 | 40% | 31 | 21 | 0% | 152 | 0 | . | . | . |
| S1 | X | 119675485 | A | C | CUL4B | missense | NM_001079872 | p.L472R | 0 | 28.5 | 29% | 156 | 65 | 0% | 147 | 0 | 0% | 76 | 0 |
| F3 | 4 | 49005993 | A | T | CWH43 | missense | NM_001286791 | p.R321S | 0 | 20.6 | 59% | 16 | 23 | 0% | 112 | 0 | . | . | . |
| F2 | 8 | 65527645 | T | G | CYP7B1 | missense | NM_004820 | p.K332T | 1 | 23.4 | 36% | 27 | 15 | 0% | 45 | 0 | 0% | 3 | 0 |
| F4 | 13 | 36700208 | G | A | DCLK1 | nonsense | NM_004734 | p.R23X | 0 | 35 | 44% | 18 | 14 | 0% | 44 | 0 | . | . | . |
| F2 | 15 | 51743842 | C | G | DMXL2 | missense | NM_001174117 | p.G2259R | 0 | 32 | 91% | 3 | 30 | 0% | 57 | 0 | 17% | 5 | 1 |
| F3 | 3 | 52394359 | G | A | DNAH1 | missense | NM_015512 | p.R1535H | 0 | 34 | 43% | 53 | 40 | 0% | 93 | 0 | . | . | . |
| S1 | 7 | 21751462 | G | T | DNAH11 | missense | NM_001277115 | p.D2323Y | 0 | 34 | 32% | 55 | 26 | 0% | 89 | 0 | . | 0 | 0 |
| S1 | 2 | 84811252 | C | T | DNAH6 | missense | NM_001370 | p.R787W | 0 | 34 | 29% | 65 | 27 | 0% | 191 | 0 | . | 0 | 0 |
| F3 | 5 | 169507282 | T | C | DOCK2 | missense | NM_004946 | p.I1761T | 0 | 25 | 46% | 21 | 18 | 0% | 57 | 0 | . | . | . |
| F4 | 8 | 25234861 | A | G | DOCK5 | missense | NM_024940 | p.D1286G | 0 | 22.4 | 42% | 73 | 53 | 0% | 99 | 0 | . | . | . |
| F2 | 2 | 74005447 | A | C | DUSP11 | missense | NM_003584 | p.F100C | 0 | 24.6 | 23% | 31 | 9 | 0% | 76 | 0 | 29% | 74 | 30 |
| S3 | 3 | 96706535 | G | A | EPHA6 | missense | NM_001080448 | p.G271E | 0 | 26.4 | 16% | 257 | 48 | 0% | 95 | 0 | . | 0 | 0 |
| S3 | 3 | 55768927 | C | T | ERC2 | missense | NM_015576 | p.A860T | 0 | 34 | 15% | 57 | 10 | 0% | 213 | 0 | . | 0 | 0 |
| F4 | 12 | 56526099 | C | T | ESYT1 | missense | NM_001184796 | p.R323C | 0 | 35 | 43% | 31 | 23 | 0% | 349 | 0 | . | . | . |
| F2 | 1 | 169519934 | G | A | F5 | missense | NM_000130 | p.P447L | 0 | 31 | 19% | 44 | 10 | 0% | 51 | 0 | . | 0 | 0 |
| F3 | X | 133922822 | G | A | FAM122B | missense | NM_001166599 | p.P106S | 0 | 25 | 37% | 71 | 41 | 0% | 49 | 0 | . | . | . |
| F4 | 11 | 92620219 | G | T | FAT3 | missense | NM_001008781 | p.G4331C | 0 | 32 | 31% | 52 | 23 | 0% | 67 | 0 | . | . | . |
| S1 | 15 | 76209601 | C | T | FBXO22 | missense | NM_012170 | p.P165L | 0 | 33 | 38% | 43 | 26 | 0% | 190 | 0 | 23% | 24 | 7 |
| F2 | 14 | 24601009 | C | G | FITM1 | missense | NM_203402 | p.I79M | 0 | 23.4 | 50% | 6 | 6 | 0% | 58 | 0 | 0% | 4 | 0 |
| F2 | 17 | 39975902 | C | A | FKBP10 | missense | NM_021939 | p.H346Q | 0 | 26.3 | 50% | 6 | 6 | 0% | 191 | 0 | 0% | 25 | 0 |
| S2 | 6 | 30709938 | G | A | FLOT1 | missense | NM_005803 | p.A10V | 0 | 27.3 | 16% | 31 | 6 | 0% | 43 | 0 | 31% | 142 | 64 |
| S1 | 1 | 240255561 | G | A | FMN2 | missense | NM_001305424 | p.G51E | 0 | 23.7 | 60% | 2 | 3 | 0% | 42 | 0 | . | 0 | 0 |
| S1 | 9 | 37746648 | C | A | FRMPD1 | nonsense | NM_014907 | p.S1540X | 0 | 39 | 33% | 29 | 14 | 0% | 150 | 0 | 0% | 2 | 0 |
| F4 | 12 | 69968288 | A | C | FRS2 | missense | NM_001278357 | p.E360D | 0 | 24.2 | 38% | 38 | 23 | 0% | 93 | 0 | . | . | . |
| S4 | 9 | 108268838 | C | T | FSD1L | nonsense | NM_001287191 | p.R217X | 0 | 36 | 13% | 88 | 13 | 0% | 43 | 0 | 0% | 12 | 0 |
| S1 | 1 | 151062952 | C | T | GABPB2 | missense | NM_144618 | p.A60V | 0 | 32 | 47% | 19 | 17 | 0% | 250 | 0 | 50% | 3 | 3 |
| F2 | 15 | 42618589 | G | T | GANC | nonsense | NM_198141 | p.E463X | 0 | 37 | 80% | 7 | 28 | 0% | 101 | 0 | 100% | 0 | 2 |
| F2 | 9 | 74817526 | T | A | GDA | missense | NM_001242505 | p.H84Q | 0 | 25.5 | 46% | 43 | 37 | 0% | 53 | 0 | . | 0 | 0 |
| F2 | 7 | 150439789 | A | C | GIMAP5 | missense | NM_018384 | p.N188H | 0 | 23.5 | 87% | 4 | 26 | 0% | 97 | 0 | 0% | 5 | 0 |
| S1 | 20 | 5538696 | G | A | GPCPD1 | missense | NM_019593 | p.A575V | 0 | 24.7 | 14% | 32 | 5 | 0% | 235 | 1 | 26% | 46 | 16 |
| F2 | 2 | 10130854 | C | T | GRHL1 | nonsense | NM_198182 | p.R434X | 1 | 41 | 75% | 5 | 15 | 0% | 24 | 0 | . | 0 | 0 |
| S1 | 6 | 146673464 | A | G | GRM1 | missense | NM_001278064 | p.H422R | 0 | 21.7 | 25% | 75 | 25 | 0% | 56 | 0 | . | 0 | 0 |
| F1 | X | 51487878 | C | A | GSPT2 | missense | NM_018094 | p.H386N | 0 | 20.4 | 41% | 73 | 51 | 0% | 64 | 0 | 0% | 65 | 0 |
| S2 | X | 153228882 | T | C | HCFC1 | missense | NM_005334 | p.Y169C | 0 | 25.5 | 81% | 3 | 13 | 0% | 34 | 0 | 88% | 6 | 45 |
| F2 | 19 | 590515 | G | C | HCN2 | missense | NM_001194 | p.E190D | 0 | 26.7 | 71% | 2 | 5 | 0% | 22 | 0 | . | 0 | 0 |
| F4 | X | 6995448 | G | T | HDHD1 | missense | NM_001178135 | p.P108H | 0 | 24 | 25% | 9 | 3 | 0% | 33 | 0 | . | . | . |
| F3 | 17 | 80400068 | G | A | HEXDC | missense | NM_173620 | p.G453E | 0 | 24.9 | 58% | 5 | 7 | 0% | 8 | 0 | . | . | . |
| F2 | 6 | 27782239 | G | A | HIST1H2AJ | missense | NM_021066 | p.L94F | 0 | 28.7 | 31% | 42 | 19 | 0% | 55 | 0 | 0% | 3 | 0 |
| F2 | 6 | 26158486 | G | C | HIST1H2BD | missense | NM_021063 | p.R30P | 0 | 23.2 | 46% | 41 | 35 | 0% | 92 | 0 | 44% | 56 | 44 |
| F1 | 6 | 27839714 | AG | A | HIST1H3I | FS Del | NM_003533 | p.L127fs | 0 | 33 | 43% | 61 | 46 | 0% | 112 | 0 | 0% | 2 | 0 |
| S2 | 1 | 149784977 | C | T | HIST2H3D | missense | NM_001123375 | p.S87N | 0 | 23.5 | 39% | 14 | 9 | 0% | 61 | 0 | 33% | 6 | 3 |
| S1 | X | 135592314 | A | G | HTATSF1 | missense | NM_014500 | p.D333G | 0 | 24.6 | 27% | 137 | 50 | 0% | 81 | 0 | 0% | 57 | 0 |
| S3 | 5 | 63256904 | A | G | HTR1A | missense | NM_000524 | p.Y215H | 1 | 25.6 | 23% | 33 | 10 | 0% | 154 | 0 | . | 0 | 0 |
| F2 | 6 | 160509089 | C | T | IGF2R | missense | NM_000876 | p.S2077F | 0 | 26.6 | 71% | 8 | 20 | 0% | 30 | 0 | 67% | 7 | 14 |
| F2 | 2 | 217528667 | A | T | IGFBP2 | missense | NM_000597 | p.K273M | 0 | 29.9 | 50% | 5 | 5 | 0% | 23 | 0 | 2% | 54 | 1 |
| F1 | 11 | 18730943 | G | A | IGSF22 | missense | NM_173588 | p.P997S | 0 | 23 | 60% | 23 | 35 | 1% | 67 | 1 | . | 0 | 0 |
| F2 | 11 | 133790951 | C | T | IGSF9B | missense | NM_001277285 | p.R890H | 1 | 32 | 91% | 3 | 31 | 0% | 139 | 0 | . | 0 | 0 |
| S1 | 2 | 219922259 | C | T | IHH | missense | NM_002181 | p.R158H | 0 | 34 | 23% | 10 | 3 | 0% | 89 | 0 | . | 0 | 0 |
| F1 | 15 | 41371955 | T | A | INO80 | nonsense | NM_017553 | p.K359X | 0 | 37 | 56% | 78 | 101 | 0% | 296 | 0 | 0% | 3 | 0 |
| S3 | 11 | 62415979 | G | A | INTS5 | nonsense | NM_030628 | p.R525X | 0 | 35 | 38% | 15 | 9 | 0% | 67 | 0 | 40% | 28 | 19 |
| F1 | 1 | 110774858 | G | A | KCNC4 | missense | NM_004978 | p.R612H | 0 | 23.5 | 63% | 7 | 12 | 0% | 79 | 0 | 75% | 3 | 9 |
| F1 | 8 | 36693828 | A | T | KCNU1 | missense | NM_001031836 | p.K437M | 0 | 26 | 24% | 112 | 35 | 0% | 39 | 0 | . | 0 | 0 |
| F2 | 6 | 24596496 | C | T | KIAA0319 | missense | NM_001168376 | p.D91N | 0 | 22.8 | 31% | 33 | 15 | 0% | 76 | 0 | . | 0 | 0 |
| S3 | 16 | 15729776 | G | A | KIAA0430 | missense | NM_001184998 | p.H190Y | 0 | 24.7 | 26% | 40 | 14 | 1% | 170 | 1 | 29% | 27 | 11 |
| F3 | 20 | 16485145 | G | A | KIF16B | missense | NM_001199865 | p.R350C | 0 | 33 | 39% | 68 | 43 | 0% | 74 | 0 | . | . | . |
| S2 | 6 | 129498942 | C | A | LAMA2 | nonsense | NM_000426 | p.C466X | 0 | 32 | 35% | 20 | 11 | 0% | 74 | 0 | 0% | 4 | 0 |
| F3 | 9 | 133901860 | G | A | LAMC3 | missense | NM_006059 | p.A188T | 0 | 26.1 | 35% | 11 | 6 | 0% | 175 | 0 | . | . | . |
| F2 | 15 | 71125361 | C | T | LARP6 | missense | NM_018357 | p.R169K | 0 | 29.1 | 95% | 1 | 19 | 0% | 38 | 0 | . | 0 | 0 |
| F2 | 1 | 66067113 | G | C | LEPR | missense | NM_001198687 | p.G345R | 0 | 25.6 | 87% | 4 | 27 | 0% | 149 | 0 | . | 0 | 0 |
| F2 | 2 | 48915599 | G | A | LHCGR | missense | NM_000233 | p.T446I | 0 | 26.2 | 66% | 28 | 54 | 0% | 93 | 0 | . | 0 | 0 |
| F4 | 19 | 48643240 | C | A | LIG1 | missense | NM_001289064 | p.A291S | 0 | 26.4 | 31% | 24 | 11 | 0% | 53 | 0 | . | . | . |
| F4 | 7 | 141816687 | T | A | LOC93432 | missense | NM_001293626 | p.L5H | 0 | 26.7 | 51% | 52 | 54 | 0% | 53 | 0 | . | . | . |
| S2 | 1 | 70509697 | G | T | LRRC7 | missense | NM_020794 | p.A1306S | 0 | 23.3 | 15% | 41 | 7 | 0% | 185 | 0 | . | 0 | 0 |
| S4 | 2 | 238664750 | G | A | LRRFIP1 | missense | NM_001137551 | p.E167K | 0 | 24.2 | 11% | 25 | 3 | 0% | 15 | 0 | 0% | 293 | 1 |
| F2 | 7 | 110763930 | A | G | LRRN3 | missense | NM_018334 | p.N368D | 0 | 25.7 | 31% | 74 | 34 | 0% | 347 | 0 | 0% | 2 | 0 |
| F2 | 2 | 77745650 | G | A | LRRTM4 | missense | NM_001282928 | p.R450C | 0 | 26.7 | 33% | 108 | 53 | 0% | 138 | 0 | . | 0 | 0 |
| F2 | 12 | 1943730 | G | T | LRTM2 | missense | NM_001039029 | p.G319V | 0 | 24.3 | 62% | 5 | 8 | 0% | 47 | 0 | 0% | 1 | 0 |
| S1 | 8 | 20107365 | C | T | LZTS1 | missense | NM_021020 | p.M553I | 0 | 24.2 | 29% | 15 | 6 | 0% | 149 | 0 | 0% | 1 | 0 |
| F2 | X | 77086359 | A | T | MAGT1 | nonsense | NM_032121 | p.L344X | 0 | 47 | 100% | 0 | 42 | 0% | 49 | 0 | 95% | 2 | 41 |
| F4 | X | 149638387 | A | C | MAMLD1 | missense | NM_001177466 | p.E156A | 0 | 24.9 | 41% | 36 | 25 | 0% | 53 | 0 | . | . | . |
| S1 | 1 | 220825333 | C | T | MARK1 | missense | NM_001286128 | p.T504M | 0 | 22.2 | 19% | 54 | 13 | 0% | 29 | 0 | 33% | 4 | 2 |
| F1 | X | 138679655 | A | C | MCF2 | missense | NM_001171877 | p.N634K | 0 | 26.8 | 42% | 97 | 71 | 0% | 180 | 0 | . | 0 | 0 |
| F4 | 5 | 126754899 | T | C | MEGF10 | missense | NM_001256545 | p.S465P | 0 | 25.6 | 46% | 33 | 28 | 0% | 121 | 0 | . | . | . |
| S2 | 4 | 170913179 | C | T | MFAP3L | missense | NM_001009554 | p.G91S | 0 | 29.3 | 26% | 34 | 12 | 0% | 226 | 0 | . | 0 | 0 |
| F2 | 21 | 26958022 | GGTAGAT | G | MRPL39 | nonFS Del | NM_017446 | p.336_338del | 0 | 20.4 | 35% | 118 | 63 | 0% | 504 | 0 | 24% | 16 | 5 |
| F2 | 19 | 39423174 | G | A | MRPS12 | missense | NM_021107 | p.R84Q | 0 | 23.5 | 20% | 12 | 3 | 0% | 182 | 0 | 45% | 135 | 111 |
| F4 | 11 | 10651219 | A | C | MRVI1 | missense | NM_001098579 | p.L138R | 0 | 28.2 | 33% | 8 | 4 | 0% | 58 | 0 | . | . | . |
| F2 | 8 | 15978036 | C | T | MSR1 | nonsense | NM_138715 | p.W371X | 0 | 43 | 38% | 45 | 27 | 0% | 52 | 0 | 0% | 2 | 0 |
| F4 | 8 | 125579358 | T | C | MTSS1 | missense | NM_001282974 | p.K227R | 0 | 23 | 33% | 31 | 15 | 0% | 34 | 0 | . | . | . |
| F2 | 8 | 17613160 | C | A | MTUS1 | missense | NM_001001924 | p.D53Y | 0 | 23.3 | 19% | 78 | 18 | 0% | 225 | 0 | . | 0 | 0 |
| F2 | 12 | 102045112 | G | A | MYBPC1 | nonsense | NM_001254722 | p.W438X | 0 | 39 | 44% | 83 | 64 | 0% | 62 | 0 | . | 0 | 0 |
| F2 | 13 | 77759266 | A | G | MYCBP2 | missense | NM_015057 | p.L1564P | 0 | 28.3 | 93% | 2 | 25 | 0% | 87 | 0 | 100% | 0 | 3 |
| S1 | 14 | 23870094 | C | T | MYH6 | missense | NM_002471 | p.V412I | 0 | 23.6 | 17% | 24 | 5 | 0% | 118 | 0 | . | 0 | 0 |
| F4 | 2 | 192246236 | C | A | MYO1B | missense | NM_001130158 | p.Q412K | 0 | 22.8 | 39% | 17 | 11 | 0% | 95 | 0 | . | . | . |
| S1 | 18 | 47488721 | A | G | MYO5B | missense | NM_001080467 | p.L487P | 0 | 29.5 | 26% | 32 | 11 | 0% | 48 | 0 | 0% | 3 | 0 |
| F2 | 3 | 172351855 | T | G | NCEH1 | missense | NM_001146278 | p.K80Q | 0 | 23.1 | 31% | 33 | 15 | 0% | 222 | 0 | 20% | 4 | 1 |
| F2 | 3 | 120315208 | T | G | NDUFB4 | missense | NM_001168331 | p.M1R | 0 | 26.9 | 50% | 10 | 10 | 0% | 31 | 0 | 23% | 27 | 8 |
| S4 | 10 | 21141538 | T | A | NEBL | missense | NM_006393 | p.Y315F | 0 | 26 | 15% | 17 | 3 | 0% | 33 | 0 | . | 0 | 0 |
| F4 | 8 | 91929828 | A | C | NECAB1 | missense | NM_022351 | p.T156P | 0 | 26.5 | 38% | 45 | 28 | 0% | 103 | 0 | . | . | . |
| F2 | 14 | 35872940 | T | A | NFKBIA | nonsense | NM_020529 | p.K98X | 0 | 35 | 62% | 5 | 8 | 0% | 49 | 0 | 18% | 243 | 54 |
| F4 | 20 | 34285610 | C | T | NFS1 | missense | NM_001198989 | p.R107H | 1 | 35 | 46% | 19 | 16 | 0% | 135 | 0 | . | . | . |
| S3 | 17 | 7318050 | C | T | NLGN2 | missense | NM_020795 | p.R243C | 0 | 33 | 24% | 16 | 5 | 0% | 209 | 0 | 0% | 20 | 0 |
| F2 | 12 | 117657944 | C | T | NOS1 | missense | NM_001204213 | p.R1033H | 0 | 23.6 | 47% | 31 | 28 | 0% | 17 | 0 | . | 0 | 0 |
| F4 | 8 | 120435218 | A | T | NOV | missense | NM_002514 | p.K307I | 0 | 25.3 | 45% | 47 | 38 | 0% | 97 | 0 | . | . | . |
| F4 | 2 | 101541733 | T | C | NPAS2 | missense | NM_002518 | p.I53T | 0 | 27 | 11% | 51 | 6 | 0% | 124 | 0 | . | . | . |
| S4 | 1 | 5993293 | T | G | NPHP4 | missense | NM_015102 | p.T406P | 0 | 21.8 | 10% | 38 | 4 | 0% | 16 | 0 | 0% | 7 | 0 |
| S1 | 1 | 107867124 | G | A | NTNG1 | missense | NM_001113226 | p.R156H | 0 | 29.7 | 24% | 90 | 28 | 1% | 139 | 1 | 50% | 6 | 6 |
| F4 | X | 128710306 | A | G | OCRL | missense | NM_000276 | p.D631G | 0 | 26.1 | 93% | 2 | 27 | 0% | 61 | 0 | . | . | . |
| S4 | 9 | 138011653 | G | A | OLFM1 | missense | NM_001282611 | p.G363R | 0 | 32 | 17% | 19 | 4 | 0% | 123 | 0 | 0% | 15 | 0 |
| S3 | 18 | 55143876 | G | A | ONECUT2 | missense | NM_004852 | p.R479H | 0 | 34 | 16% | 42 | 8 | 0% | 125 | 0 | . | 0 | 0 |
| S2 | 1 | 248402780 | C | T | OR2M4 | missense | NM_017504 | p.L184F | 0 | 26.6 | 39% | 60 | 38 | 0% | 300 | 0 | . | 0 | 0 |
| S1 | 14 | 20483046 | A | G | OR4K14 | missense | NM_001004712 | p.F103L | 0 | 21 | 29% | 47 | 19 | 0% | 229 | 0 | . | 0 | 0 |
| F3 | 11 | 5020498 | G | T | OR51L1 | missense | NM_001004755 | p.A96S | 0 | 21.7 | 43% | 66 | 50 | 0% | 410 | 0 | . | . | . |
| F3 | 11 | 55587671 | C | A | OR5D18 | missense | NM_001001952 | p.S189Y | 0 | 23.7 | 38% | 68 | 41 | 0% | 319 | 0 | . | . | . |
| S1 | 11 | 56344359 | A | C | OR5M10 | missense | NM_001004741 | p.F280C | 0 | 24.6 | 19% | 186 | 45 | 0% | 310 | 0 | . | 0 | 0 |
| F4 | 1 | 158533095 | T | G | OR6P1 | missense | NM_001160325 | p.Q100H | 0 | 23.1 | 37% | 17 | 10 | 0% | 110 | 0 | . | . | . |
| F1 | 1 | 176526290 | G | A | PAPPA2 | missense | NM_020318 | p.V278M | 0 | 25.8 | 51% | 21 | 22 | 0% | 23 | 0 | . | 0 | 0 |
| S3 | 6 | 162475191 | C | T | PARK2 | missense | NM_004562 | p.D184N | 0 | 23.9 | 52% | 21 | 23 | 0% | 42 | 0 | 0% | 1 | 0 |
| S3 | X | 150844526 | C | T | PASD1 | missense | NM_173493 | p.P745S | 0 | 22.4 | 11% | 56 | 7 | 0% | 25 | 0 | . | 0 | 0 |
| S2 | 2 | 242066334 | G | C | PASK | missense | NM_001252119 | p.L666V | 0 | 20.7 | 14% | 44 | 7 | 0% | 87 | 0 | 55% | 39 | 48 |
| F2 | 10 | 55996612 | C | A | PCDH15 | missense | NM_001142767 | p.R282M | 0 | 28.3 | 45% | 53 | 44 | 0% | 56 | 0 | . | 0 | 0 |
| F4 | 5 | 140589069 | A | G | PCDHB12 | missense | NM_018932 | p.D197G | 0 | 23.2 | 33% | 48 | 24 | 1% | 148 | 1 | . | . | . |
| F4 | 5 | 140480733 | T | G | PCDHB3 | missense | NM_018937 | p.L167R | 0 | 24.8 | 56% | 23 | 29 | 0% | 109 | 0 | . | . | . |
| S4 | 5 | 140750983 | A | G | PCDHGB3 | missense | NM_018924 | p.N341S | 0 | 22.2 | 14% | 142 | 23 | 0% | 241 | 0 | 0% | 3 | 0 |
| F3 | 5 | 95734588 | G | A | PCSK1 | missense | NM_000439 | p.A528V | 0 | 29.4 | 36% | 42 | 24 | 0% | 234 | 0 | . | . | . |
| F4 | 1 | 66723525 | A | C | PDE4B | missense | NM_001037340 | p.N173T | 0 | 23 | 48% | 46 | 43 | 0% | 206 | 0 | . | . | . |
| F3 | X | 54989734 | G | A | PFKFB1 | missense | NM_001271804 | p.T60I | 0 | 22.7 | 34% | 81 | 42 | 0% | 185 | 0 | . | . | . |
| F2 | 1 | 230461107 | G | A | PGBD5 | missense | NM_001258311 | p.A443V | 0 | 27.9 | 54% | 56 | 66 | 0% | 22 | 0 | 4% | 27 | 1 |
| S2 | 6 | 144081651 | G | T | PHACTR2 | missense | NM_001100164 | p.A190S | 0 | 23.8 | 21% | 42 | 11 | 0% | 81 | 0 | 33% | 2 | 1 |
| F4 | 6 | 51921543 | A | T | PKHD1 | missense | NM_138694 | p.L549Q | 0 | 25.9 | 13% | 63 | 9 | 0% | 279 | 0 | . | . | . |
| S1 | 1 | 155263312 | G | T | PKLR | missense | NM_000298 | p.L396M | 0 | 25 | 37% | 12 | 7 | 0% | 104 | 0 | . | 0 | 0 |
| F2 | 12 | 19514517 | T | TA | PLEKHA5 | FS Ins | NM_001256787 | p.L978fs | 0 | 28.8 | 32% | 21 | 10 | 0% | 21 | 0 | . | 0 | 0 |
| S3 | 1 | 208252744 | T | C | PLXNA2 | missense | NM_025179 | p.K816R | 0 | 22.4 | 50% | 3 | 3 | 0% | 37 | 0 | 0% | 3 | 0 |
| S1 | 16 | 72166839 | G | A | PMFBP1 | nonsense | NM_031293 | p.Q419X | 0 | 35 | 24% | 51 | 16 | 0% | 212 | 0 | . | 0 | 0 |
| S3 | 10 | 118313291 | G | A | PNLIP | missense | NM_000936 | p.G171D | 0 | 32 | 63% | 6 | 10 | 0% | 34 | 0 | . | 0 | 0 |
| S2 | 19 | 7607932 | G | A | PNPLA6 | missense | NM_001166114 | p.A524T | 1 | 23.7 | 12% | 23 | 3 | 0% | 44 | 0 | 0% | 68 | 0 |
| S2 | 19 | 50910379 | A | C | POLD1 | missense | NM_001256849 | p.Y545S | 0 | 22.3 | 45% | 6 | 5 | 0% | 71 | 0 | 55% | 38 | 47 |
| F2 | 4 | 57889891 | A | T | POLR2B | missense | NM_001303268 | p.T869S | 0 | 26.3 | 44% | 40 | 32 | 1% | 137 | 1 | 44% | 125 | 98 |
| F3 | 9 | 71628015 | C | T | PRKACG | missense | NM_002732 | p.E332K | 0 | 21.3 | 48% | 28 | 26 | 0% | 53 | 0 | . | . | . |
| S1 | 1 | 227081812 | G | A | PSEN2 | missense | NM_000447 | p.V393M | 1 | 31 | 20% | 12 | 3 | 0% | 37 | 0 | 15% | 23 | 4 |
| F2 | 1 | 97278444 | A | T | PTBP2 | missense | NM_001300990 | p.I398F | 0 | 27.1 | 90% | 4 | 35 | 0% | 268 | 0 | 100% | 0 | 6 |
| F4 | 10 | 129877931 | T | C | PTPRE | missense | NM_130435 | p.L609P | 0 | 25.7 | 13% | 46 | 7 | 0% | 63 | 0 | . | . | . |
| F4 | 1 | 44069099 | A | C | PTPRF | missense | NM_130440 | p.S776R | 0 | 22.8 | 33% | 16 | 8 | 0% | 50 | 0 | . | . | . |
| F4 | 12 | 71095096 | A | C | PTPRR | missense | NM_130846 | p.S94A | 0 | 27.6 | 52% | 26 | 28 | 0% | 154 | 0 | . | . | . |
| S1 | 20 | 40710653 | C | A | PTPRT | missense | NM_007050 | p.G1381W | 0 | 33 | 43% | 23 | 17 | 0% | 48 | 0 | 0% | 2 | 0 |
| F2 | 1 | 158906833 | A | T | PYHIN1 | nonsense | NM_152501 | p.K45X | 0 | 36 | 57% | 38 | 50 | 0% | 166 | 0 | . | 0 | 0 |
| F3 | X | 102192812 | T | G | RAB40AL | missense | NM_001031834 | p.V189G | 0 | 23.9 | 10% | 55 | 6 | 0% | 21 | 0 | . | . | . |
| F2 | 16 | 53487502 | T | C | RBL2 | missense | NM_005611 | p.I302T | 0 | 26.3 | 34% | 60 | 31 | 0% | 154 | 0 | 48% | 13 | 12 |
| S1 | 10 | 6148109 | G | A | RBM17 | missense | NM_001145547 | p.R138H | 0 | 22.3 | 36% | 21 | 12 | 0% | 96 | 0 | 41% | 77 | 54 |
| S1 | 1 | 8418304 | G | A | RERE | missense | NM_001042682 | p.H877Y | 0 | 23.7 | 33% | 10 | 5 | 0% | 34 | 0 | 47% | 50 | 44 |
| S4 | 15 | 56387528 | G | A | RFX7 | nonsense | NM_022841 | p.Q800X | 0 | 38 | 21% | 190 | 50 | 0% | 186 | 0 | 40% | 31 | 21 |
| S1 | X | 71350711 | T | G | RGAG4 | missense | NM_001024455 | p.E227A | 0 | 23.4 | 24% | 53 | 17 | 0% | 67 | 0 | . | 0 | 0 |
| S1 | 13 | 25373578 | G | A | RNF17 | missense | NM_001184993 | p.R482Q | 0 | 34 | 26% | 72 | 25 | 0% | 24 | 0 | . | 0 | 0 |
| F2 | 9 | 104314701 | C | T | RNF20 | nonsense | NM_019592 | p.Q523X | 0 | 41 | 39% | 94 | 60 | 0% | 156 | 0 | 5% | 38 | 2 |
| S1 | 11 | 124738881 | G | A | ROBO3 | missense | NM_022370 | p.R115H | 1 | 29.6 | 38% | 5 | 3 | 0% | 46 | 0 | . | 0 | 0 |
| S4 | 12 | 113303233 | G | A | RPH3A | missense | NM_014954 | p.R78H | 1 | 33 | 11% | 56 | 7 | 0% | 42 | 0 | . | 0 | 0 |
| F2 | 14 | 47120938 | A | G | RPL10L | missense | NM_080746 | p.M1T | 0 | 23.6 | 40% | 33 | 22 | 0% | 192 | 0 | . | 0 | 0 |
| F4 | 17 | 78857639 | A | G | RPTOR | missense | NM_020761 | p.H570R | 0 | 24.3 | 30% | 19 | 8 | 0% | 142 | 0 | . | . | . |
| F4 | 7 | 92760555 | A | C | SAMD9L | missense | NM_001303500 | p.L1577R | 0 | 24.9 | 33% | 45 | 22 | 0% | 168 | 0 | . | . | . |
| F2 | 2 | 224462479 | A | C | SCG2 | missense | NM_003469 | p.L508V | 0 | 20.4 | 26% | 70 | 25 | 0% | 129 | 0 | . | 0 | 0 |
| F4 | 17 | 62021133 | C | G | SCN4A | missense | NM_000334 | p.K1330N | 0 | 27.2 | 32% | 32 | 15 | 0% | 62 | 0 | . | . | . |
| S3 | 2 | 167262870 | A | T | SCN7A | missense | NM_002976 | p.S1423R | 0 | 24.4 | 56% | 117 | 151 | 0% | 155 | 0 | . | 0 | 0 |
| F2 | 7 | 4026936 | G | A | SDK1 | missense | NM_152744 | p.V705M | 1 | 25.1 | 37% | 49 | 29 | 0% | 60 | 0 | 6% | 17 | 1 |
| S3 | 7 | 83640560 | T | G | SEMA3A | missense | NM_006080 | p.K288N | 0 | 26.9 | 35% | 71 | 38 | 0% | 120 | 0 | 0% | 2 | 0 |
| F2 | 9 | 92011673 | G | C | SEMA4D | missense | NM_001142287 | p.F131L | 0 | 27.3 | 50% | 3 | 3 | 0% | 85 | 0 | 29% | 5 | 2 |
| S1 | 22 | 26707754 | T | C | SEZ6L | missense | NM_001184773 | p.Y568H | 0 | 26.7 | 21% | 95 | 25 | 0% | 98 | 0 | . | 0 | 0 |
| S4 | 22 | 40801223 | C | T | SGSM3 | missense | NM_001301849 | p.R122C | 0 | 35 | 38% | 5 | 3 | 0% | 56 | 0 | 26% | 51 | 18 |
| S1 | 17 | 7535063 | C | T | SHBG | nonsense | NM_001040 | p.R238X | 1 | 37 | 31% | 85 | 39 | 0% | 110 | 0 | 0% | 1 | 0 |
| F2 | 15 | 75702525 | C | A | SIN3A | missense | NM_001145357 | p.D371Y | 0 | 33 | 68% | 10 | 21 | 0% | 154 | 0 | 38% | 8 | 5 |
| F2 | 15 | 48512934 | T | G | SLC12A1 | missense | NM_000338 | p.V175G | 0 | 27.9 | 89% | 20 | 156 | 0% | 65 | 0 | . | 0 | 0 |
| S3 | 13 | 99374120 | C | T | SLC15A1 | missense | NM_005073 | p.G135R | 0 | 32 | 20% | 43 | 11 | 0% | 117 | 0 | . | 0 | 0 |
| S4 | X | 118603961 | CT | C | SLC25A5 | FS Del | NM_001152 | p.A150fs | 0 | 23 | 36% | 50 | 28 | 0% | 16 | 0 | 0% | 1353 | 0 |
| F4 | 2 | 220501508 | T | C | SLC4A3 | missense | NM_005070 | p.V816A | 0 | 27 | 41% | 34 | 24 | 0% | 134 | 0 | . | . | . |
| F2 | 12 | 51851197 | C | T | SLC4A8 | missense | NM_001039960 | p.R213W | 0 | 24.8 | 40% | 33 | 22 | 0% | 22 | 0 | . | 0 | 0 |
| F2 | 21 | 35468615 | C | G | SLC5A3 | missense | NM_006933 | p.A373G | 0 | 25.8 | 43% | 32 | 24 | 0% | 170 | 0 | 43% | 4 | 3 |
| F4 | 12 | 20858926 | A | C | SLCO1C1 | missense | NM_017435 | p.K105N | 0 | 26.4 | 41% | 101 | 70 | 0% | 95 | 0 | . | . | . |
| S2 | 9 | 2088540 | G | A | SMARCA2 | missense | NM_001289396 | p.R937H | 0 | 34 | 23% | 103 | 30 | 0% | 218 | 0 | 45% | 117 | 96 |
| F2 | 18 | 19209036 | CAGAGGA | C | SNRPD1 | nonFS Del | NM_001291916 | p.59_60del | 0 | 23.1 | 31% | 42 | 19 | 0% | 119 | 0 | 11% | 585 | 75 |
| S1 | 8 | 101624234 | C | T | SNX31 | missense | NM_152628 | p.R202Q | 1 | 33 | 27% | 24 | 9 | 0% | 49 | 0 | . | 0 | 0 |
| S1 | 10 | 108371744 | G | C | SORCS1 | missense | NM_001013031 | p.N986K | 0 | 27 | 32% | 13 | 6 | 0% | 56 | 0 | . | 0 | 0 |
| F2 | 2 | 39213296 | T | C | SOS1 | missense | NM_005633 | p.D1224G | 0 | 23.9 | 69% | 33 | 72 | 0% | 57 | 0 | 53% | 15 | 17 |
| S4 | 1 | 118628550 | G | A | SPAG17 | missense | NM_206996 | p.T586M | 0 | 29.7 | 14% | 55 | 9 | 0% | 62 | 0 | . | 0 | 0 |
| S2 | 14 | 65271803 | G | A | SPTB | missense | NM_000347 | p.R52W | 0 | 34 | 21% | 19 | 5 | 0% | 38 | 0 | . | 0 | 0 |
| F2 | 2 | 107423273 | G | A | ST6GAL2 | missense | NM_001142351 | p.P484L | 0 | 33 | 46% | 15 | 13 | 0% | 155 | 0 | . | 0 | 0 |
| F3 | 2 | 192011451 | G | A | STAT4 | missense | NM_001243835 | p.T54M | 2 | 24.5 | 39% | 28 | 18 | 1% | 126 | 1 | . | . | . |
| F2 | 17 | 40447750 | G | T | STAT5A | missense | NM_001288719 | p.K133N | 1 | 28.4 | 54% | 6 | 7 | 0% | 106 | 0 | 15% | 17 | 3 |
| F2 | 9 | 125898726 | T | G | STRBP | missense | NM_001171137 | p.R508S | 0 | 27.7 | 49% | 41 | 39 | 0% | 132 | 0 | 46% | 161 | 137 |
| F2 | 19 | 11486687 | C | T | SWSAP1 | missense | NM_175871 | p.P229S | 0 | 23.5 | 57% | 3 | 4 | 0% | 84 | 0 | 44% | 9 | 7 |
| F2 | 10 | 135371401 | A | T | SYCE1 | missense | NM_001143763 | p.L114Q | 0 | 23.7 | 52% | 11 | 12 | 0% | 41 | 0 | . | 0 | 0 |
| F4 | 11 | 6633018 | C | CG | TAF10 | FS Ins | NM_006284 | p.P88fs | 0 | 26.3 | 31% | 9 | 4 | 0% | 75 | 0 | . | . | . |
| S3 | 10 | 8051115 | C | T | TAF3 | missense | NM_031923 | p.P797L | 0 | 23.9 | 50% | 4 | 4 | 0% | 16 | 0 | 40% | 3 | 2 |
| F1 | 1 | 150477198 | C | CG | TARS2 | FS Ins | NM_001271896 | p.C473fs | 0 | 35 | 31% | 87 | 39 | 0% | 348 | 0 | 13% | 49 | 7 |
| F2 | 12 | 10978300 | A | T | TAS2R10 | missense | NM_023921 | p.L190Q | 0 | 23.2 | 43% | 62 | 47 | 0% | 127 | 0 | . | 0 | 0 |
| S1 | 6 | 37250089 | A | C | TBC1D22B | missense | NM_017772 | p.T184P | 0 | 22.7 | 17% | 25 | 5 | 0% | 10 | 0 | 17% | 5 | 1 |
| S2 | 6 | 134210775 | G | T | TCF21 | missense | NM_003206 | p.Q80H | 0 | 25.6 | 47% | 9 | 8 | 0% | 113 | 0 | . | 0 | 0 |
| S4 | 1 | 152082181 | C | T | TCHH | missense | NM_007113 | p.R1171H | 1 | 21.1 | 13% | 56 | 8 | 0% | 244 | 0 | . | 0 | 0 |
| S3 | 1 | 154516476 | C | T | TDRD10 | nonsense | NM_001098475 | p.R181X | 2 | 35 | 33% | 31 | 15 | 0% | 174 | 0 | 0% | 7 | 0 |
| F2 | 4 | 48143383 | A | C | TEC | missense | NM_003215 | p.F508L | 0 | 25.6 | 39% | 31 | 20 | 0% | 251 | 0 | 56% | 4 | 5 |
| F4 | 7 | 115897507 | T | G | TES | missense | NM_015641 | p.S413A | 0 | 25.6 | 17% | 15 | 3 | 0% | 74 | 0 | . | . | . |
| F4 | 3 | 133476703 | T | G | TF | missense | NM_001063 | p.F321V | 0 | 23.1 | 12% | 52 | 7 | 0% | 249 | 0 | . | . | . |
| F3 | 3 | 30732958 | A | G | TGFBR2 | missense | NM_003242 | p.D524G | 0 | 34 | 31% | 18 | 8 | 0% | 19 | 0 | . | . | . |
| F2 | 15 | 43552685 | G | A | TGM5 | missense | NM_004245 | p.R35W | 2 | 26.8 | 87% | 3 | 20 | 0% | 104 | 0 | . | 0 | 0 |
| F2 | 11 | 111956155 | T | G | TIMM8B | missense | NM_012459 | p.K54T | 0 | 21.8 | 36% | 14 | 8 | 0% | 18 | 0 | 50% | 136 | 137 |
| S1 | 10 | 98336579 | T | C | TM9SF3 | missense | NM_020123 | p.D37G | 0 | 20.4 | 31% | 35 | 16 | 0% | 76 | 0 | 31% | 11 | 5 |
| F4 | 12 | 124071408 | T | C | TMED2 | missense | NM_006815 | p.S99P | 0 | 28.2 | 56% | 7 | 9 | 0% | 63 | 0 | . | . | . |
| S1 | 3 | 111797663 | G | A | TMPRSS7 | missense | NM_001042575 | p.G641R | 0 | 24.3 | 31% | 27 | 12 | 0% | 84 | 0 | . | 0 | 0 |
| F1 | 1 | 175046624 | G | C | TNN | missense | NM_022093 | p.A24P | 0 | 24.7 | 10% | 45 | 5 | 1% | 73 | 1 | 0% | 1 | 0 |
| F3 | 9 | 132571627 | T | A | TOR1B | missense | NM_014506 | p.L259Q | 0 | 32 | 35% | 15 | 8 | 0% | 63 | 0 | . | . | . |
| F4 | 6 | 83075058 | G | A | TPBG | missense | NM_001166392 | p.G127D | 0 | 22.9 | 60% | 4 | 6 | 0% | 150 | 0 | . | . | . |
| F4 | 13 | 103330587 | T | G | TPP2 | nonsense | NM_003291 | p.L1224X | 0 | 43 | 42% | 62 | 44 | 0% | 105 | 0 | . | . | . |
| F4 | 3 | 36931332 | A | C | TRANK1 | missense | NM_014831 | p.Y255D | 0 | 24.2 | 46% | 13 | 11 | 0% | 41 | 0 | . | . | . |
| F4 | 4 | 189068181 | A | C | TRIML1 | missense | NM_178556 | p.E354D | 1 | 22.3 | 34% | 27 | 14 | 0% | 113 | 0 | . | . | . |
| F4 | 15 | 99696381 | T | G | TTC23 | missense | NM_001288616 | p.H372P | 0 | 23.5 | 10% | 27 | 3 | 0% | 94 | 0 | . | . | . |
| F4 | 9 | 124736371 | G | T | TTLL11 | missense | NM_001139442 | p.S556R | 0 | 26.5 | 40% | 18 | 12 | 0% | 28 | 0 | . | . | . |
| F3 | X | 118708943 | G | A | UBE2A | missense | NM_001282161 | p.G9R | 0 | 34 | 50% | 5 | 5 | 0% | 38 | 0 | . | . | . |
| S4 | 4 | 115597155 | A | G | UGT8 | missense | NM_003360 | p.Y446C | 0 | 25.7 | 23% | 63 | 19 | 0% | 139 | 0 | 70% | 3 | 7 |
| F3 | 9 | 6413503 | G | A | UHRF2 | missense | NM_152896 | p.V5I | 0 | 23.8 | 63% | 3 | 5 | 0% | 68 | 0 | . | . | . |
| F4 | 19 | 17366369 | T | C | USHBP1 | missense | NM_001297703 | p.E442G | 0 | 25.1 | 40% | 6 | 4 | 0% | 98 | 0 | . | . | . |
| S2 | 2 | 61456017 | T | A | USP34 | missense | NM_014709 | p.H2438L | 0 | 29.2 | 37% | 39 | 23 | 0% | 175 | 0 | 32% | 47 | 22 |
| F4 | Y | 14954231 | T | C | USP9Y | missense | NM_004654 | p.V2093A | 0 | 20.3 | 84% | 9 | 48 | 0% | 73 | 0 | . | . | . |
| F3 | 6 | 30893706 | ACAAGTT | A | VARS2 | nonFS Del | NM_001167733 | p.864_866del | 0 | 38 | 54% | 18 | 21 | 0% | 77 | 0 | . | . | . |
| F2 | 6 | 133073678 | A | G | VNN2 | missense | NM_004665 | p.F250L | 0 | 21.4 | 51% | 34 | 36 | 0% | 110 | 0 | 55% | 85 | 102 |
| S2 | 3 | 51457360 | G | A | VPRBP | missense | NM_001171904 | p.P968S | 0 | 25.8 | 30% | 103 | 45 | 0% | 268 | 0 | 42% | 33 | 24 |
| S1 | 7 | 12401006 | T | A | VWDE | nonsense | NM_001135924 | p.K1014X | 0 | 38 | 15% | 76 | 13 | 0% | 89 | 0 | . | 0 | 0 |
| F2 | 12 | 49361851 | C | T | WNT10B | missense | NM_003394 | p.G197S | 0 | 23.1 | 18% | 18 | 4 | 0% | 92 | 0 | . | 0 | 0 |
| F2 | 7 | 44247003 | C | T | YKT6 | missense | NM_006555 | p.S102F | 0 | 24 | 33% | 28 | 14 | 0% | 35 | 0 | 54% | 78 | 90 |
| F2 | 4 | 69198530 | C | T | YTHDC1 | missense | NM_001031732 | p.V337I | 1 | 23.1 | 35% | 32 | 17 | 0% | 106 | 0 | 41% | 26 | 18 |
| S4 | 21 | 43414197 | C | T | ZBTB21 | missense | NM_020727 | p.G3E | 0 | 29.7 | 11% | 66 | 8 | 0% | 204 | 0 | 36% | 7 | 4 |
| F3 | 1 | 6640744 | C | A | ZBTB48 | nonsense | NM_001278647 | p.Y25X | 0 | 36 | 65% | 6 | 11 | 0% | 91 | 0 | . | . | . |
| F2 | 14 | 89073689 | A | C | ZC3H14 | missense | NM_207662 | p.H188P | 0 | 25.1 | 10% | 27 | 3 | 0% | 29 | 0 | 0% | 66 | 0 |
| F3 | 16 | 88694056 | C | A | ZC3H18 | nonsense | NM_144604 | p.S712X | 0 | 41 | 41% | 13 | 9 | 0% | 9 | 0 | . | . | . |
| F2 | 7 | 100001836 | C | T | ZCWPW1 | missense | NM_001258008 | p.R429Q | 0 | 34 | 49% | 29 | 28 | 0% | 146 | 0 | 37% | 104 | 62 |
| S1 | 2 | 145156682 | C | T | ZEB2 | nonsense | NM_001171653 | p.W667X | 0 | 38 | 32% | 56 | 26 | 0% | 147 | 0 | 26% | 28 | 10 |
| F2 | 8 | 135615138 | T | G | ZFAT | missense | NM_001174157 | p.Y213S | 0 | 25.9 | 64% | 23 | 41 | 1% | 88 | 1 | 64% | 5 | 9 |
| S3 | 8 | 106456594 | G | A | ZFPM2 | missense | NM_012082 | p.D96N | 1 | 24.1 | 31% | 45 | 20 | 0% | 23 | 0 | 0% | 4 | 0 |
| F4 | 6 | 27419922 | T | G | ZNF184 | missense | NM_007149 | p.K472N | 0 | 24.6 | 45% | 29 | 24 | 0% | 135 | 0 | . | . | . |
| F2 | 19 | 58050212 | G | T | ZNF549 | nonsense | NM_153263 | p.E601X | 0 | 36 | 56% | 22 | 28 | 0% | 45 | 0 | 0% | 3 | 0 |
| S1 | 19 | 37975237 | G | A | ZNF570 | missense | NM_144694 | p.R238K | 1 | 23.9 | 42% | 144 | 106 | 0% | 113 | 0 | 0% | 4 | 0 |
| F2 | 19 | 52618997 | A | G | ZNF616 | missense | NM_178523 | p.F474L | 0 | 25.6 | 48% | 51 | 48 | 0% | 189 | 0 | 63% | 3 | 5 |
| F2 | 3 | 40529094 | A | T | ZNF619 | missense | NM_001145083 | p.I321F | 0 | 27 | 62% | 30 | 49 | 0% | 125 | 0 | 90% | 1 | 9 |
| F4 | 19 | 55993087 | AC | A | ZNF628 | nonsense | NM_033113 | p.Y176X | 0 | 22.7 | 59% | 9 | 13 | 0% | 173 | 0 | . | . | . |
| S1 | 16 | 30793647 | C | T | ZNF629 | missense | NM_001080417 | p.G668R | 0 | 29.1 | 27% | 11 | 4 | 0% | 58 | 0 | 0% | 1 | 0 |
| F2 | 8 | 146068008 | T | G | ZNF7 | missense | NM_001282797 | p.F410V | 0 | 28 | 26% | 56 | 20 | 0% | 126 | 0 | 29% | 37 | 15 |
| F1 | 19 | 12575177 | C | T | ZNF709 | missense | NM_152601 | p.R520Q | 1 | 23.8 | 42% | 196 | 141 | 0% | 113 | 0 | 50% | 1 | 1 |
| F4 | 7 | 56007680 | C | A | ZNF713 | missense | NM_182633 | p.P438H | 0 | 22.7 | 27% | 30 | 11 | 0% | 143 | 0 | . | . | . |
| S1 | 19 | 22497448 | G | T | ZNF729 | missense | NM_001242680 | p.G410V | 0 | 23.4 | 18% | 167 | 36 | 0% | 52 | 0 | . | 0 | 0 |
| S3 | 10 | 126672126 | G | A | ZRANB1 | missense | NM_017580 | p.E593K | 0 | 32 | 49% | 19 | 18 | 0% | 103 | 0 | 43% | 25 | 19 |
| F3 | 10 | 75550887 | G | A | ZSWIM8 | missense | NM_001242487 | p.A366T | 0 | 23.9 | 38% | 21 | 13 | 0% | 176 | 0 | . | . | . |
| F2 | 3 | 126185085 | G | A | ZXDC | missense | NM_001040653 | p.R452C | 0 | 31 | 33% | 29 | 14 | 0% | 41 | 0 | 35% | 13 | 7 |

**Supplementary table 6. Overrepresented gene sets of differentially expressed genes**

**Down-regulation**

| **Gene Set Name** | **# Genes in Gene Set (K)** | **Description** | **FDR q-value** | **Related Genes** |
| --- | --- | --- | --- | --- |
| **REACTOME IMMUNE SYSTEM** | 933 | Genes involved in Immune System | 5.15E-30 | ITGB2, ITGAL, ITGB1, ITGAV, SELL, SOS1, PRKCQ, RASGRP1, LCK, FYN, LCP2, PRKCB, ITPR2, CD36, PRKACB, YES1, KIF2A, PTPRC, CD28, CD4, CTLA4, ICOS, VCAM1, CDH1, PDCD1LG2, ITK, CD3D, CD3E, CD3G, GRAP2, PAG1, FYB, CBLB, C3, TRAT1, CTSC, NEDD4, CDKN1B, WWP1, CRTAM, CYBB, SEC24D, LNPEP, RAP1GAP2, UBE4A, UBR1, GAB2, IL1R1, IFNAR2, IL6ST, CSF2RB, IL1RAP, STAT1, PTAFR, RPS6KA3, RPS6KA2, C1QB, C1QC, S100B, ADAM17, C1S, C4A, CFH, PELI2, HERC5, EEA1, RPS6KA5, TLR4, TLR7, TLR8, TLR1, TLR5, CYLD, P2RX7, RNF125, TXNIP, EIF4G3, GBP4, GBP5, EIF4E3 |
| **REACTOME HEMOSTASIS** | 466 | Genes involved in Hemostasis | 3.09E-25 | ITGB2, ITGAL, ITGB1, ITGAV, SELL, SOS1, PRKCQ, RASGRP1, LCK, FYN, LCP2, PRKCB, ITPR2, CD36, PRKACB, YES1, KIF2A, SELP, CD2, PRKCE, VEGFC, PIK3R5, GNG12, GNG4, GNG7, GNB4, ARRB1, ADRA2A, P2RY12, F2R, PRKCH, THBS1, A2M, SERPING1, MMRN1, GP1BA, ACTN1, F5, PSAP, CLU, PDE3B, CAV1, THBD, SIRPG, TEK, SLC7A5, DOCK1, GUCY1A3, GUCY1B3, MRVI1, DOCK4, SLC8A1, DOCK9 |
| **KEGG CELL ADHESION MOLECULES CAMS** | 134 | Cell adhesion molecules (CAMs) | 7.71E-19 | ITGB2, ITGAL, ITGB1, ITGAV, SELL, PTPRC, CD28, CD4, CTLA4, ICOS, VCAM1, CDH1, PDCD1LG2, SELP, CD2, ITGA4, ITGA6, ITGA9, SDC3, VCAN, SELE, SELPLG, CADM1, PTPRM, PTPRF, NFASC, CD6 |
| **REACTOME PLATELET ACTIVATION SIGNALING AND AGGREGATION** | 208 | Genes involved in Platelet activation, signaling and aggregation | 7.22E-18 | SOS1, PRKCQ, RASGRP1, LCK, FYN, LCP2, PRKCB, ITPR2, CD36, SELP, PRKCE, VEGFC, PIK3R5, GNG12, GNG4, GNG7, GNB4, ARRB1, ADRA2A, P2RY12, F2R, PRKCH, THBS1, A2M, SERPING1, MMRN1, GP1BA, ACTN1, F5, PSAP, CLU |
| **REACTOME ADAPTIVE IMMUNE SYSTEM** | 539 | Genes involved in Adaptive Immune System | 5.94E-17 | ITGB2, ITGAL, ITGB1, ITGAV, SELL, SOS1, PRKCQ, RASGRP1, LCK, FYN, LCP2, PRKCB, ITPR2, CD36, PRKACB, YES1, KIF2A, PTPRC, CD28, CD4, CTLA4, ICOS, VCAM1, CDH1, PDCD1LG2, ITK, CD3D, CD3E, CD3G, GRAP2, PAG1, FYB, CBLB, C3, TRAT1, CTSC, NEDD4, CDKN1B, WWP1, CRTAM, CYBB, SEC24D, LNPEP, RAP1GAP2, UBE4A, UBR1 |
| **PID TCR PATHWAY** | 66 | TCR signaling in naive CD4+ T cells | 2.43E-16 | SOS1, PRKCQ, RASGRP1, LCK, FYN, LCP2, PRKCB, PTPRC, CD28, CD4, ITK, CD3D, CD3E, CD3G, GRAP2, PAG1, FYB, GAB2, PRKCE |
| **KEGG CYTOKINE CYTOKINE RECEPTOR INTERACTION** | 267 | Cytokine-cytokine receptor interaction | 6.49E-15 | IL1R1, IFNAR2, IL6ST, CSF2RB, IL1RAP, VEGFC, CXCL12, CCR5, CXCR3, CXCL11, CCL19, CCR4, CXCR6, XCR1, CCL2, IL12RB2, CSF1, TNFSF14, TNFSF15, CSF1R, KDR, IL18R1, TNFRSF9, LEPR, LIFR, OSMR, TNFRSF1A, TNFRSF8, ACVR1, TNFRSF11B, CD27 |
| **KEGG T CELL RECEPTOR SIGNALING PATHWAY** | 108 | T cell receptor signaling pathway | 2.65E-13 | SOS1, PRKCQ, RASGRP1, LCK, FYN, LCP2, PTPRC, CD28, CD4, CTLA4, ICOS, ITK, CD3D, CD3E, CD3G, GRAP2, CBLB, PIK3R5, NFAT5, NFATC3 |
| **KEGG CHEMOKINE SIGNALING PATHWAY** | 190 | Chemokine signaling pathway | 3.08E-13 | SOS1, PRKCB, PRKACB, ITK, STAT1, PIK3R5, GNG12, GNG4, GNG7, GNB4, ARRB1, CXCL12, CCR5, CXCR3, CXCL11, CCL19, CCR4, CXCR6, XCR1, CCL2, ADCY7, GRK5, TIAM1, TIAM2, PREX1 |
| **PID CD8 TCR PATHWAY** | 53 | TCR signaling in naive CD8+ T cells | 8.60E-13 | SOS1, PRKCQ, RASGRP1, LCK, FYN, LCP2, PRKCB, PTPRC, CD28, CD3D, CD3E, CD3G, GRAP2, PAG1, PRKCE |
| **REACTOME SIGNALING BY GPCR** | 920 | Genes involved in Signaling by GPCR | 1.49E-11 | SOS1, PRKCQ, RASGRP1, PRKCB, ITPR2, PRKACB, C3, PTAFR, RPS6KA3, RPS6KA2, PRKCE, PIK3R5, GNG12, GNG4, GNG7, GNB4, ADRA2A, P2RY12, F2R, PRKCH, PDE3B, CXCL12, CCR5, CXCR3, CXCL11, CCL19, CCR4, CXCR6, XCR1, CCL2, ADCY7, GRK5, TIAM1, TIAM2, PREX1, C3AR1, LPAR1, FPR2, P2RY13, P2RY14, CNR1, S1PR3, OPN3, EDNRB, LPAR6, PTGER2, CALCRL, RASGRF2, AKAP13, PDE4D, CD97 |
| **BIOCARTA THELPER PATHWAY** | 14 | T Helper Cell Surface Molecules | 1.95E-11 | ITGB2, ITGAL, PTPRC, CD28, CD4, CD3D, CD3E, CD3G, CD2 |
| **REACTOME G ALPHA I SIGNALLING EVENTS** | 195 | Genes involved in G alpha (i) signalling events | 3.00E-11 | C3, GNG12, GNG4, GNG7, GNB4, ADRA2A, P2RY12, CXCL12, CCR5, CXCR3, CXCL11, CCL19, CCR4, CXCR6, ADCY7, C3AR1, LPAR1, FPR2, P2RY13, P2RY14, CNR1, S1PR3, OPN3 |
| **REACTOME GPCR DOWNSTREAM SIGNALING** | 805 | Genes involved in GPCR downstream signaling | 6.74E-11 | SOS1, PRKCQ, RASGRP1, PRKCB, ITPR2, C3, PTAFR, PRKCE, PIK3R5, GNG12, GNG4, GNG7, GNB4, ADRA2A, P2RY12, F2R, PRKCH, PDE3B, CXCL12, CCR5, CXCR3, CXCL11, CCL19, CCR4, CXCR6, XCR1, ADCY7, GRK5, TIAM1, TIAM2, PREX1, C3AR1, LPAR1, FPR2, P2RY13, P2RY14, CNR1, S1PR3, OPN3, EDNRB, LPAR6, PTGER2, CALCRL, RASGRF2, AKAP13, PDE4D |
| **PID CXCR4 PATHWAY** | 102 | CXCR4-mediated signaling events | 1.13E-10 | ITGB1, ITGAV, LCK, FYN, YES1, PTPRC, CD4, CD3D, CD3E, CD3G, PAG1, STAT1, PIK3R5, ITGA4, ITGA6, ITGA9, CXCL12 |
| **BIOCARTA NO2IL12 PATHWAY** | 17 | NO2-dependent IL 12 Pathway in NK cells | 1.70E-10 | CD4, CD3D, CD3E, CD3G, CD2, CCR5, CXCR3, IL12RB2, STAT4 |
| **REACTOME CELL SURFACE INTERACTIONS AT THE VASCULAR WALL** | 91 | Genes involved in Cell surface interactions at the vascular wall | 1.94E-10 | ITGB2, ITGAL, ITGB1, ITGAV, SELL, SOS1, LCK, FYN, YES1, SELP, CD2, CAV1, THBD, SIRPG, TEK, SLC7A5 |
| **REACTOME GPCR LIGAND BINDING** | 408 | Genes involved in GPCR ligand binding | 2.70E-10 | C3, PTAFR, GNG12, GNG4, GNG7, GNB4, ADRA2A, P2RY12, F2R, CXCL12, CCR5, CXCR3, CXCL11, CCL19, CCR4, CXCR6, XCR1, CCL2, C3AR1, LPAR1, FPR2, P2RY13, P2RY14, CNR1, S1PR3, OPN3, EDNRB, LPAR6, PTGER2, CALCRL, CD97 |
| **REACTOME TCR SIGNALING** | 54 | Genes involved in TCR signaling | 2.87E-10 | PRKCQ, LCK, LCP2, PTPRC, CD4, ITK, CD3D, CD3E, CD3G, GRAP2, PAG1, FYB, TRAT1 |
| **NABA MATRISOME** | 1028 | Ensemble of genes encoding extracellular matrix and extracellular matrix-associated proteins | 5.10E-10 | CTSC, C1QB, C1QC, S100B, ADAM17, VEGFC, THBS1, A2M, SERPING1, MMRN1, SDC3, VCAN, CXCL12, CXCL11, CCL19, CCL2, CSF1, TNFSF14, TNFSF15, LAMA3, LAMC2, IGF1, FGF7, HSPG2, SEMA3A, SEMA5A, NID1, IGFBP5, FGL2, EFEMP1, ABI3BP, VWA5A, MATN2, NELL2, LGI2, SPARCL1, RSPO3, COL14A1, SPOCK2, SEMA3D, ADAM28, HPSE, TIMP2, ADAMTS2, ITIH5, CST7, ADAM9, CLEC10A, FCN1, CLEC9A, MEGF9 |
| **BIOCARTA TCYTOTOXIC PATHWAY** | 14 | T Cytotoxic Cell Surface Molecules | 1.00E-09 | ITGB2, ITGAL, PTPRC, CD28, CD3D, CD3E, CD3G, CD2 |
| **KEGG HEMATOPOIETIC CELL LINEAGE** | 88 | Hematopoietic cell lineage | 1.16E-09 | CD36, CD4, CD3D, CD3E, CD3G, IL1R1, CD2, GP1BA, ITGA4, ITGA6, CSF1, CSF1R, CD7, CD5, CD38 |
| **KEGG FOCAL ADHESION** | 201 | Focal adhesion | 1.88E-09 | ITGB1, ITGAV, SOS1, FYN, PRKCB, VEGFC, PIK3R5, THBS1, ACTN1, CAV1, DOCK1, ITGA4, ITGA6, ITGA9, KDR, LAMA3, LAMC2, IGF1, PARVA, MYLK, CCND1 |
| **REACTOME CLASS A1 RHODOPSIN LIKE RECEPTORS** | 305 | Genes involved in Class A/1 (Rhodopsin-like receptors) | 5.01E-09 | C3, PTAFR, ADRA2A, P2RY12, F2R, CXCL12, CCR5, CXCR3, CXCL11, CCL19, CCR4, CXCR6, XCR1, CCL2, C3AR1, LPAR1, FPR2, P2RY13, P2RY14, CNR1, S1PR3, OPN3, EDNRB, LPAR6, PTGER2 |
| **BIOCARTA MONOCYTE PATHWAY** | 11 | Monocyte and its Surface Molecules | 5.76E-09 | ITGB2, ITGAL, ITGB1, SELL, SELP, ITGA4, SELE |
| **KEGG COMPLEMENT AND COAGULATION CASCADES** | 69 | Complement and coagulation cascades | 5.81E-09 | C3, C1QB, C1QC, C1S, C4A, CFH, F2R, A2M, SERPING1, F5, THBD, C3AR1, C4B |
| **BIOCARTA LAIR PATHWAY** | 17 | Cells and Molecules involved in local acute inflammatory response | 6.03E-09 | ITGB2, ITGAL, ITGB1, VCAM1, C3, SELP, ITGA4, SELPLG |
| **ST T CELL SIGNAL TRANSDUCTION** | 45 | T Cell Signal Transduction | 7.40E-09 | SOS1, RASGRP1, LCK, LCP2, PTPRC, CD28, CTLA4, ITK, CD3D, GRAP2, NFAT5 |
| **REACTOME CELL CELL COMMUNICATION** | 120 | Genes involved in Cell-Cell communication | 8.67E-09 | ITGB1, FYN, CDH1, FYB, ACTN1, SIRPG, CADM1, LAMA3, LAMC2, PARVA, IQGAP1, CTNND1, INADL, FBLIM1, CDH11, SIRPB1 |
| **REACTOME GASTRIN CREB SIGNALLING PATHWAY VIA PKC AND MAPK** | 205 | Genes involved in Gastrin-CREB signalling pathway via PKC and MAPK | 1.45E-08 | SOS1, PRKCQ, RASGRP1, ITPR2, PTAFR, RPS6KA3, RPS6KA2, PRKCE, GNG12, GNG4, GNG7, GNB4, F2R, PRKCH, XCR1, GRK5, LPAR1, FPR2, EDNRB, LPAR6 |
| **REACTOME GENERATION OF SECOND MESSENGER MOLECULES** | 27 | Genes involved in Generation of second messenger molecules | 1.46E-08 | LCK, LCP2, CD4, ITK, CD3D, CD3E, CD3G, GRAP2, FYB |
| **REACTOME INNATE IMMUNE SYSTEM** | 279 | Genes involved in Innate Immune System | 1.89E-08 | CD4, C3, RPS6KA3, RPS6KA2, C1QB, C1QC, S100B, C1S, C4A, CFH, PELI2, HERC5, EEA1, RPS6KA5, TLR4, TLR7, TLR8, TLR1, TLR5, CYLD, P2RX7, RNF125, TXNIP |
| **PID IL12 2PATHWAY** | 63 | IL12-mediated signaling events | 2.03E-08 | LCK, CD4, CD3D, CD3E, CD3G, IL1R1, STAT1, CCR5, IL12RB2, IL18R1, STAT4, EOMES |
| **REACTOME COSTIMULATION BY THE CD28 FAMILY** | 63 | Genes involved in Costimulation by the CD28 family | 2.03E-08 | LCK, FYN, YES1, CD28, CD4, CTLA4, ICOS, PDCD1LG2, CD3D, CD3E, CD3G, GRAP2 |
| **BIOCARTA TCRA PATHWAY** | 13 | Lck and Fyn tyrosine kinases in initiation of TCR Activation | 2.08E-08 | LCK, FYN, PTPRC, CD4, CD3D, CD3E, CD3G |
| **KEGG REGULATION OF ACTIN CYTOSKELETON** | 216 | Regulation of actin cytoskeleton | 3.04E-08 | ITGB2, ITGAL, ITGB1, ITGAV, SOS1, PIK3R5, GNG12, F2R, ACTN1, DOCK1, ITGA4, ITGA6, ITGA9, TIAM1, TIAM2, FGF7, MYLK, IQGAP1, NCKAP1, PIKFYVE |
| **KEGG VASCULAR SMOOTH MUSCLE CONTRACTION** | 115 | Vascular smooth muscle contraction | 3.38E-08 | PRKCQ, PRKCB, ITPR2, PRKACB, PRKCE, PRKCH, GUCY1A3, GUCY1B3, MRVI1, ADCY7, CALCRL, MYLK, ACTA2, PPP1R12B, CALD1 |
| **BIOCARTA CTLA4 PATHWAY** | 21 | The Co-Stimulatory Signal During T-cell Activation | 3.38E-08 | LCK, CD28, CTLA4, ICOS, ITK, CD3D, CD3E, CD3G |
| **REACTOME CYTOKINE SIGNALING IN IMMUNE SYSTEM** | 270 | Genes involved in Cytokine Signaling in Immune system | 4.60E-08 | SOS1, LCK, FYN, PRKACB, YES1, VCAM1, NEDD4, GAB2, IL1R1, IFNAR2, IL6ST, CSF2RB, IL1RAP, STAT1, PTAFR, ADAM17, PELI2, HERC5, EIF4G3, GBP4, GBP5, EIF4E3 |
| **BIOCARTA IL12 PATHWAY** | 23 | IL12 and Stat4 Dependent Signaling Pathway in Th1 Development | 7.51E-08 | CD3D, CD3E, CD3G, CCR5, CXCR3, IL12RB2, IL18R1, STAT4 |
| **REACTOME G ALPHA Z SIGNALLING EVENTS** | 44 | Genes involved in G alpha (z) signalling events | 7.54E-08 | PRKCQ, PRKCB, PRKCE, GNG12, GNG4, GNG7, GNB4, ADRA2A, PRKCH, ADCY7 |
| **REACTOME PHOSPHORYLATION OF CD3 AND TCR ZETA CHAINS** | 16 | Genes involved in Phosphorylation of CD3 and TCR zeta chains | 1.11E-07 | LCK, PTPRC, CD4, CD3D, CD3E, CD3G, PAG1 |
| **KEGG ADHERENS JUNCTION** | 75 | Adherens junction | 1.31E-07 | FYN, YES1, CDH1, ACTN1, PTPRM, PTPRF, IQGAP1, CTNND1, PTPRJ, SMAD3, TJP1, SORBS1 |
| **PID PDGFRB PATHWAY** | 129 | PDGFR-beta signaling pathway | 1.45E-07 | ITGAV, SOS1, LCK, FYN, YES1, PAG1, STAT1, RPS6KA3, PRKCE, PIK3R5, DOCK4, IQGAP1, NCKAP1, ACTA2, PTPRJ |
| **REACTOME SIGNALING BY RHO GTPASES** | 113 | Genes involved in Signaling by Rho GTPases | 2.01E-07 | SOS1, A2M, TIAM1, TIAM2, PREX1, RASGRF2, AKAP13, BCR, CHN1, ARHGAP26, DLC1, ARHGAP10, ARHGAP29, STARD8 |
| **BIOCARTA LYM PATHWAY** | 11 | Adhesion and Diapedesis of Lymphocytes | 2.56E-07 | ITGB2, ITGAL, ITGB1, SELL, VCAM1, ITGA4 |
| **BIOCARTA TCAPOPTOSIS PATHWAY** | 11 | HIV Induced T Cell Apoptosis | 2.56E-07 | CD28, CD4, CD3D, CD3E, CD3G, CCR5 |
| **PID RAC1 REG PATHWAY** | 38 | Regulation of RAC1 activity | 2.70E-07 | SOS1, DOCK1, TIAM1, TIAM2, PREX1, RASGRF2, BCR, CHN1, SPATA13 |
| **KEGG PATHWAYS IN CANCER** | 328 | Pathways in cancer | 2.70E-07 | ITGB1, ITGAV, SOS1, PRKCB, CDH1, CBLB, CDKN1B, STAT1, VEGFC, PIK3R5, ITGA6, CSF1R, LAMA3, LAMC2, IGF1, FGF7, CCND1, SMAD3, BCR, HIF1A, DAPK1, BRCA2, EPAS1 |
| **PID CD8 TCR DOWNSTREAM PATHWAY** | 65 | Downstream signaling in naive CD8+ T cells | 2.70E-07 | PRKCQ, PRKCB, CD3D, CD3E, CD3G, IFNAR2, PRKCE, TNFRSF9, NFATC3, STAT4, EOMES |

**Up-regulation**

| **Gene Set Name** | **# Genes in Gene Set (K)** | **Description** | **FDR q-value** | **Related Genes** |
| --- | --- | --- | --- | --- |
| **KEGG RIBOSOME** | 88 | Ribosome | 5.80E-70 | RPS27A, RPS27, RPS6, RPS4X, RPSA, RPS2, RPS3, RPS5, RPS11, RPS12, RPS13, RPS15, RPS15A, RPS18, RPS19, RPS21, RPS24, RPS25, RPS26, RPS28, RPS29, UBA52, RPL23, RPL13A, RPL35, RPL36, RPL7, RPL8, RPL9, RPL10, RPL13, RPL15, RPL18, RPL18A, RPL24, RPL27A, RPL28, RPL29, RPL31, RPL32, RPL34, RPL35A, RPL37, RPL37A, RPL38, RPL39, RPLP0, RPLP1, RPLP2, RPL14 |
| **REACTOME INFLUENZA VIRAL RNA TRANSCRIPTION AND REPLICATION** | 169 | Genes involved in Influenza Viral RNA Transcription and Replication | 4.02E-57 | RPS27A, RPS27, RPS6, RPS4X, RPSA, RPS2, RPS3, RPS5, RPS11, RPS12, RPS13, RPS15, RPS15A, RPS18, RPS19, RPS21, RPS24, RPS25, RPS26, RPS28, RPS29, UBA52, RPL23, RPL13A, RPL35, RPL36, RPL7, RPL8, RPL9, RPL10, RPL13, RPL15, RPL18, RPL18A, RPL24, RPL27A, RPL28, RPL29, RPL31, RPL32, RPL34, RPL35A, RPL37, RPL37A, RPL38, RPL39, RPLP0, RPLP1, RPLP2, RPL14, RPS14, POLR2H, POLR2J |
| **REACTOME PEPTIDE CHAIN ELONGATION** | 153 | Genes involved in Peptide chain elongation | 1.22E-56 | RPS27A, RPS27, RPS6, RPS4X, RPSA, RPS2, RPS3, RPS5, RPS11, RPS12, RPS13, RPS15, RPS15A, RPS18, RPS19, RPS21, RPS24, RPS25, RPS26, RPS28, RPS29, UBA52, RPL23, RPL13A, RPL35, RPL36, RPL7, RPL8, RPL9, RPL10, RPL13, RPL15, RPL18, RPL18A, RPL24, RPL27A, RPL28, RPL29, RPL31, RPL32, RPL34, RPL35A, RPL37, RPL37A, RPL38, RPL39, RPLP0, RPLP1, RPLP2, RPL14, RPS14 |
| **REACTOME 3 UTR MEDIATED TRANSLATIONAL REGULATION** | 176 | Genes involved in 3' -UTR-mediated translational regulation | 2.89E-53 | RPS27A, RPS27, RPS6, RPS4X, RPSA, RPS2, RPS3, RPS5, RPS11, RPS12, RPS13, RPS15, RPS15A, RPS18, RPS19, RPS21, RPS24, RPS25, RPS26, RPS28, RPS29, UBA52, RPL23, RPL13A, RPL35, RPL36, RPL7, RPL8, RPL9, RPL10, RPL13, RPL15, RPL18, RPL18A, RPL24, RPL27A, RPL28, RPL29, RPL31, RPL32, RPL34, RPL35A, RPL37, RPL37A, RPL38, RPL39, RPLP0, RPLP1, RPLP2, RPL14, RPS14 |
| **REACTOME NONSENSE MEDIATED DECAY ENHANCED BY THE EXON JUNCTION COMPLEX** | 176 | Genes involved in Nonsense Mediated Decay Enhanced by the Exon Junction Complex | 2.89E-53 | RPS27A, RPS27, RPS6, RPS4X, RPSA, RPS2, RPS3, RPS5, RPS11, RPS12, RPS13, RPS15, RPS15A, RPS18, RPS19, RPS21, RPS24, RPS25, RPS26, RPS28, RPS29, UBA52, RPL23, RPL13A, RPL35, RPL36, RPL7, RPL8, RPL9, RPL10, RPL13, RPL15, RPL18, RPL18A, RPL24, RPL27A, RPL28, RPL29, RPL31, RPL32, RPL34, RPL35A, RPL37, RPL37A, RPL38, RPL39, RPLP0, RPLP1, RPLP2, RPL14, RPS14 |
| **REACTOME SRP DEPENDENT COTRANSLATIONAL PROTEIN TARGETING TO MEMBRANE** | 179 | Genes involved in SRP-dependent cotranslational protein targeting to membrane | 6.42E-53 | RPS27A, RPS27, RPS6, RPS4X, RPSA, RPS2, RPS3, RPS5, RPS11, RPS12, RPS13, RPS15, RPS15A, RPS18, RPS19, RPS21, RPS24, RPS25, RPS26, RPS28, RPS29, UBA52, RPL23, RPL13A, RPL35, RPL36, RPL7, RPL8, RPL9, RPL10, RPL13, RPL15, RPL18, RPL18A, RPL24, RPL27A, RPL28, RPL29, RPL31, RPL32, RPL34, RPL35A, RPL37, RPL37A, RPL38, RPL39, RPLP0, RPLP1, RPLP2, RPL14, RPS14 |
| **REACTOME INFLUENZA LIFE CYCLE** | 203 | Genes involved in Influenza Life Cycle | 7.24E-53 | RPS27A, RPS27, RPS6, RPS4X, RPSA, RPS2, RPS3, RPS5, RPS11, RPS12, RPS13, RPS15, RPS15A, RPS18, RPS19, RPS21, RPS24, RPS25, RPS26, RPS28, RPS29, UBA52, RPL23, RPL13A, RPL35, RPL36, RPL7, RPL8, RPL9, RPL10, RPL13, RPL15, RPL18, RPL18A, RPL24, RPL27A, RPL28, RPL29, RPL31, RPL32, RPL34, RPL35A, RPL37, RPL37A, RPL38, RPL39, RPLP0, RPLP1, RPLP2, RPL14, RPS14, POLR2H, POLR2J |
| **REACTOME METABOLISM OF MRNA** | 284 | Genes involved in Metabolism of mRNA | 5.72E-50 | RPS27A, RPS27, RPS6, RPS4X, RPSA, RPS2, RPS3, RPS5, RPS11, RPS12, RPS13, RPS15, RPS15A, RPS18, RPS19, RPS21, RPS24, RPS25, RPS26, RPS28, RPS29, UBA52, RPL23, RPL13A, RPL35, RPL36, RPL7, RPL8, RPL9, RPL10, RPL13, RPL15, RPL18, RPL18A, RPL24, RPL27A, RPL28, RPL29, RPL31, RPL32, RPL34, RPL35A, RPL37, RPL37A, RPL38, RPL39, RPLP0, RPLP1, RPLP2, RPL14, RPS14, PSMB4, PSMB5, EXOSC7, EXOSC3, EXOSC5, LSM2 |
| **REACTOME TRANSLATION** | 222 | Genes involved in Translation | 8.44E-48 | RPS27A, RPS27, RPS6, RPS4X, RPSA, RPS2, RPS3, RPS5, RPS11, RPS12, RPS13, RPS15, RPS15A, RPS18, RPS19, RPS21, RPS24, RPS25, RPS26, RPS28, RPS29, UBA52, RPL23, RPL13A, RPL35, RPL36, RPL7, RPL8, RPL9, RPL10, RPL13, RPL15, RPL18, RPL18A, RPL24, RPL27A, RPL28, RPL29, RPL31, RPL32, RPL34, RPL35A, RPL37, RPL37A, RPL38, RPL39, RPLP0, RPLP1, RPLP2, RPL14, RPS14 |
| **REACTOME METABOLISM OF RNA** | 330 | Genes involved in Metabolism of RNA | 3.59E-46 | RPS27A, RPS27, RPS6, RPS4X, RPSA, RPS2, RPS3, RPS5, RPS11, RPS12, RPS13, RPS15, RPS15A, RPS18, RPS19, RPS21, RPS24, RPS25, RPS26, RPS28, RPS29, UBA52, RPL23, RPL13A, RPL35, RPL36, RPL7, RPL8, RPL9, RPL10, RPL13, RPL15, RPL18, RPL18A, RPL24, RPL27A, RPL28, RPL29, RPL31, RPL32, RPL34, RPL35A, RPL37, RPL37A, RPL38, RPL39, RPLP0, RPLP1, RPLP2, RPL14, RPS14, PSMB4, PSMB5, EXOSC7, EXOSC3, EXOSC5, LSM2 |
| **REACTOME METABOLISM OF PROTEINS** | 518 | Genes involved in Metabolism of proteins | 1.35E-41 | RPS27A, RPS27, RPS6, RPS4X, RPSA, RPS2, RPS3, RPS5, RPS11, RPS12, RPS13, RPS15, RPS15A, RPS18, RPS19, RPS21, RPS24, RPS25, RPS26, RPS28, RPS29, UBA52, RPL23, RPL13A, RPL35, RPL36, RPL7, RPL8, RPL9, RPL10, RPL13, RPL15, RPL18, RPL18A, RPL24, RPL27A, RPL28, RPL29, RPL31, RPL32, RPL34, RPL35A, RPL37, RPL37A, RPL38, RPL39, RPLP0, RPLP1, RPLP2, RPL14, RPS14, SLC25A13, TIMM8A, TIMM13, TIMM10, GFER, PAM16, TOMM7, PMPCB, B4GALT3, PIGC, PIGQ, GALNT14 |
| **REACTOME FORMATION OF THE TERNARY COMPLEX AND SUBSEQUENTLY THE 43S COMPLEX** | 74 | Genes involved in Formation of the ternary complex, and subsequently, the 43S complex | 6.63E-23 | RPS27A, RPS27, RPS6, RPS4X, RPSA, RPS2, RPS3, RPS5, RPS11, RPS12, RPS13, RPS15, RPS15A, RPS18, RPS19, RPS21, RPS24, RPS25, RPS26, RPS28, RPS29, RPS14 |
| **REACTOME ACTIVATION OF THE MRNA UPON BINDING OF THE CAP BINDING COMPLEX AND EIFS AND SUBSEQUENT BINDING TO 43S** | 84 | Genes involved in Activation of the mRNA upon binding of the cap-binding complex and eIFs, and subsequent binding to 43S | 1.41E-21 | RPS27A, RPS27, RPS6, RPS4X, RPSA, RPS2, RPS3, RPS5, RPS11, RPS12, RPS13, RPS15, RPS15A, RPS18, RPS19, RPS21, RPS24, RPS25, RPS26, RPS28, RPS29, RPS14 |
| **KEGG HUNTINGTONS DISEASE** | 185 | Huntington's disease | 7.12E-07 | POLR2H, POLR2J, UQCR11, COX7C, UQCRQ, NDUFB9, NDUFB10, NDUFC1, ATP5D, ATP5O, ATP5G2, BBC3, AP2S1, DNAL4, CREB3L4 |
| **REACTOME MITOCHONDRIAL PROTEIN IMPORT** | 58 | Genes involved in Mitochondrial Protein Import | 3.81E-05 | SLC25A13, TIMM8A, TIMM13, TIMM10, GFER, PAM16, TOMM7, PMPCB |
| **KEGG PYRIMIDINE METABOLISM** | 98 | Pyrimidine metabolism | 2.13E-04 | POLR2H, POLR2J, POLR1C, POLR3K, ZNRD1, NME1, NME4, ITPA, NME3 |
| **REACTOME RESPIRATORY ELECTRON TRANSPORT ATP SYNTHESIS BY CHEMIOSMOTIC COUPLING AND HEAT PRODUCTION BY UNCOUPLING PROTEINS** | 98 | Genes involved in Respiratory electron transport, ATP synthesis by chemiosmotic coupling, and heat production by uncoupling proteins. | 2.13E-04 | UQCR11, COX7C, UQCRQ, NDUFB9, NDUFB10, NDUFC1, ATP5D, ATP5O, ATP5J2 |
| **KEGG PURINE METABOLISM** | 159 | Purine metabolism | 2.64E-04 | POLR2H, POLR2J, POLR1C, POLR3K, ZNRD1, NME1, NME4, ITPA, NME3, IMPDH2, APRT |
| **KEGG OXIDATIVE PHOSPHORYLATION** | 135 | Oxidative phosphorylation | 3.78E-04 | UQCR11, COX7C, UQCRQ, NDUFB9, NDUFB10, NDUFC1, ATP5D, ATP5O, ATP5G2, ATP5J2 |
| **KEGG ALZHEIMERS DISEASE** | 169 | Alzheimer's disease | 4.28E-04 | UQCR11, COX7C, UQCRQ, NDUFB9, NDUFB10, NDUFC1, ATP5D, ATP5O, ATP5G2, PSENEN, CACNA1D |
| **REACTOME TCA CYCLE AND RESPIRATORY ELECTRON TRANSPORT** | 141 | Genes involved in The citric acid (TCA) cycle and respiratory electron transport | 4.96E-04 | UQCR11, COX7C, UQCRQ, NDUFB9, NDUFB10, NDUFC1, ATP5D, ATP5O, ATP5J2, MDH2 |
| **REACTOME DNA REPAIR** | 112 | Genes involved in DNA Repair | 4.96E-04 | RPS27A, UBA52, POLR2H, POLR2J, RFC2, DDB2, FANCG, NTHL1, ALKBH2 |
| **REACTOME DNA REPLICATION** | 192 | Genes involved in DNA Replication | 1.21E-03 | RPS27A, RPS27, UBA52, PSMB4, PSMB5, RFC2, E2F1, MCM7, PMF1, CENPM, CENPT |
| **KEGG RNA POLYMERASE** | 29 | RNA polymerase | 1.21E-03 | POLR2H, POLR2J, POLR1C, POLR3K, ZNRD1 |
| **KEGG PARKINSONS DISEASE** | 133 | Parkinson's disease | 1.74E-03 | UQCR11, COX7C, UQCRQ, NDUFB9, NDUFB10, NDUFC1, ATP5D, ATP5O, ATP5G2 |
| **REACTOME MITOTIC M M G1 PHASES** | 172 | Genes involved in Mitotic M-M/G1 phases | 2.27E-03 | RPS27A, RPS27, UBA52, PSMB4, PSMB5, E2F1, MCM7, PMF1, CENPM, CENPT |
| **REACTOME CELL CYCLE** | 421 | Genes involved in Cell Cycle | 2.67E-03 | RPS27A, RPS27, UBA52, PSMB4, PSMB5, RFC2, E2F1, MCM7, PMF1, CENPM, CENPT, HIST1H2BD, HIST1H2BK, RAD9A, TERT, REC8 |
| **REACTOME GLYCEROPHOSPHOLIPID BIOSYNTHESIS** | 82 | Genes involved in Glycerophospholipid biosynthesis | 2.69E-03 | AGPAT2, GPAT2, CHKA, CRLS1, PTDSS2, LPIN3, DGAT1 |
| **REACTOME REGULATION OF MRNA STABILITY BY PROTEINS THAT BIND AU RICH ELEMENTS** | 84 | Genes involved in Regulation of mRNA Stability by Proteins that Bind AU-rich Elements | 3.03E-03 | RPS27A, UBA52, PSMB4, PSMB5, EXOSC7, EXOSC3, EXOSC5 |
| **REACTOME GENERIC TRANSCRIPTION PATHWAY** | 352 | Genes involved in Generic Transcription Pathway | 4.31E-03 | RPS27A, UBA52, KAT2A, NOTCH4, ZNF266, ZNF273, ZNF485, ZKSCAN5, ZNF500, ZNF3, ZNF138, ZNF655, ZNF394, ZNF496 |
| **REACTOME ASSEMBLY OF THE PRE REPLICATIVE COMPLEX** | 65 | Genes involved in Assembly of the pre-replicative complex | 5.36E-03 | RPS27A, UBA52, PSMB4, PSMB5, E2F1, MCM7 |
| **REACTOME DOWNSTREAM SIGNALING EVENTS OF B CELL RECEPTOR BCR** | 97 | Genes involved in Downstream Signaling Events Of B Cell Receptor (BCR) | 6.81E-03 | RPS27A, UBA52, PSMB4, PSMB5, NFKBIE, MLST8, AKT1S1 |
| **REACTOME CHOLESTEROL BIOSYNTHESIS** | 24 | Genes involved in Cholesterol biosynthesis | 6.81E-03 | EBP, LSS, TM7SF2, MVD |
| **KEGG P53 SIGNALING PATHWAY** | 69 | p53 signaling pathway | 6.81E-03 | BBC3, DDB2, CD82, PIDD, GADD45B, EI24 |
| **REACTOME HIV INFECTION** | 207 | Genes involved in HIV Infection | 7.79E-03 | RPS27A, UBA52, POLR2H, POLR2J, PSMB4, PSMB5, AP2S1, TCEB2, TAF11, AP1S1 |
| **REACTOME TRANSCRIPTION** | 210 | Genes involved in Transcription | 8.51E-03 | POLR2H, POLR2J, POLR1C, POLR3K, HIST1H2BD, HIST1H2BK, TCEB2, TAF11, TAF1C, PABPN1 |
| **REACTOME SIGNALING BY NOTCH** | 103 | Genes involved in Signaling by NOTCH | 8.57E-03 | RPS27A, UBA52, PSENEN, E2F1, KAT2A, NOTCH4, RFNG |
| **PID P53 DOWNSTREAM PATHWAY** | 137 | Direct p53 effectors | 8.65E-03 | BBC3, DDB2, E2F1, KAT2A, CD82, PIDD, TNFRSF10D, PCBP4 |
| **REACTOME MRNA DECAY BY 3 TO 5 EXORIBONUCLEASE** | 11 | Genes involved in mRNA Decay by 3' to 5' Exoribonuclease | 8.74E-03 | EXOSC7, EXOSC3, EXOSC5 |
| **KEGG GLYCEROPHOSPHOLIPID METABOLISM** | 77 | Glycerophospholipid metabolism | 1.06E-02 | AGPAT2, GPAT2, CHKA, CRLS1, PTDSS2, LCAT |
| **REACTOME RECEPTOR LIGAND BINDING INITIATES THE SECOND PROTEOLYTIC CLEAVAGE OF NOTCH RECEPTOR** | 12 | Genes involved in Receptor-ligand binding initiates the second proteolytic cleavage of Notch receptor | 1.10E-02 | RPS27A, UBA52, NOTCH4 |
| **REACTOME RESPIRATORY ELECTRON TRANSPORT** | 79 | Genes involved in Respiratory electron transport | 1.16E-02 | UQCR11, COX7C, UQCRQ, NDUFB9, NDUFB10, NDUFC1 |
| **REACTOME VIF MEDIATED DEGRADATION OF APOBEC3G** | 52 | Genes involved in Vif-mediated degradation of APOBEC3G | 1.18E-02 | RPS27A, UBA52, PSMB4, PSMB5, TCEB2 |
| **REACTOME M G1 TRANSITION** | 81 | Genes involved in M/G1 Transition | 1.26E-02 | RPS27A, UBA52, PSMB4, PSMB5, E2F1, MCM7 |
| **REACTOME ASSOCIATION OF LICENSING FACTORS WITH THE PRE REPLICATIVE COMPLEX** | 14 | Genes involved in Association of licensing factors with the pre-replicative complex | 1.63E-02 | RPS27A, UBA52, E2F1 |
| **REACTOME NRIF SIGNALS CELL DEATH FROM THE NUCLEUS** | 15 | Genes involved in NRIF signals cell death from the nucleus | 1.94E-02 | RPS27A, UBA52, PSENEN |
| **KEGG RNA DEGRADATION** | 59 | RNA degradation | 1.94E-02 | EXOSC7, EXOSC3, EXOSC5, LSM2, LSM7 |
| **REACTOME CELL CYCLE CHECKPOINTS** | 124 | Genes involved in Cell Cycle Checkpoints | 2.03E-02 | RPS27A, UBA52, PSMB4, PSMB5, RFC2, MCM7, RAD9A |
| **REACTOME ER PHAGOSOME PATHWAY** | 61 | Genes involved in ER-Phagosome pathway | 2.09E-02 | RPS27A, UBA52, PSMB4, PSMB5, HLA-F |
| **REACTOME SIGNALING BY THE B CELL RECEPTOR BCR** | 126 | Genes involved in Signaling by the B Cell Receptor (BCR) | 2.09E-02 | RPS27A, UBA52, PSMB4, PSMB5, NFKBIE, MLST8, AKT1S1 |

**Supplementary table 7. Overrepresented gene sets of cancer gene outliers**

| **Gene Set Name** | **# Genes in Gene Set (K)** | **Description** | **FDR q-value** | **Related Genes** |
| --- | --- | --- | --- | --- |
| KEGG RIBOSOME | 88 | Ribosome | 4.64E-16 | RPS6, RPL35, RPL22L1, RPL36, RPL4, RPL6, RPL13, RPL17, RPL18, RPL18A, RPL19, RPL22, RPL24, RPL26, RPL36AL, RPLP0, RPLP1, RPS2, RPS4X, RPS7, RPS12, RPS15, RPS18 |
| KEGG SPLICEOSOME | 128 | Spliceosome | 0.00000052 | LSM3, LSM2, HSPA6, PQBP1, USP39, PRPF6, SRSF7, SNRPA, SNRPA1, SNRPB2, SNRPC, SNRPD2, SF3A2, THOC3, DHX16, BUD31, RBM8A |
| KEGG RNA DEGRADATION | 59 | RNA degradation | 0.000343 | LSM3, LSM2, DCPS, EXOSC3, EXOSC4, EXOSC5, PAPOLG, SKIV2L, ZCCHC7 |
| KEGG CELL CYCLE | 128 | Cell cycle | 0.000343 | GADD45B, CDK6, CDKN1A, RBX1, HDAC2, ANAPC5, PCNA, CDK7, PKMYT1, MCM3, ORC5, WEE1, CDC45 |
| KEGG PYRIMIDINE METABOLISM | 98 | Pyrimidine metabolism | 0.000529 | POLR3F, POLR3D, POLR1C, ZNRD1, POLR1E, NUDT2, NME1, NME4, TK1, UPRT, AK3 |
| KEGG MAPK SIGNALING PATHWAY | 267 | MAPK signaling pathway | 0.0014 | HSPA6, GADD45B, PDGFB, TRAF2, FGF13, NFKB2, MAPK12, MAPK13, IL1B, PPP3R1, RRAS2, DAXX, HSPB1, CACNA2D3, CACNB4, RASGRP4, DUSP2, STK3 |
| KEGG PEROXISOME | 78 | Peroxisome | 0.00148 | DHRS4, IDH2, PRDX5, PEX1, PEX10, PEX14, PHYH, CROT, FAR2 |
| KEGG INSULIN SIGNALING PATHWAY | 137 | Insulin signaling pathway | 0.00148 | RPS6, PRKCI, TSC2, IRS1, IRS2, PRKAB1, FBP1, HK3, PRKAR2B, EIF4EBP1, SH2B2, FLOT2 |
| KEGG PATHWAYS IN CANCER | 328 | Pathways in cancer | 0.00148 | CDK6, CDKN1A, RBX1, HDAC2, PDGFB, TRAF2, FGF13, NFKB2, TCEB2, SLC2A1, VEGFA, EGLN3, ARNT2, TRAF3, RXRB, BCL2L1, LAMC3, TRAF1, PPARD, AXIN1 |
| KEGG ENDOCYTOSIS | 183 | Endocytosis | 0.00152 | HSPA6, PRKCI, SMURF1, CLTA, PIP5K1C, HLA-F, SRC, PARD3, PARD6A, GIT1, CHMP4B, RAB4A, TSG101, RAB11FIP3 |
| KEGG P53 SIGNALING PATHWAY | 69 | p53 signaling pathway | 0.00281 | GADD45B, CDK6, CDKN1A, TSC2, DDB2, RFWD2, CD82, SHISA5 |
| KEGG RENAL CELL CARCINOMA | 70 | Renal cell carcinoma | 0.00285 | RBX1, PDGFB, TCEB2, SLC2A1, VEGFA, EGLN3, ARNT2, PAK4 |
| KEGG RIG I LIKE RECEPTOR SIGNALING PATHWAY | 71 | RIG-I-like receptor signaling pathway | 0.00291 | TRAF2, MAPK12, MAPK13, TRAF3, IL12A, CASP10, DAK, TBKBP1 |
| KEGG UBIQUITIN MEDIATED PROTEOLYSIS | 138 | Ubiquitin mediated proteolysis | 0.00429 | RBX1, ANAPC5, TCEB2, SMURF1, DDB2, RFWD2, CUL4B, MGRN1, UBE2R2, UBE2A, CUL3 |
| KEGG RNA POLYMERASE | 29 | RNA polymerase | 0.00493 | POLR3F, POLR3D, POLR1C, ZNRD1, POLR1E |
| KEGG ABC TRANSPORTERS | 44 | ABC transporters | 0.00493 | ABCA3, ABCB7, ABCC1, ABCB1, ABCG2, ABCG1 |
| KEGG NUCLEOTIDE EXCISION REPAIR | 44 | Nucleotide excision repair | 0.00493 | RBX1, PCNA, CDK7, DDB2, CUL4B, CETN2 |
| KEGG PROTEASOME | 48 | Proteasome | 0.0075 | PSME1, PSME2, SHFM1, POMP, PSMB8, PSMC2 |
| KEGG ADIPOCYTOKINE SIGNALING PATHWAY | 67 | Adipocytokine signaling pathway | 0.00784 | TRAF2, IRS1, IRS2, PRKAB1, SLC2A1, RXRB, CAMKK2 |
| KEGG FRUCTOSE AND MANNOSE METABOLISM | 34 | Fructose and mannose metabolism | 0.00886 | FBP1, HK3, PFKL, TSTA3, PFKFB4 |
| KEGG PURINE METABOLISM | 159 | Purine metabolism | 0.00937 | POLR3F, POLR3D, POLR1C, ZNRD1, POLR1E, NUDT2, NME1, NME4, PRPS1, HPRT1, NUDT9 |
| KEGG HUNTINGTONS DISEASE | 185 | Huntington's disease | 0.00948 | HDAC2, CLTA, PLCB2, UQCRQ, NDUFB6, NDUFB9, NDUFB10, ATP5D, NDUFA4L2, SLC25A6, CREB3L4, HIP1 |
| KEGG LEISHMANIA INFECTION | 72 | Leishmania infection | 0.00995 | MAPK12, MAPK13, IL1B, IL12A, HLA-DMA, HLA-DQA2, NCF4 |
| KEGG INOSITOL PHOSPHATE METABOLISM | 54 | Inositol phosphate metabolism | 0.0106 | PIP5K1C, PLCB2, ITPKB, IMPA2, PI4KB, ISYNA1 |
| KEGG ALLOGRAFT REJECTION | 38 | Allograft rejection | 0.0119 | HLA-F, IL12A, HLA-DMA, HLA-DQA2, CD40 |
| KEGG ALZHEIMERS DISEASE | 169 | Alzheimer's disease | 0.0124 | IL1B, PPP3R1, PLCB2, UQCRQ, NDUFB6, NDUFB9, NDUFB10, ATP5D, NDUFA4L2, PSEN2, APBB1 |
| KEGG TOLL LIKE RECEPTOR SIGNALING PATHWAY | 102 | Toll-like receptor signaling pathway | 0.0157 | MAPK12, MAPK13, IL1B, TRAF3, IL12A, CD40, SPP1, TICAM1 |
| KEGG AXON GUIDANCE | 129 | Axon guidance | 0.0184 | PPP3R1, PAK4, L1CAM, EPHB2, EPHB4, ABLIM1, PLXNA1, SEMA3F, SRGAP3 |
| KEGG SMALL CELL LUNG CANCER | 84 | Small cell lung cancer | 0.0185 | CDK6, TRAF2, TRAF3, RXRB, BCL2L1, LAMC3, TRAF1 |
| KEGG PENTOSE PHOSPHATE PATHWAY | 27 | Pentose phosphate pathway | 0.0185 | FBP1, PFKL, PRPS1, TKT |
| KEGG TYPE I DIABETES MELLITUS | 44 | Type I diabetes mellitus | 0.0185 | IL1B, HLA-F, IL12A, HLA-DMA, HLA-DQA2 |
| KEGG RETINOL METABOLISM | 64 | Retinol metabolism | 0.019 | DHRS4, DGAT2, DGAT1, DHRS4L2, RDH11, DHRS3 |
| KEGG PARKINSONS DISEASE | 133 | Parkinson's disease | 0.0192 | UQCRQ, NDUFB6, NDUFB9, NDUFB10, ATP5D, NDUFA4L2, SLC25A6, UCHL1, SNCAIP |
| KEGG TIGHT JUNCTION | 134 | Tight junction | 0.0195 | RRAS2, PRKCI, SRC, PARD3, PARD6A, CSNK2B, MYH11, CLDN5, TJAP1 |
| KEGG APOPTOSIS | 88 | Apoptosis | 0.0203 | TRAF2, IL1B, PPP3R1, PRKAR2B, BCL2L1, CASP10, TNFRSF10D |
| KEGG REGULATION OF ACTIN CYTOSKELETON | 216 | Regulation of actin cytoskeleton | 0.0203 | PDGFB, FGF13, RRAS2, PIP5K1C, GIT1, PAK4, ITGB4, ARPC1B, ARPC1A, SSH3, ENAH, ARHGEF7 |
| KEGG NOTCH SIGNALING PATHWAY | 47 | Notch signaling pathway | 0.0203 | HDAC2, PSEN2, NOTCH4, MAML3, CIR1 |
| KEGG ANTIGEN PROCESSING AND PRESENTATION | 89 | Antigen processing and presentation | 0.0203 | HSPA6, HLA-F, PSME1, PSME2, HLA-DMA, HLA-DQA2, TAPBP |
| KEGG GLYCEROLIPID METABOLISM | 49 | Glycerolipid metabolism | 0.0236 | DAK, DGAT2, DGAT1, ALDH2, GPAT2 |
| KEGG GLYOXYLATE AND DICARBOXYLATE METABOLISM | 16 | Glyoxylate and dicarboxylate metabolism | 0.0242 | MDH1, MDH2, MTHFD2L |
| KEGG CITRATE CYCLE TCA CYCLE | 32 | Citrate cycle (TCA cycle) | 0.0258 | IDH2, MDH1, MDH2, SUCLG1 |
| KEGG ONE CARBON POOL BY FOLATE | 17 | One carbon pool by folate | 0.0272 | MTHFD2L, AMT, SHMT2 |
| KEGG MTOR SIGNALING PATHWAY | 52 | mTOR signaling pathway | 0.0272 | RPS6, TSC2, EIF4EBP1, VEGFA, ULK3 |
| KEGG LYSOSOME | 121 | Lysosome | 0.0272 | CLTA, HGSNAT, AP1S1, CTSD, GGA1, AP4M1, NAPSA, CD63 |
| KEGG AMYOTROPHIC LATERAL SCLEROSIS ALS | 53 | Amyotrophic lateral sclerosis (ALS) | 0.0285 | MAPK12, MAPK13, PPP3R1, DAXX, BCL2L1 |
| KEGG VEGF SIGNALING PATHWAY | 76 | VEGF signaling pathway | 0.0307 | MAPK12, MAPK13, PPP3R1, HSPB1, VEGFA, SRC |
| KEGG CYTOSOLIC DNA SENSING PATHWAY | 56 | Cytosolic DNA-sensing pathway | 0.0344 | POLR3F, POLR3D, POLR1C, IL1B, AIM2 |
| KEGG OXIDATIVE PHOSPHORYLATION | 135 | Oxidative phosphorylation | 0.0467 | UQCRQ, NDUFB6, NDUFB9, NDUFB10, ATP5D, NDUFA4L2, COX17, ATP5I |
| KEGG PYRUVATE METABOLISM | 40 | Pyruvate metabolism | 0.0476 | ALDH2, MDH1, MDH2, HAGHL |
| KEGG GLYCOLYSIS GLUCONEOGENESIS | 62 | Glycolysis / Gluconeogenesis | 0.0491 | FBP1, HK3, PFKL, ALDH2, PGK1 |
